# Synthesis of 2‐Alkynoates by Palladium(II)‐Catalyzed Oxidative Carbonylation of Terminal Alkynes and Alcohols

**DOI:** 10.1002/chem.201602558

**Published:** 2016-07-19

**Authors:** Qun Cao, N. Louise Hughes, Mark J. Muldoon

**Affiliations:** ^1^ School of Chemistry and Chemical EngineeringQueen's University BelfastStranmillis RoadBelfastBT9 5AGNorthern Ireland

**Keywords:** alkynes, carbonylation, homogeneous catalysis, oxidation, palladium

## Abstract

A homogeneous Pd^II^ catalyst, utilizing a simple and inexpensive amine ligand (TMEDA), allows 2‐alkynoates to be prepared in high yields by an oxidative carbonylation of terminal alkynes and alcohols. The catalyst system overcomes many of the limitations of previous palladium carbonylation catalysts. It has an increased substrate scope, avoids large excesses of alcohol substrate and uses a desirable solvent. The catalyst employs oxygen as the terminal oxidant and can be operated under safer gas mixtures.

2‐Alkynoates (alkynoate esters) are incredibly valuable building blocks for organic synthesis as they can be transformed into a diverse range of other desirable products.^1^ Unfortunately the synthesis of 2‐alkynoates often has several drawbacks, with only a limited number of synthetic methods for preparing these esters. For example, lithiated alkynes can be treated with an alkyl chloroformate,^2^, ^3^ and alkynyl carboxylic acids can be esterified using carbodiimide coupling reagents such as DCC^4^ and EDCI (Figure 1).^5^ Such methods utilize stoichiometric reagents and also have substrate limitations. 2‐Alkynoates can also be prepared catalytically with alkynes, CO_2_, and alkyl halides.^6^ Whilst the use of CO_2_ is desirable, employing alkyl halides can have disadvantages and there has been a limited substrate scope demonstrated to‐date using this route. An alternative is to carry out a catalytic oxidative carbonylation of terminal alkynes and alcohols. Replacing alkyl halides with alcohols is preferable, as a wide range of alcohols are commercially available and are normally inexpensive. Carbon monoxide is a widely available, sustainable, and inexpensive carbonyl source. Palladium catalyzed carbonylations are well established, however oxidative carbonylations have been less developed to‐date.^7^ This underdevelopment is apparent in the case of 2‐alkynoates. The oxidative carbonylation of alkynes with alcohols using Pd^II^ catalysts dates back some time, however, reactions do not always produce 2‐alkynoates and in some cases dicarbonylation is prevalent and a range of products can be produced.^8^ In terms of catalyst systems that can prepare 2‐alkynoates, there have only been a few reports using homogeneous Pd^II^ catalysts,^9^, ^10^, ^11^, ^12^, ^13^ and there are some significant limitations. In these examples, the catalyst loadings are high and there is only one example of O_2_ being used to directly reoxidize the catalyst.^13^ The use of only a limited number of substrates has been demonstrated so far and simple alcohols (such as methanol) have been the main focus. Furthermore, the alcohol is generally used as the solvent and when this is not the case it is used in very large excess.

**Figure 1:**
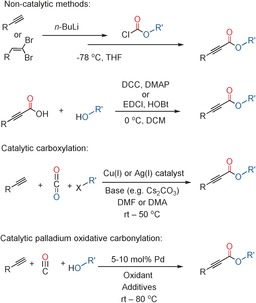
Examples of methods for the preparation of 2‐alkynoates.

More recently, an aerobic system which used a heterogeneous palladium catalyst (Pd on carbon) was reported.^14^ High yields of the desired 2‐alkynoate could be produced when 1,4‐dioxane was used as the solvent (THF was the next best solvent). A number of alkynes were shown to be suitable, but only primary alcohols acted as suitable nucleophiles. An attractive feature of this system is the ability to recycle the catalyst, however the inability to oxidize secondary alcohols and the use of 1,4‐dioxane as the solvent are significant drawbacks.

Our aim was to try and address the limitations of previous catalyst systems and develop a more efficient and widely applicable method. We are interested in exploiting ligands to improve the performance of Pd^II^‐catalyzed oxidation reactions, and developing systems which use sustainable oxidants such as O_2_ and H_2_O_2_.^15^ Ligand modulation has been successfully developed for other Pd^II^‐catalyzed reactions,^16^ but as Beller and co‐workers highlighted in their recent review,7c this is an area that needs to be addressed in the field of oxidative carbonylations. Another aim was to demonstrate that these aerobic systems could be operated under safer oxygen concentrations and with a safer solvent.

We screened a wide variety of ligands and other reaction conditions and a comprehensive summary of these details can be found in the Supporting Information. It was found that adding tetrabutylammonium iodide (TBAI), an additive previously employed in these reactions,^14^ was essential for good performance. In terms of solvents, we were pleased to find that ethyl acetate was the best solvent for these reactions. Ethyl acetate is a solvent which is a “recommended” choice by pharmaceutical companies,^17^ unlike solvents such as DMF or 1,4‐dioxane and THF, which have previously been used for these reactions. DMF and 1,4‐dioxane are classed as hazardous and are to be avoided, while THF is “problematic”.^17^ Furthermore, aerobic reactions in ethereal solvents such as 1,4‐dioxane and THF are particularly dangerous due to their propensity to form potentially explosive peroxides. It is possible that peroxide formation in 1,4‐dioxane plays a role in the catalysis, as this solvent has not only been used for oxidative synthesis of 2‐alkynoates, but it has also been the solvent of choice for oxidative aminocarbonylations to synthesize 2‐ynamides.^18^ There are examples of other palladium oxidation catalysts, in which in situ formation of peroxides in THF^19^ or 1,4‐dioxane^20^ are responsible for the reactivity. One would assume that the dangers of peroxide‐forming solvents would severely limit the use of these catalyst systems on a larger scale.

Additionally, we screened a range of ligands, which had a significant effect on the substrate conversion and selectivity to the desired 2‐alkynoates. Table 1 shows a few examples and further details are in the Supporting Information.

**Table 1:** Some examples of the ligand effects on the yield of respective 2‐alkynoates.

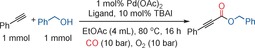			
Entry	Ligand	Amount [mol %]	Yield [%]^[a]^
1	–	–	10
2	PPh_3_	2	20
3	Phen	1	32
4	NEt_3_	20	19
5	TMDAM	10	12
**6**	**TMEDA**	**10**	**73**
7	TMPDA	10	18
8	TMBDA	10	11

[a] The yields were determined by GC using an internal standard. Experiment details and further examples of optimizations can be found in the Supporting Information.

To‐date the only example of a Pd^II^ catalyst that directly uses O_2_, is based on PPh_3_ as a ligand.^13^ However, phosphine ligands are not ideal candidates for oxidation reactions and it can be seen from our screening studies that other ligands delivered superior performances. The best overall performance in terms of rate and selectivity was obtained with *N,N,N′,N′*‐tetramethylethylenediamine (TMEDA) (Entry 6 in Table 1). After optimization, we found that a ratio of 10:1 TMEDA/Pd was optimal. The performance of TMEDA was very pleasing due to the fact that this is a readily available and very inexpensive amine ligand. Interestingly, the ethylene spacer in TMEDA was very important and having a methylene (TMDAM), propylene (TMPDA), or butylene (TMBDA) spacer did not result in high selectivity to the desired product.

We utilized Pd(OAc)_2_ as the palladium source and as shown in Table 2, found that the counter ion was very important. Successful catalysis was only obtained with alkyl carboxylate anions and there are a few possible reasons for this. Acetate (and similar carboxylate anions) are known to play a key role in C‐H activation reactions,^21^ and acetate effects have also been shown for the Pd^II^‐catalyzed hydration and dimerization reactions of terminal alkynes.^22^ Although some of the homogeneous systems that were previously reported used PdCl_2_ and PdBr_2_ salts, these methods also added NaOAc to the reaction.^9^, ^10^ However, when alcohols are replaced with amines in these oxidative carbonylation reactions to produce 2‐ynamides, there is not a need for a source of acetate.^18^ Therefore it is possible that the need for a carboxylate anion is related to its ability to deprotonate the Pd^II^‐coordinated alcohol. In previous studies on aerobic Pd^II^‐catalyzed oxidations of alcohols, it was found that acetate is often key in this regard.^23^

**Table 2:** Influence of different Pd^II^ salts on the catalytic performance.

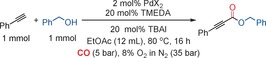			
Counterion *X*	Conv. alkyne [%]^[a]^	Conv. alcohol [%]^[a]^	Yield [%]^[a]^
I^−^	6	0	0
Cl^−^	12	0	0
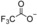	27	5	0
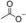	92	89	82
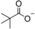	90	87	84
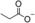	80	89	74

[a] Conversions and yields were determined by GC using an internal standard.

In terms of gas compositions, we can manage the dangers of using pressurized O_2_ or air on a small scale in our laboratory, and although we used such gas mixtures in our optimization experiments (see the Supporting Information), we wanted to demonstrate that the reactions could be carried out under safer conditions. Carbon monoxide is a flammable gas and pure CO/O_2_ mixtures are within the explosion limits as the lower flammability limit (LFL) of carbon monoxide in air is 11.5 vol % at 100 °C.^24^ Additionally, we also need to take into account that we are using organic solvents and those have their own flammability limits, so it is important to try and use limiting oxygen concentrations (LOC) when using batch conditions. The LOC of ethyl acetate at 100 °C is 9.4 vol % O_2_ at a pressure of 1 bar and 9.9 vol % O_2_ at a pressure of 20 bar.^25^ Therefore we carried out our substrate‐scope studies using 5 bar of CO with 35 bar of an O_2_/N_2_ gas mixture (8:92). By using an O_2_/N_2_ gas mixture we should not only be below the LOC of the solvent but also the LFL of CO. A lower catalyst loading could be employed with 35 bar of air (see the Supporting Information for details), but we wanted to demonstrate the use of a gas mixture that should be safe with regards to the solvent and CO.

With optimized conditions in hand, we demonstrated that we could produce a significant number of 2‐alkynoates (**1–33**), many of which in excellent yields. We examined a number of different primary alcohols using phenylacetylene as the alkyne (Figure 2) and a number of alkynes using benzyl alcohol as the nucleophile (Figure 3). In previous reports, a large excess of alcohol is normally required, but in the case of primary alcohols we could employ equimolar quantities of alkyne and alcohol. As shown in Figure 2, we could utilize both activated and unactivated alcohols. Substrates with electron‐withdrawing or ‐donating substituents proceeded readily. Heteroatoms were tolerated, although a lower yield was obtained with a pyridine‐containing substrate (**11**). Benzyl alcohols bearing chloro and bromo substituents (**6** and **7**) worked well, as did an aliphatic substrate bearing a terminal olefin (**16**). Such products have potential to be further functionalized at a later stage. Interestingly, the same substituents had a differing effect on the alkyne and the alcohol. For examples, compare **3** to **18** and **7** to **23**.

**Figure 2:**
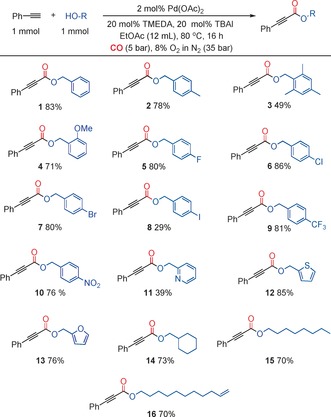
Synthesis of 2‐alkynoates with various primary alcohols.

**Figure 3:**
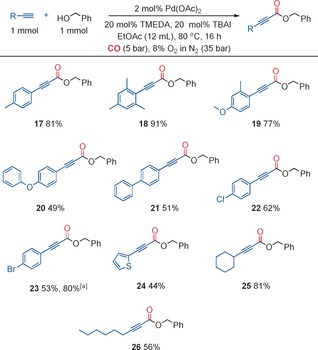
Synthesis of 2‐alkynoates with various alkynes. [a] 2 mmol benzyl alcohol.

Secondary alcohols are more challenging substrates, which is demonstrated by the fact that there are just a few examples in the literature that are restricted to simple substrates such as 2‐propanol. We found that we could utilize secondary alcohols with only a slight change to our method, employing a slightly higher catalyst loading (3 mol % Pd(OAc)_2_) and two equivalents of the alcohol (Figure 4). In the case of chiral alcohols, we demonstrated that the corresponding alkynoates could be produced with the chirality maintained. Previously, chiral 2‐alkynoates have been used for Pauson–Khand reactions.^4^ In this case, the 2‐alkynoates were prepared by more traditional methods using alkynyl carboxylic acid derivatives, and in a number of cases these methods were unable to produce the desired 2‐alkynoate.

**Figure 4:**
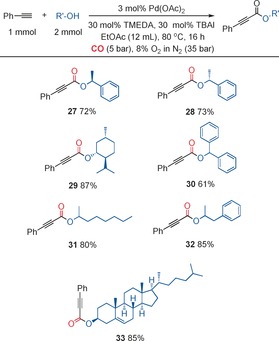
Synthesis of 2‐alkynoates with secondary alcohols.

In conclusion, our study has made significant strides towards developing efficient and scalable oxidative carbonylation methods for the synthesis of 2‐alkynoates. By studying a wide range of variables it has become clear that these reactions are complex and a number of factors greatly influence their performance. This highlights the need for a greater mechanistic understanding of Pd^II^‐catalyzed oxidative carbonylation reactions; something which is currently lacking in this area. We have demonstrated that ligands can have a profound effect on these reactions and pleasingly TMEDA, a very inexpensive amine, was found to deliver an excellent performance. The catalyst has a wider/more diverse substrate scope than previous examples and can utilize O_2_ to directly reoxidize the catalyst. The system utilizes ethyl acetate, an industrially preferred solvent, and does not require large excess of the alcohol substrates. The work also highlights the importance of using safer gas mixtures to avoid potentially explosive conditions. Further work will examine if we can improve the performance of a wider range of oxidative carbonylation reactions.

## Supporting information

As a service to our authors and readers, this journal provides supporting information supplied by the authors. Such materials are peer reviewed and may be re‐organized for online delivery, but are not copy‐edited or typeset. Technical support issues arising from supporting information (other than missing files) should be addressed to the authors.

Supplementary
