# Supplementary material for: Synthesis of 2‐Alkynoates by Palladium(II)‐Catalyzed Oxidative Carbonylation of Terminal Alkynes and Alcohols
Source: Chemistry. 2016 Jul 19;22(34):11982–5. doi: 10.1002/chem.201602558 (PMC5347984; doi:10.1002/chem.201602558)
Supplement: Supplementary file 1 — Supplementary [file CHEM-22-11982-s001.pdf]

CHEMISTRY
A European Journal

Supporting Information

Synthesis of 2-Alkynoates by Palladium(II)-Catalyzed Oxidative Carbonylation of Terminal Alkynes and Alcohols

Qun Cao, N. Louise Hughes, and Mark J. Muldoon*[a]

chem_201602558_sm_miscellaneous_information.pdf

1 General Information

Unless otherwise stated, all reagents were purchased from Sigma-Aldrich and used without further purification. The following chemicals were purchased from Fluorochem: (4-chlorophenyl)methanol, (4-iodophenyl)methanol, 4-ethynyl-1,1'-biphenyl, 4-ethynylbenzonitrile, 1-chloro-4-ethynylbenzene, 1-bromo-4-ethynylbenzene, 2-ethynylthiophene, palladium(II) acetate (99%). The following palladium salts were purchased from Alfa Aesar: palladium(II) trifluoroacetate (97%), palladium(II) trimethylacetate (97%). The following palladium salts were purchased from Sigma Aldrich: palladium(II) propionate (≥ 99.5%), palladium(II) acetate (≥99.9 trace metal basis), palladium(II) chloride (≥99.9%), palladium(II) iodide (≥99.9%). TMEDA used for the synthesis of 2-alkynoates was purchased from Sigma Aldrich (≥99.5%).

Thin layer chromatography (TLC) was carried out using Merck TLC silica gel 60 sheets, and visualized with ultraviolet light or potassium permanganate stain. Flash column chromatography (FCC) was performed with Fluorochem silica gel 60 Å as the stationary phase and solvents employed were analytical grade. ^{1}H NMR spectra were recorded on a Bruker AVX400 (400 MHz) spectrometer at ambient temperature. ^{13}C NMR spectra were recorded on a Bruker AVX400 (100 MHz) spectrometer at ambient temperature. Mass spectra （ESI) data were analyzed using Waters LCT Premier TOF. Melting points were measured on Stuart melting point apparatus (Digital, SMP10). IR spectra were measured on PerkinElmer Spectrum 100 FT-IR Spectrometers. Carbon monoxide (CP Grade), air and O_2 cylinders were from BOC and pre-mixed O_2:N_2(8:92) (β standard) cylinder was from BOC Special Gases.

Chiral HPLC was carried out on an Agilent 1100 series HPLC, with a Chiralcel® OJ-H column. HPLC conditions: 1mL/min 95% hexane / 5% isopropanol with a diode array detector measuring at 220 nm.

Gas chromatography analysis was carried out using Agilent 7820A series gas chromatograph. An Agilent 19091J-413HP-5 column (30.0 m × 320 µm × 0.25 µm nominal) was employed for all the separations using the following conditions: initial column temperature, 40 °C; initial hold time, 1 min; next temperature, 100 °C; hold time, 5 min; rate of temperature ramp 1, 4 °C/min, final temperature 320 °C; hold time, 5 min; rate of temperature ramp 2, 30 °C/min; injection temperature, 250 °C; injection volume 1 µL; detection temperature, 300 °C, split mode. The effluent was combusted in an H_2/air flame and detected using FID (flame ionization detector).

The GC yield of products and conversion of substrates were determined by using the internal standard method. The response factor (RF) of analytes was determined by analyzing known quantities of internal standard (biphenyl) against known quantities of substrate and product:

$$RF = \frac{Area_{internal\ standard} \times Moles_{analyte}}{Area_{analyte} \times Moles_{internal\ standard}}$$

The quantity of an analyte was then calculated according to the following equation:

$$Moles_{analyte} = \frac{RF \times Moles_{internal\ standard} \times Area_{analyte}}{Area_{internal\ standard}}$$

2 Experimental Procedures

Safety

Catalytic oxidative carbonylations should be carried out by trained personnel, with suitable safety measures and utilizing appropriate equipment. Suitable precautions should be taken by those wishing to reproduce or extend this type of work.

In these studies high pressure O_2 gas mixtures (air and 8% O_2) and pure O_2 are employed with organic solvents and CO. We use pressures which are significantly below the pressure ratings of the vessels, and these are also equipped with safety relief valves (set to release pressure at 100 bar). Carbon monoxide is a flammable and highly toxic gas. The CO cylinder was stored in a ventilated cylinder cupboard adjacent to the fume hood. A CO monitor / alarm was used in order to detect any leaks. Pressurized tubing and reactors were all vented in the fume hood.

General points:

$Pd(OAc)_2$ which was ≥99.9% trace metal basis purity (from Sigma Aldrich) was used and it was found that lower grades of $Pd(OAc)_2$ led to reduced yields (See Table S5 for an example).

In the case of TMEDA, stock solutions were made and these were used immediately and only used once. It was found that storing and using the stock solution over multiple days led to reduced product yield.

Reactions were all carried out in pressure vessels which were heated and stirred on a hotplate stirrer. Reactions were carried out in glass liners and stirred using Teflon coated magnetic stirrer bars. If reactions were carried out without using a glass liner, it was found that the stainless steel or Hastelloy C276 reactor body caused a dramatic reduction in the yield of product produced (and selectivity).

2.1 General Method A: procedure for evaluation of ligands, solvent, additives and temperature

Reactions were carried out in 16 mL high-pressure reactors made of stainless steel or Hastelloy C276 and the reaction mixture was placed in a glass liner along with a magnetic stirrer bar.

Into a glass liner, pre-made* Pd(II) complex (1 mol%, 0.01 mmol), tetrabutylammonium iodide (TBAI) (10 mol%, 0.1 mmol, 0.0369 g) and internal standard biphenyl (~ 0.3 g), a magnetic stir bar in 4 mL of ethyl acetate were added. Then substrate phenylacetylene (1 mmol, 0.1021 g) and benzyl alcohol (1mmol, 0.1080 g) were then added. The glass liner was then placed in the reactor, which was then pressurized to 10 bar with carbon monoxide gas, followed by pressuring to a total pressure of 20 bar with oxygen gas to make a $CO:O_2$ = 1:1 gas mixture. The reactor was then stirred in a heating block (on a hotplate stirrer) at 80 °C for 16 hours. Reactions were sampled after the reactor body was cooled in an ice bath and slowly depressurized. The collected sample was washed through a silica plug with diethyl ether to remove all catalyst components. Filtrate was collected and submitted for GC analysis.

*For non-premade catalysts, $Pd(OAc)_2$ (1 mol%, 0.01 mmol, 0.0022 g) and ligand (1 mol%, 0.01 mmol *unless specified*) were added into the glass liner in 4 mL of ethyl acetate. Then the glass liner was put into a water bath sonicator for 1 min to solubilize the mixture, before adding biphenyl (internal standard), tetrabutylammonium iodide and substrates.

2.2 General Method B: procedure for the synthesis of 2-alkynoates using primary alcohols

Reactions were carried out in 45 mL high-pressure reactors made of stainless steel and the reaction mixture was placed in a glass liner along with a magnetic stirrer bar.

To the glass liner, $Pd(OAc)_2$ (2 mol%, 0.02 mmol, 0.0046 g) and TMEDA (20 mol%, 0.2 mmol, 0.0238 g) from a stock solution in ethyl acetate were added with a magnetic stir bar and more ethyl acetate was added to give a final volume of 12 mL. Then the glass liner was put into a water bath sonicator for 1 min to solubilize the mixture. Then tetrabutylammonium iodide (20 mol%, 0.2 mmol, 0.0748 g) was added followed by alkyne (1 mmol) and alcohol (1 mmol). Then the reactor was pressurized with 5 bar of carbon monoxide gas, followed by O_2:N_2 (8:92) to make a total pressure of 40 bar. The reactor was then stirred in a heating block at 80 °C for 16 hours. When reaction was finished, the reaction mixture was transferred to a separating funnel and brine (20 mL) was added. The aqueous layer was separated and back extracted with diethyl ether or ethyl acetate (30 mL) twice. Then the combined organic layers were dried over $MgSO_4$, filtered and concentrated under reduced pressure. The crude material was then purified by silica gel flash chromatography, then the product containing fractions were combined and concentrated under reduced pressure.

2.3 General Method C: Procedure for the synthesis of 2-alkynoates with secondary alcohols

Procedure was the same as described above in General Method B but using 3 mol%, $Pd(OAc)_2$, 30 mol% TMEDA, 30 mol% tetrabutylammonium iodide and 2 mmol of alcohol.

3 Ligand screening and reaction optimization

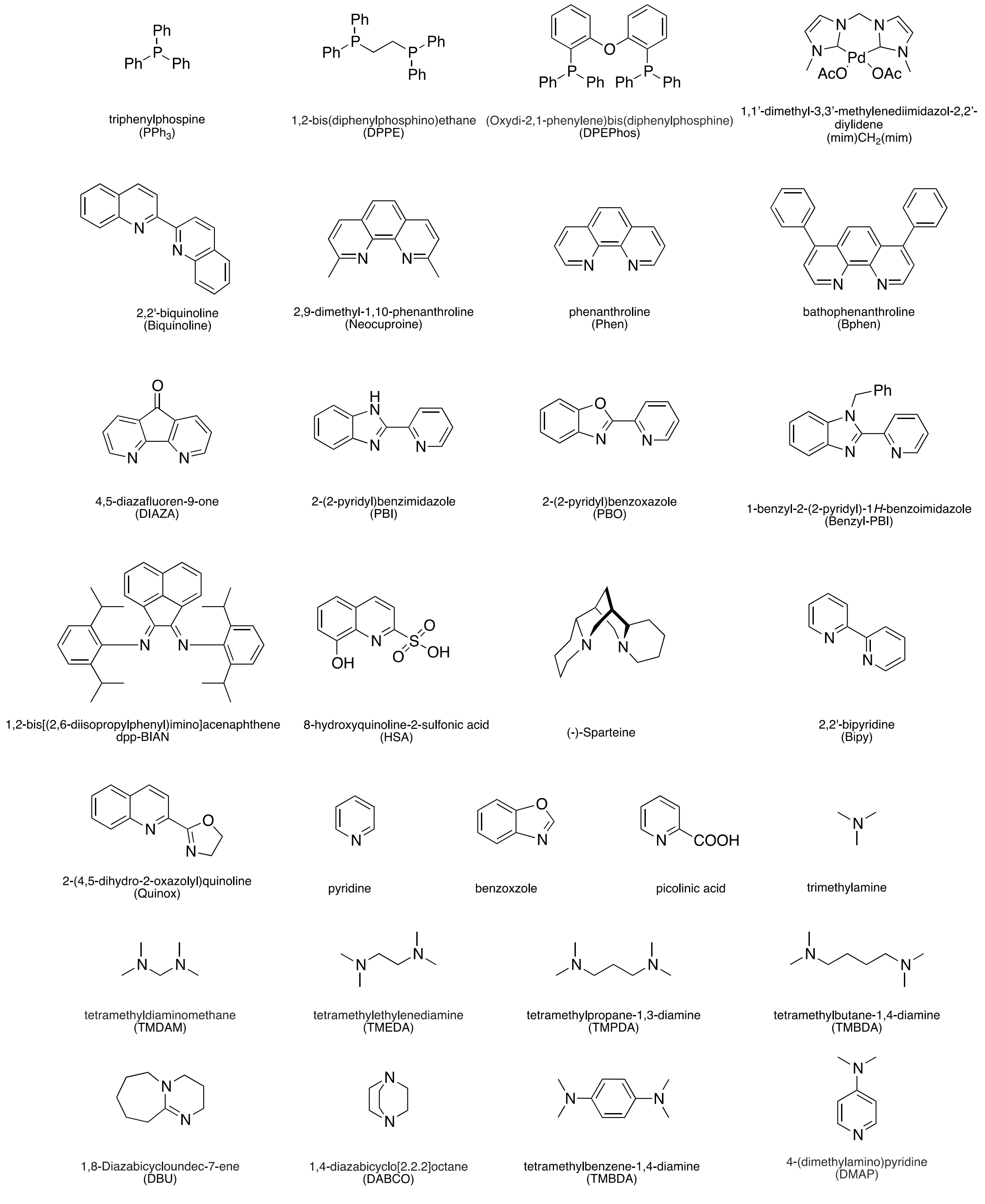

Figure S1: Structures and acronyms of ligands that were tested for the oxidative carbonylation for the synthesis of 2-alkynoates.

Table S1: Screening of phosphorus ligands for the synthesis of 2-alkynoates

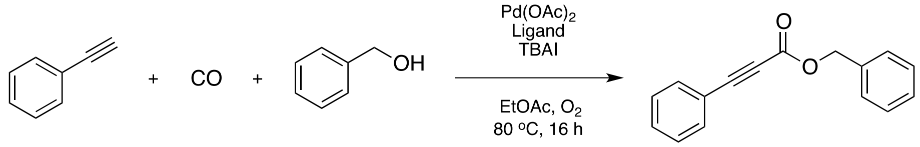

Entry	$Pd(OAc)_2$	Ligand	TBAI	Alkyn Conv. [%][a]	Alcohol Cov. [%][a]	Yield [%][a]
1	5 mol%	10 mol% PPh_3	N/A	60	26	17
2	5 mol%	5 mol% DPPE	N/A	100	25	12
3	5 mol%	5 mol% DPEPhose	N/A	36	6	6
4	5 mol%	10 mol% PPh_3	30 mol%	97	59	55
5	5 mol%	5 mol% DPPE	30 mol%	75	69	62
6	5 mol%	5 mol% DPEPhose	30 mol%	77	46	34

Reaction conditions: $Pd(OAc)_2$, Ligand, phenylacetylene (1 mmol, 0.1020 g), benzyl alcohol (1 mmol, 0.1080 g), ethyl acetate (4 mL), CO/O_2 = 1:1 (20 bar), 80 °C, 16 hours. Experimant details see General Method A.
[a] Conversion and yield were determined by GC using biphenyl as internal standard

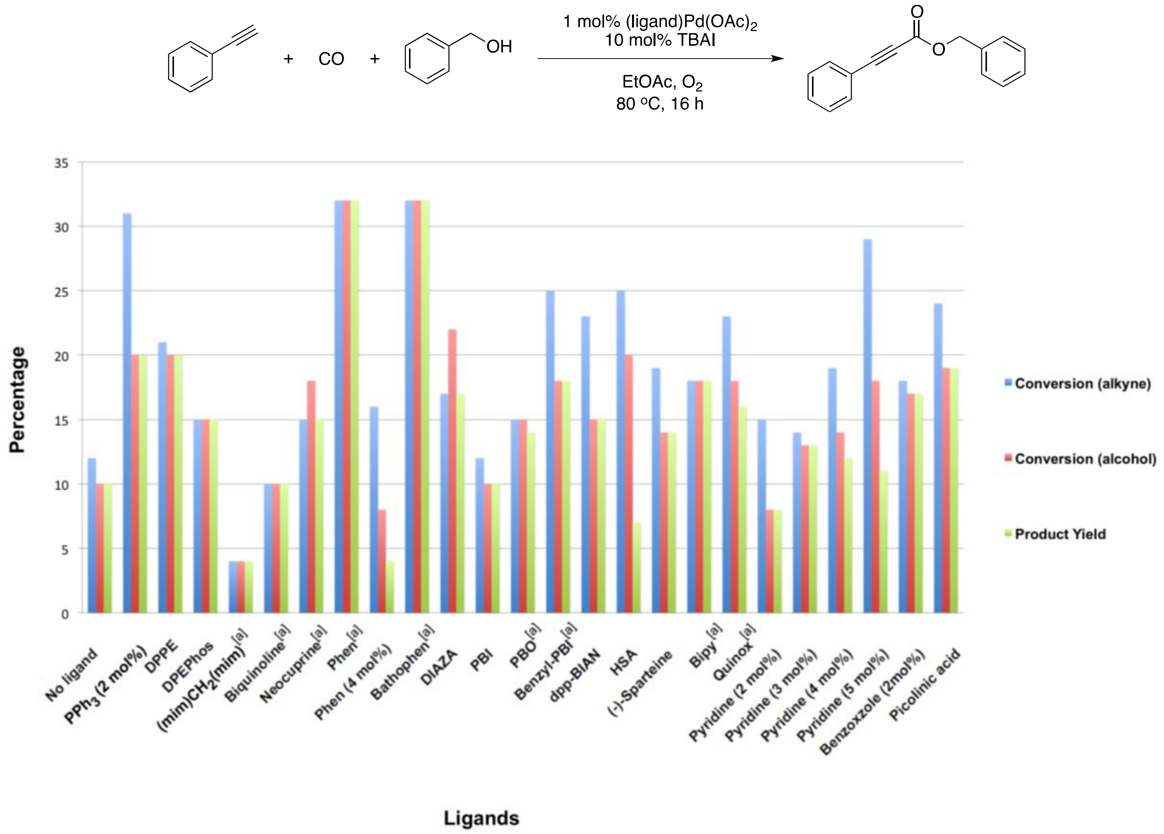

Figure S2: Initial evaluation of ligands for the oxidative carbonylation for the synthesis of 2-alkynoates. For experiment details see General Method A. Ligand structures see Figure S1.

[a] Pre-made & isolated (Ligand)Pd$(OAc)_2$ was used.

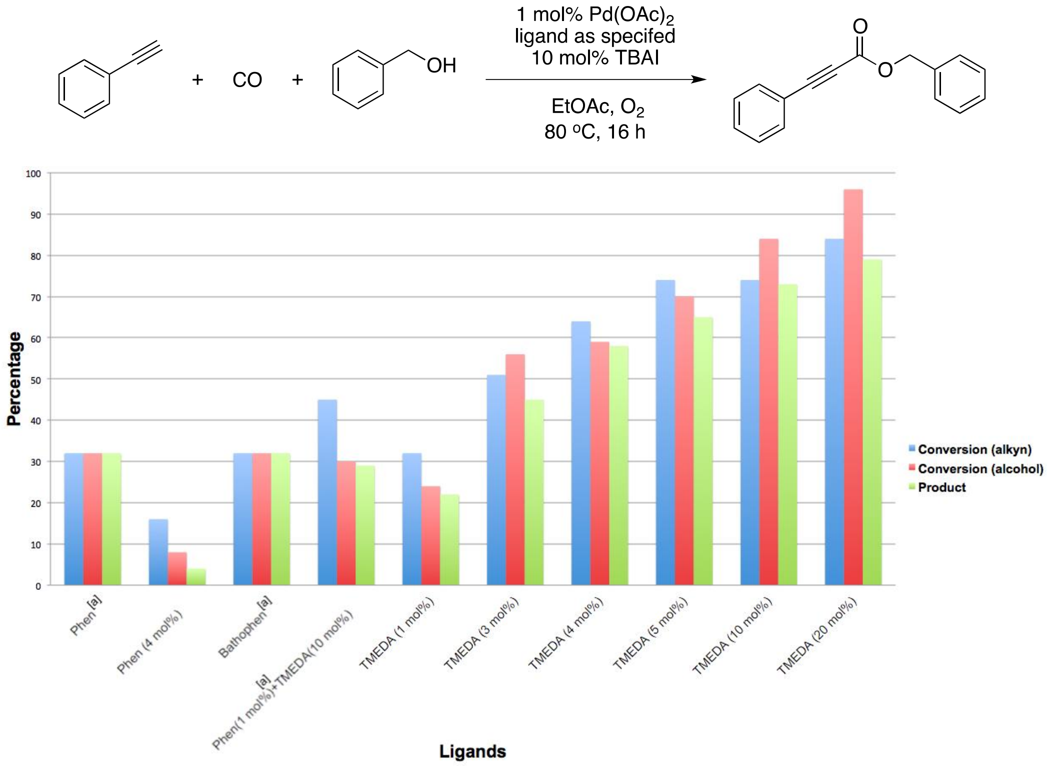

Figure S3: Using TMEDA as ligand for the synthesis of 2-alkynoates.

For experiment details see General Method A. Ligand structures see Figure S1.

[a] Pre-made & isolated (Ligand)Pd$(OAc)_2$ was used.

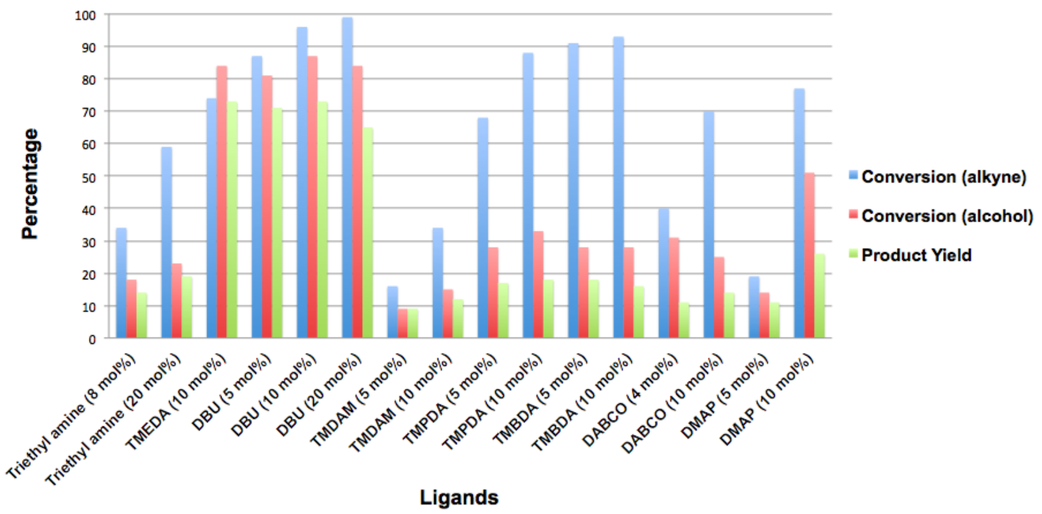

Figure S4: Amine ligand screening for the synthesis of 2-alkynoates.

For experiment details see General Method A. Ligand structures see Figure S1.

[a] Pre-made & isolated (Ligand)Pd$(OAc)_2$ was used.

Note: Increasing TMEDA loading from 10 mol% to 20 mol% with 1 mol% Pd(II) showed better results when CO/O_2 = 1:1 (20 bar) was used. When 5 bar CO with 35 bar of an O_2:N_2 (8:92) gas mixture was used, increasing TMEDA amount from 20 mol% to 40 mol% with 2 mol% Pd(II) did not improve yield of 2-alkynoates (Experiment details see Table S12, Entry 10)

Table S2: Evaluation of additives for oxidative carbonylation for the synthesis of 2-alkynoate with $(Phen)Pd(OAc)_2$ as catalyst

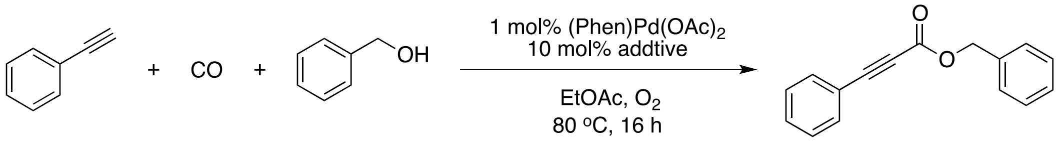

Entry	Additive	Alkyn Conv. [%][a]	Alcohol Conv. [%][a]	Yield [%][a]
1[b]	N/A	40	6	5
2	$[NBu_4]I$	32	32	32
3	$[NBu_4]Br$	52	16	12
4	$[NBu_4][OAc]$	15	5	2
5	KI	35	2	2

Reaction conditions: 1 mol% $(Phen)Pd(OAc)_2$ (0.01 mmol, 0.0040 g), Additive as specified (10 mol%), phenylacetylene (1 mmol, 0.1020 g), benzyl alcohol (1 mmol, 0.1080 g), EtOAc (4 mL), CO/O_2 = 1:1 (20 bar), 80 °C, 16 hours. Experimant details see General Method A.
[a] Conversion and yield were determined by GC using biphenyl as internal standard
[b] No additive was added

Table S3: Optimization of TBAI for oxidative carbonylation of alkyne and alcohol with CO for the synthesis of 2-alkynoate using $(Phen)Pd(OAc)_2$ as catalyst

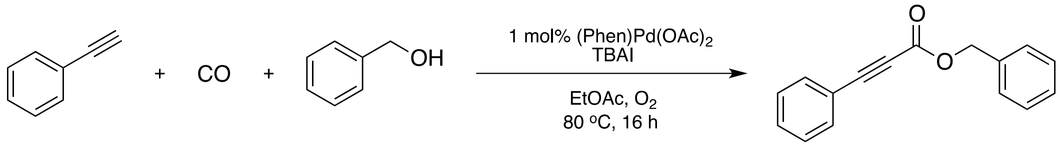

Entry	TBAI	Alkyn Conv. [%][a]	Alcohol Conv. [%][a]	Yield [%][a]
1	N/A	40	6	5
2	1 mol%	27	9	8
3	2 mol%	20	13	12
4	6 mol%	25	25	25
5	10 mol%	32	32	32
6	20 mol%	24	30	24

Reaction conditions: 1 mol% $(Phen)Pd(OAc)_2$ (0.01 mmol, 0.0040 g), amount of TBAI as specified, phenylacetylene (1 mmol, 0.1020 g), benzyl alcohol (1 mmol, 0.1080 g), ethyl acetate (4 mL), CO/O_2 = 1:1 (20 bar), 80 °C, 16 hours. Experimant details see General Method A.
[a] Conversion and yield were determined by GC using biphenyl as internal standard

Table S4: Evaluation of counter ion of different (phenanthroline)palladium complexes

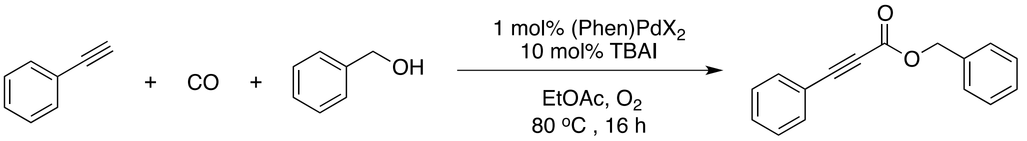

Entry	Catalyst	Conv. alkyn [%][a]	Conv. alcohol [%][a]	Yield of product [%][a]
1	(Phen)Pd(OAc)$_2$	32	32	32
2	(Phen)Pd(MeCN)$_2$(OTf)$_2$	0	0	0
3	(Phen)Pd(CF$_3$COO)$_2$	0	0	0
4	(Phen)PdI$_2$	0	0	0

Reaction conditions: 1 mol% (Phen)PdX$_2$ (0.01 mmol), 10 mol% TBAI (0.1 mmol, 0.0374 g), phenylacetylene (1 mmol, 0.1020 g), benzyl alcohol (1 mmol, 0.1080 g), ethyl acetate (4 mL), CO/O$_2$ = 1:1 (20 bar), 80 °C, 16 hours. Experimant details see General Method A.
[a] Conversion and yield were determined by GC using biphenyl as internal standard

Table S5: Evaluation of counter ion for different Pd(II) salts with TMEDA ligand

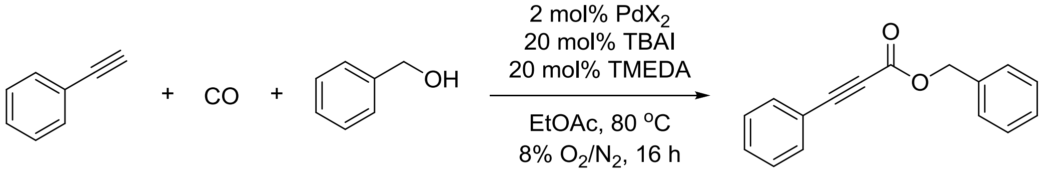

Entry	Catalyst	Conv. alkyne [%][a]	Conv. alcohol [%][a]	Yield of product [%][a]
1	PdI$_2$	6	0	0
2	PdCl$_2$	12	0	0
3	Pd(CF$_3$COO)$_2$	27	5	0
4[b]	Pd(OAc)$_2$	92	89	82
5[c]	Pd(OAc)$_2$	75	80	73
6	Pd((CH$_3$)$_3$COO)$_2$	90	87	84
7	Pd(C$_2$H$_5$COO)$_2$	80	89	74

Reaction conditions: 2 mol% PdX$_2$ (0.02 mmol), 20 mol% TBAI (0.20 mmol), 20 mol% TMEDA (0.20 mmol), phenylacetylene (1 mmol, 0.1020 g), benzyl alcohol (1 mmol, 0.1080 g), ethyl acetate (12 mL), CO : 8% O$_2$/N$_2$ = 5 bar : 35 bar, 80 °C, 16 hours. Experiment details see General Method B.
[a] Conversion and yield were determined by GC using biphenyl as internal standard.
[b] Pd(OAc)$_2$ with purity 99.9%, trace metal basis, purchased from Sigma Aldrich.
[c] Pd(OAc)$_2$ with purity 99%, purchased from Fluorochem.

Table S6: Solvent screening using $(Phen)Pd(OAc)_2$ as catalyst

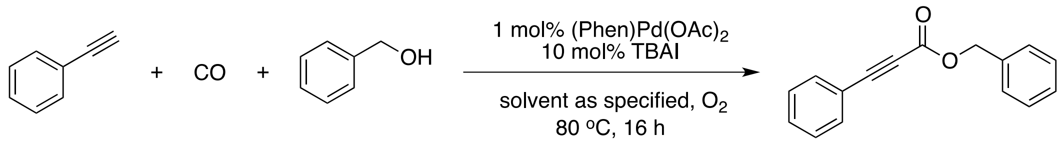

Entry	Solvent	Alkyn Conv. [%][a]	Alcohol Conv. [%][a]	Yield [%][a]
1	EtOAc	32	32	32
2	DMSO	55	52	1
3	Toluene	25	17	14
4	DMSO:Toluene = 0.4 mL : 3.6 mL	23	18	17
5	DMSO:Toluene = 3.6 mL : 0.4 mL	38	20	1
6	THF	28	33	23
7	DMF	26	13	13
8	1,4-dioxane	21	18	15
9	MeCN	17	11	9
10	Acetic acid	50	72	0
11	2-Butanone	24	23	18

Reaction conditions: 1 mol% $(Phen)Pd(OAc)_2$ (0.01 mmol, 0.0040 g), TBAI (10 mol%, 0.0374 g), phenylacetylene (1 mmol, 0.1020 g), benzyl alcohol (1 mmol, 0.1080 g), solvent as specified (4 mL), CO/O_2 = 1:1 (20 bar), 80 °C, 16 hours. Experimant details see General Method A.
[a] Conversion and yield were determined by GC using biphenyl as internal standard

Table S7: Temperature optimization using (Phen)Pd(OAc)$_2$ as catalyst

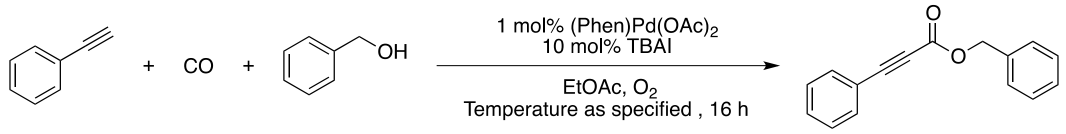

Entry	Temperature	Alkyn Conv. [%][a]	Alcohol Conv. [%][a]	Yield [%][a]
1	60 °C	22	24	22
2	80 °C	32	32	32
3	100 °C	32	42	32
4	120 °C	30	43	26

Reaction conditions: 1 mol% (Phen)Pd(OAc)$_2$ (0.01 mmol, 0.0040 g), TBAI (10 mol%, 0.0374 g), phenylacetylene (1 mmol, 0.1020 g), benzyl alcohol (1 mmol, 0.1080 g), ethyl acetate (4 mL), CO/O$_2$ = 1:1 (20 bar), 80 °C, 16 hours. Experimant details see General Method A.
[a] Conversion and yield were determined by GC using biphenyl as internal standard

Table S8: Influence of concentration using (Phen)Pd(OAc)$_2$ as catalyst

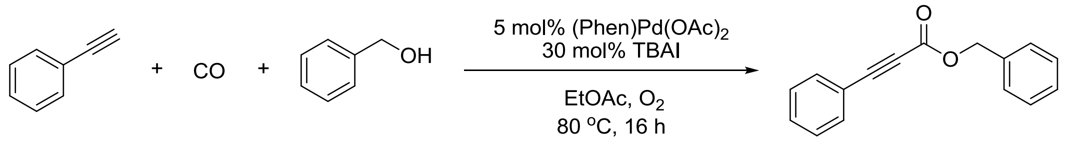

Entry	Ethyl acetate	Alkyn Conv. [%][a]	Alcohol Conv. [%][a]	Yield [%][a]
1	4 mL	85	75	50
2[b]	4 mL	82	42	70
3	8 mL	85	87	72
4[b]	8 mL	97	63	87
5	12 mL	88	92	80

Reaction conditions: 5 mol% (Phen)Pd(OAc)$_2$ (0.05 mmol, 0.0200 g), TBAI (30 mol%, 0.1124 g) , phenylacetylene (1 mmol, 0.1020 g), benzyl alcohol (1 mmol, 0.1080 g), amount of ethyl acetate as specified, CO/O$_2$ = 1:1 (20 bar), 80 °C, 16 hours. Experimant details see General Method B.
[a] Conversion and yield were determined by GC using biphenyl as internal standard
[b] 2 mmol alcohol

Table S9: Influence of selected ligands when a secondary aliphatic alcohol (2-octanol) is used as the nucelophile

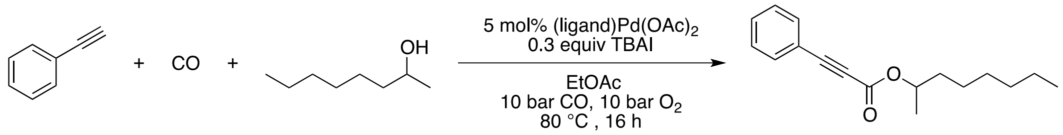

Entry	Ligand	Alcohol	Conv. alkyn [%][a]	Conv. alcohol [%][a]	Yield of product [%][a]
1	Bphen	1 equiv.	62	65	37
2	Bphen	2 equiv.	67	36	38
3	Phen	5 equiv.	67	20	36
4[b]	Phen	1 equiv.	56	51	36
5	DPPE[c]	1 equiv.	50	29	29
6	DPPE[c]	5 equiv.	82	18	65
7[b]	DPPE[c]	5 equiv.	100	25	95

Reaction conditions: 5 mol% pre-made & isolated (ligand)Pd(OAc)$_2$ (0.05 mmol), TBAI (30 mol%, 0.1024 g), phenylacetylene (1 mmol, 0.1020 g), 2-octanol (1 mmol, 0.1080 g), ethyl acetate (4 mL), CO/O$_2$ = 1:1 (20 bar), 80 °C, 16 hours. Experimant details see General Method B.
[a] Conversion and yield were determined by GC using biphenyl as internal standard
[b] 12 mL EtOAc was used as solvent instead of 4 mL EtOAc
[c] 5 mol% DPPE and 5 mol% Pd(OAc)$_2$ were added

Table S10: Testing secondary alcohols using Pd(OAc)$_2$ and DPPE

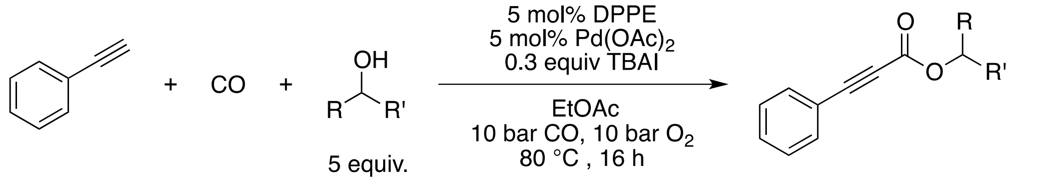

Entry	Alcohol	Convn. alkyn [%][a]	Yield [%][a]
1	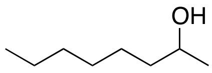	100	95
2	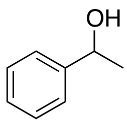	99	74
3	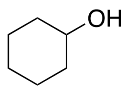	97	77

Reaction conditions: 5 mol% DPPE (0.005 mmol, 0.0199 g), 5 mol% Pd(OAc)$_2$ (0.0112 g, 0.05 mmol), TBAI (30 mol%, 0.1024 g), phenylacetylene (1 mmol, 0.1020 g), alcohol (5 mmol), ethyl acetate (12 mL), CO/O$_2$ = 1:1 (20 bar), 80 °C, 16 hours. Experimant details see General Method B.
[a] Conversion and yield were determined by GC using biphenyl as internal standard

Table S11: Study of CO and O_2 gas mixtures with Phen and DPPE ligands

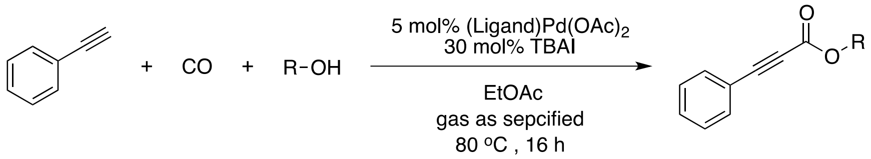

Entry	Ligand	Alcohol	Gas mixture	Alkyn Conv. [%][a]	Yield [%][a]
1	Phen	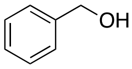	CO : O_2 = 10 bar : 10 bar	88	80
2	Phen	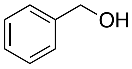	CO : 8% O_2 in N_2 = 5 bar : 35 bar	70	52
3	Phen	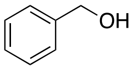	CO : 8% O_2 in CO_2 = 5 bar : 35 bar	65	48
4[b]	DPPE	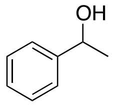	CO : O_2 = 10 bar : 10 bar	99	74
5[b]	DPPE	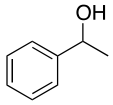	CO : Air = 5 bar : 35 bar	99	66
6[b]	DPPE	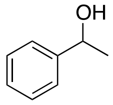	CO : 8% O_2 in N_2 = 5 bar : 35 bar	69	36

Reaction conditions: Premade & isolated 5 mol% (Phen)Pd$(OAc)_2$, TBAI (30 mol%, 0.1024 g), phenylacetylene (1 mmol, 0.1020 g), primary alcohol (1 mmol) or secondary alcohol (5 mmol), ethyl acetate (12 mL), gas as specified, 80 °C, 16 hours. Experimant details see General Method B and C.
[a] Conversion and yield were determined by GC using biphenyl as internal standard
[b] 5 mol% DPPE (0.005 mmol, 0.0199 g), 5 mol% Pd$(OAc)_2$ (0.0112 g, 0.05 mmol) were used as catalyst

Table S12: Optimization of conditions using TMEDA as a ligand with a primary alcohol

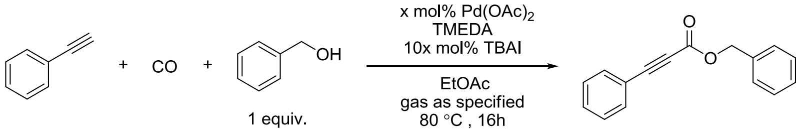

Entry	TMEDA	$Pd(OAc)_2$	Gas mixture	EtOAc	Conv. alkyn [%][a]	Conv. alcohol [%][a]	Yield [%][a]
1	10 mol%	1 mol%	10 bar CO : 10 bar O_2	4 mL	86	85	75
2	10 mol%	1 mol%	5 bar CO : 35 bar Air	4 mL	60	43	35
3	20 mol%	1 mol%	5 bar CO : 35 bar Air	4 mL	65	47	35
4	30 mol%	3 mol%	5 bar CO : 35 bar Air	4 mL	85	62	30
5	30 mol%	3 mol%	5 bar CO : 35 bar Air	12 mL	95	96	83
6	20 mol%	2 mol%	5 bar CO : 35 bar Air	12 mL	95	96	84
7	10 mol%	1 mol%	5 bar CO : 35 bar Air	12 mL	96	90	85
8	10 mol%	1 mol%	5 bar CO : 35 bar 8% O_2 in N_2	12 mL	47	45	42
**9**	**20 mol%**	**2 mol%**	**5 bar CO : 35 bar 8 %O_2 in N_2**	**12 mL**	**92**	**89**	**82**
10	40 mol%	2 mol%	5 bar CO : 35 bar 8 %O_2 in N_2	12 mL	91	95	82

Reaction conditions: $Pd(OAc)_2$ (amount as specified), TMEDA (amount as specified), TBAI (amount as specified), phenylacetylene (1 mmol, 0.1020 g), benzyl alcohol (1 mmol, 0.1080 g), ethyl acetate (12 mL), gas as specified, 80 °C, 16 hours. Experimant details see General Method B.
[a] Conversion and yield were determined by GC using biphenyl as internal standard

Table S13: Optimization of conditions using TMEDA as a ligand with a secondary alcohol

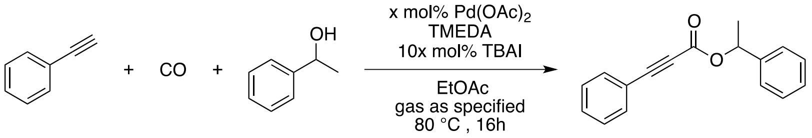

Entry	TMEDA	$Pd(OAc)_2$	Alcohol amount	Gas mixture	Conv. alkyn [%][a]	Yield [%][a]
1	10 mol% TMEDA	1 mol%	5 equiv.	10 bar CO : 10 bar O_2	47	43
2	30 mol% TMEDA	3 mol%	5 equiv.	10 bar CO : 10 bar O_2	88	77
3	30 mol% TMEDA	3 mol%	5 equiv.	5 bar CO : 35 bar Air	93	76
4	30 mol% TMEDA	3 mol%	2 equiv.	5 bar CO : 35 bar Air	95	80
5	30 mol% TMEDA	3 mol%	1.5 equiv.	5 bar CO : 35 bar Air	95	76
6	30 mol% TMEDA	3 mol%	1 equiv.	5 bar CO : 35 bar Air	81	58
7	30 mol% TMEDA	3 mol%	5 equiv.	5 bar CO : 35 bar 8 %O_2 in N_2	86	76
**8**	**30 mol% TMEDA**	**3 mol%**	**2 equiv.**	**5 bar CO : 35 bar 8 %O_2 in N_2**	**92**	**75**
9	30 mol% TMEDA	3 mol%	1.5 equiv.	5 bar CO : 35 bar 8 %O_2 in N_2	78	52
10[b]	30 mol% TMEDA	3 mol%	1 equiv.	5 bar CO : 35 bar Air	43	44[c]

Reaction conditions: $Pd(OAc)_2$ (3 mol%, 0.03 mmol, 0.0067 g), TMEDA (30 mol%, 0.3 mmol, 0.0348 g), TBAI (30 mol%, 0.3 mmol, 0.1124), phenylacetylene (1 mmol, 0.1020 g), 1-phenylethanol (5 mmol, 0.6536 g), ethyl acetate (12 mL), gas as specified, 80 °C, 16 hours. Experimant details see General Method C.
[a] Conversion and yield were determined by GC using biphenyl as internal standard
[b] 2 mmol alkyn with 1 mmol alcohol
[c] Yield was calculated with regard to 1-phenylethanol

4 Product Characterization data

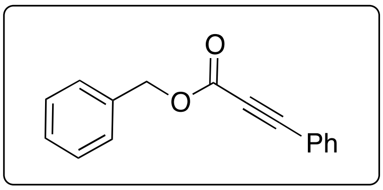

Benzyl 3-phenylpropiolate (1)

Purified by column chromatography (diethyl ether/Petroleum ether = 5: 100) to afford **1** as a colorless oil (0.1960 g, 83%). ^{1}H NMR (400 MHz, $CDCl_3$): δ 7.60-7.56 (m, 2H), 7.48-7.34 (m, 8H), 5.27 (s, 2H); ^{13}C NMR (100 MHz, $CDCl_3$): δ 154.0, 135.1, 133.2, 130.8, 128.8, 128.7, 119.7, 86.9, 80.6, 67.9. NMR data is consistent with literature values.[1] HRMS (ESI^+) Calc. for $C_{16}H_{13}O_2$ [$M+H^+$] 237.0916, found: 237.0808. IR (neat): 2220, 1705, 1281, 1165 cm^{-1}. IR data is consistent with literature values.[1, 2]

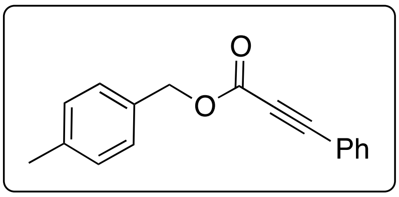

4-Methylbenzyl 3-phenylpropiolate (2)

Purified by flash column chromatography (diethyl ether/petroleum ether = 5: 100) to afford **2** as a golden yellow oil (0.1975 g, 78 %). ^{1}H NMR (400 MHz, $CDCl_3$): δ 7.58-7.54 (m, 2H), 7.46-7.40 (m, 1H), 7.39-7.29 (m, 4H), 7.22-7.16 (m, 2H), 5.22 (s, 2H), 2.36 (s, 3H); ^{13}C NMR (100 MHz, $CDCl_3$): 154.1, 138.7, 133.2, 132.1, 130.8 129.5, 129.0, 128.7, 119.8, 86.7, 80.8, 67.9, 21.4. HRMS (ESI^+) Calc. for $C_{17}H_{15}O_2$ [$M+H^+$] 251.1072, found: 251.1065. IR (neat): 2218, 1704, 1280, 1285, 1164, 756 cm^{-1}.

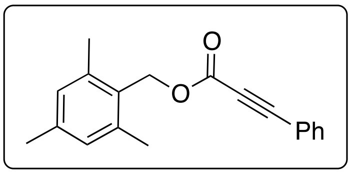

2,4,6-Trimethylbenzyl 3-phenylpropiolate (3)

Purified by flash column chromatography (diethyl ether/petroleum ether = 5: 100) to afford **3** as a bright yellow crystalline solid (0.1422 g, 49 %). m.p. 63-65 °C. ^{1}H NMR (400 MHz, $CDCl_3$): δ 7.57-7.53 (m, 2H), 7.45-7.40 (m, 1H), 7.38-7.32 (m, 2H), 6.91-6.88 (m, 2H), 5.32 (s, 2H), 2.39 (s, 6H), 2.28 (s, 3H). ^{13}C NMR (100 MHz, $CDCl_3$): δ 154.5, 139.1, 138.6, 133.2, 130.8, 129.4, 128.7, 128.3, 119.8, 86.6, 80.7, 62.7, 21.2, 19.7. HRMS (ESI$^+$) Calc. for $C_{19}H_{19}O_2$ [M+H$^+$] 279.1385, found: 279.1375. IR (neat): 2221, 1703, 1277, 1167, 748, 686 cm^{-1}.

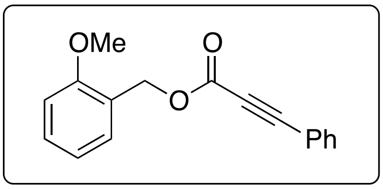

2-Methoxybenzyl 3-phenylpropiolate (4)

Purified by column chromatography (diethyl ether/Petroleum ether = 5: 100) to afford **4** as a colorless oil (0.1889 g, 71%). ^{1}H NMR (400 MHz, $CDCl_3$): δ 7.60-7.55 (m, 2H), 7.47-7.30 (m, 5H), 6.98 (td, *J* = 7.5, 1.0, 1H), 6.91 (d, *J* = 8.3 Hz, 1H), 5.33 (s, 2H), 3.87 (s, 3H); ^{13}C NMR (100 MHz, $CDCl_3$): δ 157.9, 154.2, 133.1, 130.7, 130.4, 130.2, 128.7, 123.4, 120.6, 119.85, 110.7, 86.5, 80.7, 63.4, 55.6. HRMS (ESI$^+$) Calc. for $C_{17}H_{14}O_3Na$ [M+Na$^+$]: 289.0841, found: 289.0851. IR (neat): 2220, 1705, 1281, 1249, 1165, 1028 cm^{-1}.

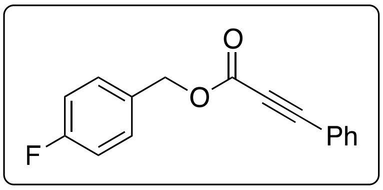

4-Fluorobenzyl 3-phenylpropiolate (5)

Purified by column chromatography (EtOAc/Petroleum ether = 5: 100) to afford **5** as colorless oil (0.2031 g, 80%). ^{1}H NMR (400 MHz, $CDCl_3$): δ 7.61-7.55 (m, 2H), 7.48-7.34 (m, 5H), 7.11-7.04 (m, 2H), 5.23 (s, 2H); ^{13}C NMR (100 MHz, $CDCl_3$): δ 164.1, 161.7, 153.8, 133.0, 130.8, 130.6, 128.6, 119.5, 115.7, 115.5, 86.9, 80.4, 66.9. NMR data is consistent with literature values.[3] HRMS (ESI$^+$) Calc. for $C_{16}H_{12}FO_2$ [M+H$^+$]: 255.0821, found: 255.0816. IR (neat): 2220, 1705, 1512, 1282, 1165, 1155 cm^{-1}. IR data is consistent with literature values.[3]

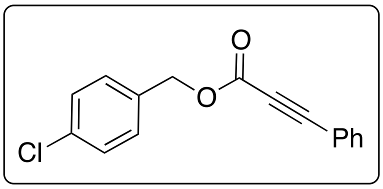

4-Chlorobenzyl 3-phenylpropiolate (6)

Purified by column chromatography (EtOAc/Petroleum ether = 5: 100) to afford **6** as a white crystalline solid (0.2325 g, 86%). m.p. 59-61 °C. ^{1}H NMR (400 MHz, $CDCl_3$): δ 7.61-7.55 (m, 2H), 7.48-7.42 (m, 1H), 7.40-7.32 (m, 6H), 5.22 (s, 2H); ^{13}C NMR (100 MHz, $CDCl_3$): δ 153.9, 134.7, 133.6, 133.2, 130.9, 130.1, 129.0, 128.7, 119.6, 87.2, 80.5, 67.0. HRMS (ESI$^+$) Calc. for $C_{16}H_{12}ClO_2$ [M+H$^+$] 271.0526, found: 271.0494. IR (neat): 2215, 1700, 1489, 1285, 1166, 757 cm^{-1}.

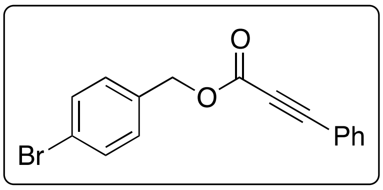

4-Bromobenzyl 3-phenylpropiolate (7)

Purified by column chromatography (EtOAc/Petroleum ether = 5: 100) to afford **7** as a light yellow crystalline solid (0.2512 g, 80%). m.p. 62-63°C. ^{1}H NMR (400 MHz, $CDCl_3$): δ 7.58-7.28 (m, 9H), 5.20 (s, 2H); ^{13}C NMR (100 MHz, $CDCl_3$): δ 153.9, 134.1, 133.2, 132.0, 130.9, 130.4, 128.7, 122.9, 119.6, 87.2, 80.4, 67.0. NMR data is consistent with literature values.[4] HRMS (ESI$^+$) Calc. for $C_{16}H_{12}O_2Br$ [M+H$^+$]: 315.0021, found 314.9982. IR (neat): 2217, 1700, 1486, 1285, 1169, 758, 688 cm^{-1}. IR data is consistent with literature values.[4]

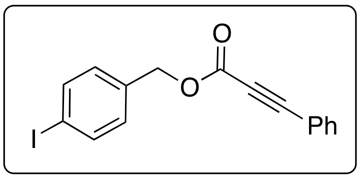

4-Iodobenzyl 3-phenylpropiolate (8)

Purified by flash column chromatography (ethyl acetate /petroleum ether = 5: 100) to afford **8** as a pale yellow oil (0.1068 g, 29 %). ^{1}H NMR (400 MHz, $CDCl_3$): δ 7.77-7.71 (m, 2H), 7.62-7.58 (m, 2H), 7.49-7.44 (m, 1H), 7.42-7.36 (t, *J* = 7.5 Hz, 2H), 7.21-7.16 (d, *J* = 8.3 Hz, 2H), 5.21 (s, 2H). ^{13}C NMR (100 MHz, $CDCl_3$): δ 153.9, 138.0, 134.8, 133.2, 130.6, 128.8, 119.6, 94.6, 87.3, 80.5, 67.1. HRMS (ESI$^+$) Calc. for $C_{16}H_{15}INO_2$ [M+NH_4^+] 380.0148 found: 380.0163. IR (neat): 2210, 1704, 1283, 1189, 1170, 760 cm^{-1}.

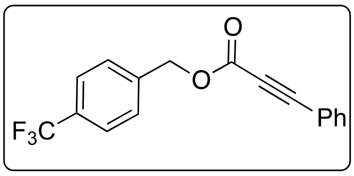

4-(Trifluoromethyl)benzyl 3-phenylpropiolate (9)

Purified by flash column chromatography (diethyl ether/petroleum ether = 5: 100) to afford **9** as a pale yellow crystalline solid (0.2404 g, 81 %). m.p. 50-51 °C. ^{1}H NMR (400 MHz, $CDCl_3$): δ 7.68-7.62 (d, *J* = 8.1 Hz, 2H), 7.62-7.57 (m, 2H), 7.56-7.52 (d, *J* = 8.1 Hz, 2H), 7.49-7.43 (m, 1H), 7.41-7.35 (m, 2H), 5.31 (s, 2H). ^{13}C NMR (100 MHz, $CDCl_3$): δ 153.8, 139.1, 133.3, 131.1, 128.7, 125.8, 119.5, 87.5, 80.4, 66.8. NMR Data is in correlation with literature.[5] HRMS (ESI$^+$) Calc. for $C_{17}H_{12}F_3O_2$ [M+H$^+$] 305.0789, found: 305.0765. IR (neat): 2218, 1706, 1285, 1191, 1067, 760 cm^{-1}.

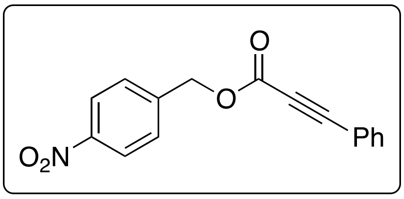

4-Nitrobenzyl 3-phenylpropiolate (10)

Purified by column chromatography (EtOAc/Petroleum ether = 10: 100) to afford **10** as a light yellow crystalline solid (0.2136 g, 76%). m.p. 81-82 °C ^{1}H NMR (400 MHz, $CDCl_3$): δ 8.28-8.23 (m, 2H), 7.63-7.56 (m, 4H), 7.51-7.44 (m, 1H), 7.42-7.35 (m, 2H), 5.35 (s, 2H); ^{13}C NMR (100 MHz, $CDCl_3$): δ 153.6, 148.1, 142.3, 132.3, 133.3, 131.1, 128.8, 124.1, 119.4, 87.9, 80.1, 66.1. NMR data is consistent with literature values.[3] HRMS (ESI$^+$) Calc. for $C_{16}H_{15}N_2O_4$ [M+NH_4^+] 299.1031 Found: 299.1063. IR (neat): 2217, 1693, 1520, 1350, 1292 cm^{-1}. IR data is consistent with literature values.[3]

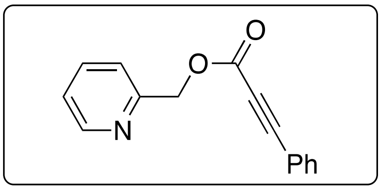

Pyridine-2-ylmethyl 3-phenylpropiolate (11)

Purified by column chromatography (EtOAc/Petroleum ether = 20: 100) to afford **11** as a yellow oil (0.0925 g, 39%). ^{1}H NMR (400 MHz, $CDCl_3$): δ 8.63 (ddd, *J* = 4.9, 1.7, 0.9 Hz, 1H), 7.74 (td, *J* = 7.7, 1.8 Hz, 1H), 7.62-7.57 (m, 2H), 7.49-7.43 (m, 4H), 7.26 (m, 1H), 5.34 (s, 2H); ^{13}C NMR (100 MHz, $CDCl_3$): δ 155.0, 153.8, 149.8, 137.1, 133.2, 130.9, 128.7, 123.3, 122.1, 119.6, 87.4, 80.5, 68.2. HRMS (ESI^+) Calc. for $C_{15}H_{12}NO_2$ [$M+H^+$]: 238.0868, found: 238.0844. IR (neat): 2224, 1710, 1283, 1167, 1000 cm^{-1}.

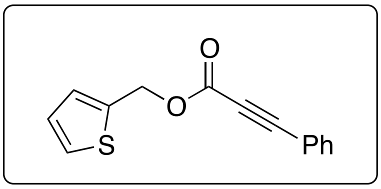

Thiophen-2-ylmethyl 3-phenylpropiolate (12)

Purified by column chromatography (EtOAc/Petroleum ether = 5: 100) to afford **12** as light yellow crystalline solid (0.2057 g, 85%). m.p. 46-48 °C. ^{1}H NMR (400 MHz, $CDCl_3$): δ 7.59-7.55 (m, 2H), 7.47-7.42 (m, 1H), 7.40-7.34 (m, 3H), 7.20-7.16 (m, 1H), 7.02 (dd, *J* = 5.1, 3.5 Hz, 1H), 5.42 (s, 2H); ^{13}C NMR (100 MHz, $CDCl_3$): δ 153.8, 136.8, 133.2, 130.9, 129.3, 128.7 127.6, 127.1, 119.6, 87.1, 80.5, 61.8. HRMS (ESI^+) Calc. for $C_{14}H_{11}O_2S$ [$M+H^+$] 243.0480, found: 243.0474. IR (neat): 2217, 1695, 1489, 1436, 1287, 1168 cm^{-1}.

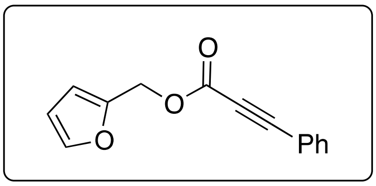

(Tetrahydrofuran-2-yl)methyl 3-phenylpropiolate (13)

Purified by flash column chromatography (ethyl acetate /petroleum ether = 5: 100) to afford **13** as a golden yellow oil (0.1692 g, 76 %). ^{1}H NMR (400 MHz, $CDCl_3$): δ 7.59-7.53 (m, 2H), 7.47-7.40 (m, 2H), 7.39-7.32 (m, 2H), 6.50-6.47 (d, *J* = 3.3 Hz, 1H), 6.40-6.36 (dd, *J* =1.9, 3.2 Hz, 1H), 5.21 (s, 2H); ^{13}C NMR (100 MHz, $CDCl_3$): δ 153.8, 148.61, 143.7, 133.2, 130.8, 128.7, 119.6, 111.5, 110.9, 87.2, 80.4, 59.4.HRMS (ESI$^+$) Calc. for $C_{14}H_{11}O_3$ [M+H$^+$] 227.0708, found: 227.0705. IR (neat): 2220, 1706, 1280, 1162, 743 cm^{-1}.

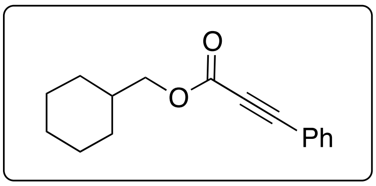

Cyclohexylmethyl 3-phenylpropiolate (14)

Purified by flash column chromatography (diethyl ether/petroleum ether = 5: 100) to afford **14** as a pale yellow oil (0.1845 g, 73 %). ^{1}H NMR (400 MHz, $CDCl_3$): δ 7.52-7.48(m, 2H), 7.38-7.33 (m, 1H), 7.31-7.25 (m, 2H), 3.99-3.93 (d, *J* = 6.6 Hz, 2H), 1.75-1.55 (m, 6H), 1.22-1.07 (m, 3H), 0.98-0.86 (m, 2H); ^{13}C NMR (100 MHz, $CDCl_3$): δ 154.4, 133.1, 130.7, 128.7, 119.8, 86.2, 80.9, 71.2, 37.1, 29.6, 26.3, 25.7. HRMS (ESI$^+$) Calc. for $C_{16}H_{19}O_2$ [M+H$^+$] 243.1385, found: 243.1388. IR (neat): 2927, 2226, 1705, 1280, 1170, 756 cm^{-1}.

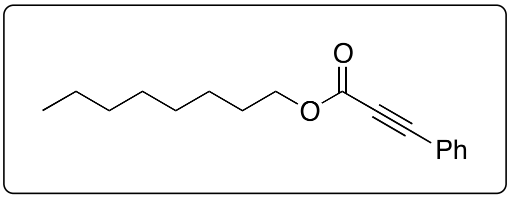

Octyl 3-phenylpropiolate (15)

Purified by column chromatography (diethyl ether/Petroleum ether = 5: 100) to afford **15** as a colorless oil (0.1807 g, 70%). ^{1}H NMR (400 MHz, $CDCl_3$): δ 7.61-7.57 (m, 2H), 7.47-7.42 (m, 1H), 7.40-7.34 (m, 2H), 4.23 (t, *J* = 6.8 Hz, 2H), 1.71 (quint, *J* = 6.8 Hz, 2H), 1.44-1.23 (m, 10H), 0.89 (t, *J* = 6.8 Hz, 3H); ^{13}C NMR (100 MHz, $CDCl_3$): δ 154.4, 133.1, 130.7, 128.7, 119.9, 86.2, 80.9, 66.4, 31.9, 29.3, 28.6, 26.0, 22.8, 14.2. NMR data is consistent with literature values.[6] HRMS (ESI$^+$) Calc. for $C_{17}H_{23}O_2$ [M+H$^+$] 259.1698, found: 259.1706. IR (neat): 2225, 1708, 1283, 1171 cm^{-1}. IR data is consistent with literature values.[6]

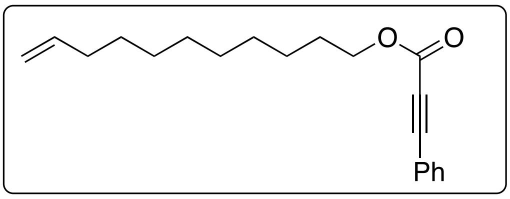

Undec-10-en-1-yl 3-phenylpropiolate (16)

Purified by column chromatography (diethyl ether/Petroleum ether = 5: 100) to afford **16** as a colorless oil (0.2087 g, 70%). ^{1}H NMR (400 MHz, $CDCl_3$): δ 7.61-7.57 (m, 2H), 7.47-7.42 (m, 1H), 7.40-7.34 (m, 2H), 5.81 (ddt, *J* = 16.9, 10.2, 6.7 Hz, 1H), 5.03-4.90 (m, 2H), 4.23 (t, *J* = 6.8 Hz, 2H), 2.08-2.00 (m, 2H), 1.71 (quint, *J* = 6.8 Hz, 2H), 1.45-1.25 (m, 12H); ^{13}C NMR (100 MHz, $CDCl_3$): δ 154.4, 139.3, 133.1, 130.7, 128.7, 119.9, 114.3, 86.2, 80.9, 66.4, 33.9, 29.6, 29.5, 29.3, 29.2, 29.1, 28.6, 26.0. HRMS (ESI$^+$) Calc. for $C_{20}H_{27}O_2$ [M+H$^+$] 299.2011, found: 299.1972. IR (neat): 2226, 1708, 1642 (C=C), 1283, 1172 cm^{-1}.

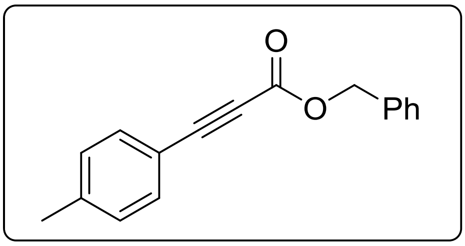

Benzyl 3-(*p*-tolyl)propiolate (17)

Purified by flash column chromatography (diethyl ether/petroleum ether = 5: 100) to afford **17** as a dark yellow solid (0.1951 g, 81 %). m.p. 72-75 °C. ^{1}H NMR (400 MHz, $CDCl_3$): δ 7.49-7.45(d, *J* = 8.0, 2H), 7.44-7.33 (m, 5H), 7.20-7.14 (d, *J* = 7.9, 2H), 5.26 (s, 2H), 2.37 (s, 3H). ^{13}C NMR (100 MHz, $CDCl_3$): δ 154.2, 141.6, 135.2, 133.2, 129.6, 128.8, 116.6, 87.5, 80.4, 67.8, 21.9. NMR data is consistent with literature values.[5] HRMS (ESI$^+$) Calc. for $C_{17}H_{15}O_2$ [M+H$^+$] 251.1072, found: 251.1082. IR (neat): 2215, 1695, 1288, 1166, 756 cm^{-1}.

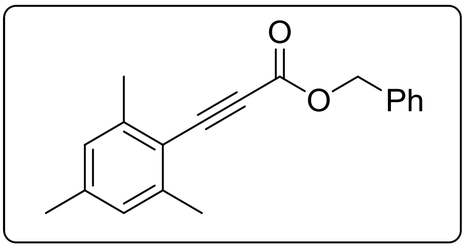

Benzyl 3-mesitylpropiolate (18)

Purified by flash column chromatography (diethyl ether/petroleum ether = 5: 100) to afford **18** as a golden orange oil (0.2552 g, 91 %). ^{1}H NMR (400 MHz, $CDCl_3$): δ 7.44-7.32(m, 5H), 6.90-6.84 (s, 2H), 5.27 (s, 2H), 2.45-2.41(d, *J* = 4.5 Hz, 6H), 2.30-2.26 (d, *J* = 9.3 Hz, 3H). ^{13}C NMR (100 MHz, $CDCl_3$): δ 154.5, 142.7, 140.8, 135.5, 128.8, 128.6, 128.1, 116.6, 88.2, 85.5, 67.6, 21.7, 21.0. HRMS (ESI$^+$) Calc. for $C_{19}H_{19}O_2$ [M+H$^+$] 279.1385, found: 279.1387. IR (neat): 2212, 1701, 1265,1206, 1147, 752 cm^{-1}.

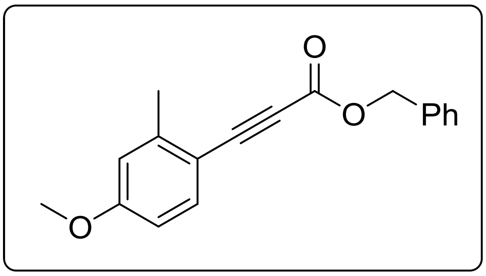

Benzyl 3-(4-methoxy-2-methylphenyl)propiolate (19)

Purified by flash column chromatography (ethyl acetate/petroleum ether = 10: 100) to afford **19** as an off white solid (0.2150 g, 77 %). m.p. 38-39 °C. ^{1}H NMR (400 MHz, $CDCl_3$): δ 7.50-7.46 (d, *J* = 8.5, 1H), 7.44-7.32 (m, 5H), 6.76-6.74 (m, 1H), 6.73-6.68 (m, 1H), 5.26 (s, 2H), 3.81 (s, 3H), 2.46 (s, 3H). ^{13}C NMR (100 MHz, $CDCl_3$): δ 161.7, 154.5, 144.8, 135.5, 135.4, 128.8, 128.7, 115.5, 111.8, 86.9, 83.8, 67.6, 55.5, 21.0 HRMS (ESI^+) Calc. for $C_{18}H_{17}O_3$ [$M+H^+$] 281.1178, found: 281.1175. IR (neat): 2205, 1693, 1287,1239, 1160, 741 cm^{-1}.

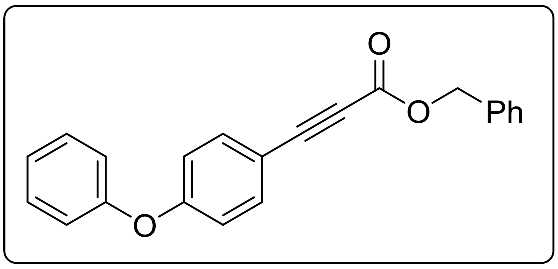

Benzyl 3-(4-phenoxyphenyl)propiolate (20)

Purified by flash column chromatography (ethyl acetate/petroleum ether = 4: 100) to afford **20** as a yellow oil (0.1604 g, 49 %). ^{1}H NMR (400 MHz, $CDCl_3$): δ 7.55-7.50 (m, 2H), 7.44-7.34 (m, 7H), 7.21-7.15 (m, 1H), 7.08-7.01 (m, 2H), 6.96-6.90 (m, 2H), 5.26 (s, 2H). ^{13}C NMR (100 MHz, $CDCl_3$): δ 160.2, 155.7, 154.2, 135.2, 130.2, 128.8, 126.8, 124.8, 120.3, 118.1, 113.6, 87.1, 80.4, 67.8. HRMS (ESI^+) Calc. for $C_{22}H_{17}O_3$ [$M+H^+$] 329.1178, found: 329.1186. IR (neat): 2218, 1704, 1502, 1237, 1177, 692 cm^{-1}.

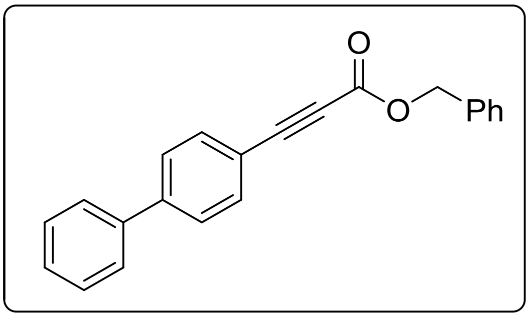

Benzyl 3-([1,1'-biphenyl]-4-yl)propiolate (21)

Purified by flash column chromatography (diethyl ether/petroleum ether = 5: 100) to afford **21** as a yellow crystalline solid (0.1595 g, 51 %). m.p. 101-102 °C. ^{1}H NMR (400 MHz, $CDCl_3$): δ 7.67-7.63 (m, 2H), 7.62-7.57 (m, 4H), 7.48-7.36 (m, 8H), 5.28 (s, 2H). ^{13}C NMR (100 MHz, $CDCl_3$): δ 154.1, 143.7, 140.0, 135.1, 133.7, 129.2, 128.8, 128.3, 127.4, 118.5, 87.0, 81.3, 67.9. HRMS (ESI^+) Calc. for $C_{22}H_{17}O_2$ [$M+H^+$], 313.1229 found:, 313.1221. IR (neat): 2208, 1701, 1288,838, 755 cm^{-1}.

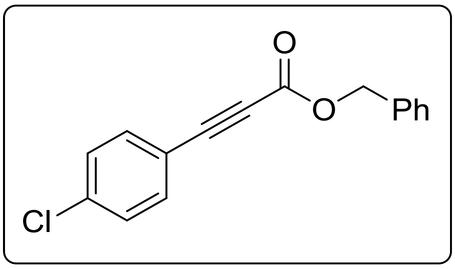

Benzyl 3-(4-chlorophenyl)propiolate (22)

Purified by flash column chromatography (ethyl acetate/petroleum ether = 5: 100) to afford **22** as a dark orange crystalline solid (0.1675 g, 62 %). m.p. 65-67 °C. ^{1}H NMR (400 MHz, $CDCl_3$): δ 7.52-7.48 (m, 2H), 7.42-7.35 (m, 6H), 7.34-7.33 (m, 1H), 5.26 (s, 2H); ^{13}C NMR (100 MHz, $CDCl_3$): δ 153.9, 137.3, 135.0, 134.4, 129.2, 128.9, 118.2, 85.5, 81.5, 68.0. HRMS (ESI^+) Calc. for $C_{16}H_{12}ClO_2$ [$M+H^+$] 271.0526, found: 271.0530. IR (neat): 2215, 1695, 1287, 1179,1163, 1086, 823 cm^{-1}.

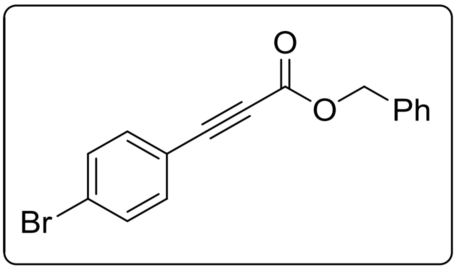

Benzyl 3-(4-bromophenyl)propiolate (23)

Purified by flash column chromatography (ethyl acetate/petroleum ether = 5: 100) to afford **23** as a yellow crystalline solid (0.1650 g, 53 %). m.p. 87-89 °C. ^{1}H NMR (400 MHz, $CDCl_3$): δ 7.55-7.49 (m, 2H), 7.46-7.33 (m, 7H), 5.26 (s, 2H). ^{13}C NMR (100 MHz, $CDCl_3$): δ 153.9, 134.5, 132.2, 128.8, 125.7, 118.7, 85.6, 81.6, 68.0. HRMS (ESI^{+}) Calc. for $C_{16}H_{12}O_2Br$ [M+H^{+}] 315.0021, found: 315.0010. IR (neat): 2224, 1695, 1288, 1180,1163, 950, 742 cm^{-1}.

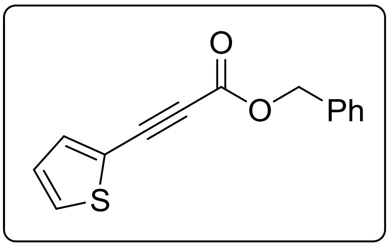

Benzyl 3-(thiophen-2-yl)propiolate (24)

Purified by flash column chromatography (ethyl acetate/petroleum ether = 5: 100) to afford **24** as a yellow crystalline solid (0.1050 g, 44 %). m.p. 70-72 °C. ^{1}H NMR (400 MHz, $CDCl_3$): δ 7.76-7.73 (dd, J = 3.0, 1.2 Hz, 1H), 7.44-7.35 (m, 5H), 7.32-7.29 (m, 1H), 7.23-7.21 (dd, J = 5.0, 1.2 Hz, 1H), 5.25 (s, 2H); ^{13}C NMR (100 MHz, $CDCl_3$): δ 154.1, 135.1, 134.1, 128.8, 126.3, 119.0, 82.4, 80.7, 67.9. HRMS (ESI^{+}) Calc. for $C_{14}H_{11}O_2S$ [M+H^{+}] 243.0480, found: 243.0518. IR (neat): 2215, 1700, 1260, 1206,1152, 753 cm^{-1}.

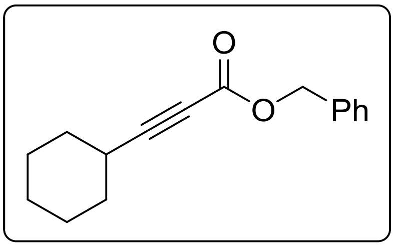

Benzyl 3-cyclohexylpropiolate (25)

Purified by flash column chromatography (diethyl ether/petroleum ether = 5: 100) to afford **25** as a pale yellow oil (0.1981 g, 81 %). ^{1}H NMR (400 MHz, $CDCl_3$): δ 7.41-7.29 (m, 5H), 5.17 (s, 2H), 2.54-2.44 (m, 1H), 1.87-1.76 (m, 2H), 1.75-1.65 (m, 2H), 1.57-1.42 (m, 3H), 1.38-1.22 (m, 3H). ^{13}C NMR (100 MHz, $CDCl_3$): δ 154.0, 135.3, 128.7, 93.7, 73.0, 69.9, 67.5, 31.6, 29.0, 25.7, 24.8. NMR data is consistent with literature values.[7] HRMS (ESI$^+$) Calc. for $C_{16}H_{19}O_2$ [M+H$^+$] 243.1385, found: 243.1390. IR (neat): 2931, 2229, 1706, 1450, 1233, 1085, 989, 749 cm^{-1}.

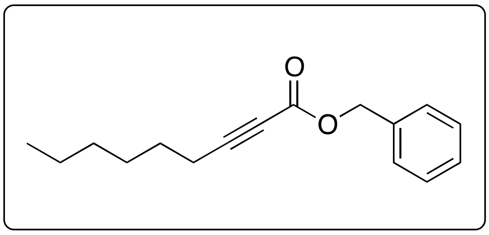

Benzyl non-2-ynoate (26)

Purified by column chromatography (diethyl ether/Petroleum ether = 5: 100) to afford **26** as a colorless oil (0.1368 g, 56%). ^{1}H NMR (400 MHz, $CDCl_3$): δ 7.37-7.26 (m, 5H), 5.18(s, 2H), 2.34-2.29 (m, 2H), 1.61-1.52 (m, 2H), 1.43-1.34 (m, 2H), 1.29-1.28 (m, 4H), 0.90-0.86 (m, 3H); ^{13}C NMR (100 MHz, $CDCl_3$): δ 153.9, 135.2, 128.8, 128.7, 128.6, 90.4, 73.1, 67.5, 31.3, 28.7, 27.6, 22.6, 18.9, 14.1. NMR data is consistent with literature values.[7] HRMS (ESI$^+$) Calc. for $C_{16}H_{21}O_2$ [M+H$^+$] 245.1542, found: 245.1540. IR (neat): 2235, 1709, 1237, 1066, 1120 cm^{-1}.

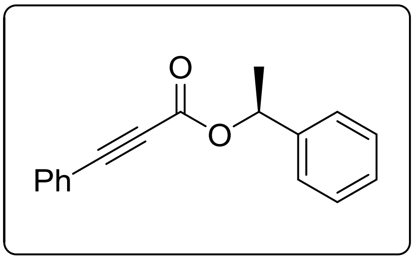

(*S*)-1-Phenylethyl 3-phenylpropiolate (27)*

Purified by flash column chromatography (ethyl acetate/petroleum ether = 5: 100) to afford **27** as a golden yellow oil (0.1916 g, 72 %). ^{1}H NMR (400 MHz, $CDCl_3$): δ 7.59-7.54 (m, 2H), 7.45-7.28 (m, 8H), 6.05-5.98 (q, J = 6.6 Hz, 1H), 1.65-1.61 (d, J = 6.6 Hz, 3H). ^{13}C NMR (100 MHz, $CDCl_3$): δ 153.5, 140.8, 133.1, 130.7, 128.4, 126.7, 126.4, 119.8, 86.4, 81.0, 74.5, 22.2.HRMS (ESI$^+$) Calc. for $C_{17}H_{15}O_2$ [M+H$^+$] 251.1072, found: 251.1066. IR (neat): 2215, 1702, 1279, 1185, 1170, 1057, 756 cm^{-1}.

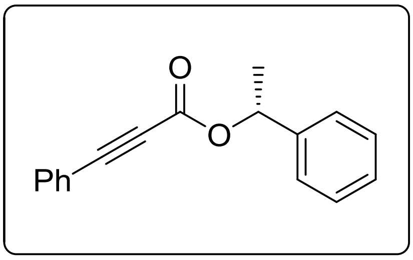

(*R*)-1-Phenylethyl 3-phenylpropiolate (28)*

Purified by column chromatography (ethyl acetate/petroleum ether = 5: 100) to afford **28** as a golden yellow oil (0.1834 g, 73 %). ^{1}H NMR (400 MHz, $CDCl_3$): δ 7.60-7.53 (m, 2H), 7.46-7.26 (m, 8H), 6.06-5.98 (q, J = 6.6 Hz, 1H), 1.66-1.59(d, J = 6.6 Hz, 3H); ^{13}C NMR (100 MHz, $CDCl_3$): δ 153.5, 140.8, 133.1, 130.8, 128.8, 128.3, 126.4, 119.8, 86.4, 81.0, 74.5, 22.2. HRMS (ESI$^+$) Calc. for $C_{17}H_{15}O_2$ [M+H$^+$] 251.1072, found: 251.1076. IR (neat): 2218, 1703, 1279, 1185, 1170, 1057, 756 cm^{-1}.

* **27** and **28** were prepared using (*S*)-(−)-1-Phenylethanol and (*R*)-(+)-1-phenylethanol respectively. Chiral HPLC was used to analyze chirality of the products.

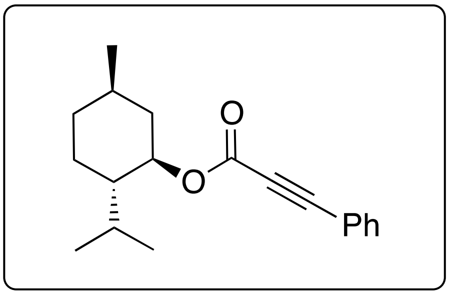

(1*R*,2*S*,5*R*)-2-isopropyl-5-methylcyclohexyl 3-phenylpropiolate (29)

Purified by column chromatography (diethyl ether/Petroleum ether = 5: 100) to afford **29** as light yellow oil (0.2472 g, 87%). ^{1}H NMR (400 MHz, $CDCl_3$): δ 7.63-7.55 (m, 2H), 7.48-7.33 (m, 3H), 4.86 (td, *J* = 10.9, 4.3 Hz, 1H), 2.08 (d, *J* = 11.9, 1H), 2.03-1.92 (m, 1H), 1.71 (d, *J* = 11.9, 2H), 1.56-1.43 (m, 2H), 1.16-1.02 (m, 2H), 0.97-0.84 (m, 7H), 0.80 (d, J = 6.9 Hz, 3H); ^{13}C NMR (100 MHz, $CDCl_3$): δ 154.0, 133.1, 130.6, 128.7, 120.0, 86.0, 81.2, 76.5, 47.0, 40.8, 34.3, 31.6, 26.3, 23.5, 22.1, 20.9, 16.4. HRMS (ESI^+) Calc. for $C_{19}H_{25}O_2$ [$M+H^+$] 284.1855, found: 285.1866. IR (neat): 2217, 1704, 1280, 1189, 1173, 756 cm^{-1}.

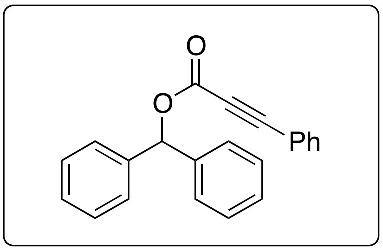

Benzhydryl 3-phenylpropiolate (30)

Purified by column chromatography (diethyl ether/Petroleum ether = 5: 100) to afford **30** as a white crystalline solid (0.1910 g, 61%). m.p. 92-94 °C. ^{1}H NMR (400 MHz, $CDCl_3$): δ 7.62-7.58 (m, 2H), 7.48-7.26 (m, 13H), 7.01 (s, 1H); ^{13}C NMR (100 MHz, $CDCl_3$): δ 153.3, 139.5, 133.2, 128.7, 128.3, 127.4, 119.8, 87.1, 80.8, 78.7. NMR data is consistent with literature values.[8] HRMS (ESI^+) Calc. for $C_{44}H_{32}O_4Na$[$2M+Na^+$] 647.2198, found: 647.2253. IR (neat): 2927, 2856, 2209, 1698, 1290, 1166 cm^{-1}. IR data is consistent with literature values.[8]

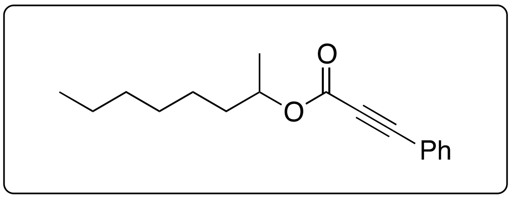

Octan-2-yl 3-phenylpropiolate (31)

Purified by column chromatography (diethyl ether/Petroleum ether = 5: 100) to afford **31** as a yellow oil (0.2066 g, 80%). ^{1}H NMR (400 MHz, $CDCl_3$): δ 7.62-7.56 (m, 2H), 7.49-7.40 (m, 1H), 7.40-7.33 (m, 2H), 5.11-5.01 (m, 1H), 1.76-1.48 (m, 2H), 1.40-1.21 (m, 11H), 0.89 (t, *J* = 6.8 Hz, 3H); ^{13}C NMR (100 MHz, $CDCl_3$): δ 154.0, 133.1, 130.6, 128.7, 120.0, 85.8, 81.2, 73.6, 36.0, 31.9, 29.2, 25.5, 22.7, 20.0, 14.2 HRMS (ESI^+) Calc. for $C_{17}H_{26}NO_2$ [$M+NH_4^+$] 276.1964, found: 276.1955. IR (neat): 3077, 3037, 2216, 1705, 1284, 1190, 1120 cm^{-1}.

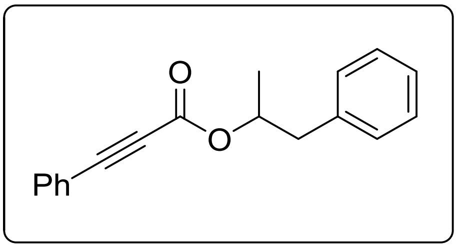

1-Phenylpropan-2-yl 3-phenylpropiolate (32)

Purified by flash column chromatography (diethyl ether/petroleum ether = 5: 100) to afford **32** as a pale yellow oil (0.2252g, 85 %). ^{1}H NMR (400 MHz, $CDCl_3$): δ 7.61-7.56 (m, 2H), 7.47-7.42 (m, 1H), 7.40-7.34 (m, 2H), 7.34-7.28 (m, 2H), 7.26-7.21 (m, 3H), 5.30-5.21 (dt, *J* = 13.0, 6.3 Hz, 1H), 3.11-3.00 (dd, *J* = 13.6, 6.3 Hz, 1H), 2.87-2.78 (dd, *J* = 13.6, 7.0 Hz, 1H), 1.32-1.28 (d, *J* = 6.3 Hz, 3H). ^{13}C NMR (100 MHz, $CDCl_3$): δ 153.8, 137.3, 133.2, 130.1, 129.6, 128.7, 126.9, 119.9, 86.2, 81.1, 73.9, 42.2, 19.4. HRMS (ESI^+) Calc. for $C_{18}H_{16}O_2Na$ [$M+Na^+$] 287.1048, found: 287.1038. IR (neat): 2218, 1700, 1282, 1206, 1187, 743, 686 cm^{-1}.

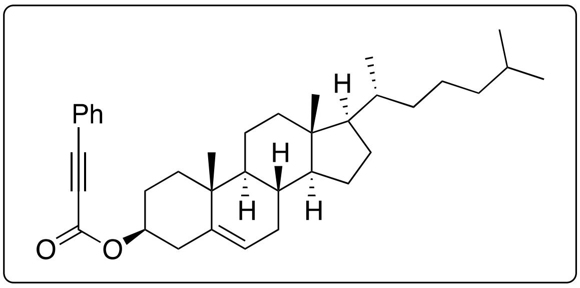

(3*S*,8*S*,9*S*,10*R*,13*R*,14*S*,17*R*)-10,13-Dimethyl-17-((*R*)-6-methylheptan-2-yl)-2,3,4,7,8,9,10,11,12,13,14,15,16,17-tetradecahydro-1*H*-cyclopenta[*a*]phenanthren-3-yl 3-phenylpropiolate (33)

Purified by column chromatography (diethyl ether/Petroleum ether = 5: 100) to afford **33** as a white crystalline solid (0.2286 g, 85%). m.p. 155-156 °C. ^{1}H NMR (400 MHz, $CDCl_3$): δ 7.60-7.58 (m, 2H), 7.48-7.41 (m, 1H), 7.41-7.33 (m, 2H), 5.42 (d, *J* = 5.0 Hz, 1H), 4.85-4.72 (m, 1H), 2.47-2.39 (m, 2H), 2.04-0.85 (m, 38H), 0.68 (s, 3H); ^{13}C NMR (100 MHz, $CDCl_3$): δ 153.7, 139.4, 133.1, 130.7, 128.7, 123.3, 119.9, 86.0, 81.2, 76.25, 56.8, 56.3, 50.2, 39.7, 39.9, 39.7, 38.1, 37.1, 36.3, 36.0, 32.1, 32.0, 28.4, 28.2, 27.8, 24.4, 24.0, 23.0, 22.7, 21.2, 19.5, 18.9, 12.0. NMR data is consistent with literature values.[9] HRMS (ESI$^+$) Calc. for $C_{72}H_{101}O_4$ [2M+H$^+$] 1029.76, found: 1029.77; $C_{72}H_{100}O_4Na$ [2M+Na$^+$] 1051.7520, found: 1051.7500. IR (neat): 2967, 2841, 2228, 1705, 1284, 1192, 1188 cm^{-1}. IR data is consistent with literature values.[10]

5 Characterization Data for synthesized Ligands and Isolated Pd(II) Complexes

Synthesis of (1,10-phenanthroline)Pd(OAc)$_2$

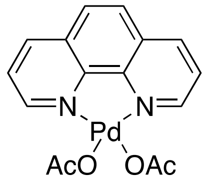

(1,10-Phenanthroline)Pd(OAc)$_2$ was synthesized using the method reported by Sheldon and coworkers.[11] A solution of 1,10-phenanthroline (0.2292 g, 1.272 mmol) in dichloromethane (4 mL) was added to a solution of Pd(OAc)$_2$ (0.2746 g, 1.223 mmol) in toluene (25 mL) at room temperature under N_2. The reaction mixture was stirred overnight, and then HPLC grade hexane was added to precipitate the complex. Then the yellow solid was filtered off and washed with diethyl ether and dried under vaccum. Yield: 0.4528 g, 92%.

^{1}H NMR (400 MHz, $CDCl_3$): δ 8.62-8.54 (m, 4H), 7.97 (s, 2H), 7.80 (dd, *J* = 8.2, 5.3 Hz, 2H), 2.21 (s, 6H); ^{13}C NMR (100 MHz, $CDCl_3$): δ 178.8, 150.6, 146.7, 138.8, 129.8, 127.3, 125.4, 23.5. NMR data is consistent with literature.[12] HRMS (ESI$^+$) Calc. for $C_{14}H_{11}N_2O_2Pd$ [M-OAc]$^+$ 344.9855, found: 344.9851.

Synthesis of (bathophenanthroline)Pd(OAc)$_2$

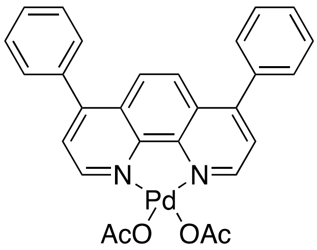

(bathophenanthroline)Pd(OAc)$_2$ was synthesized using the method reported by Sheldon and coworkers.[11] A solution of bathophenanthroline (0.1015 g, 0.3023 mmol) in dichloromethane (2 mL) was added to a solution of Pd(OAc)$_2$ (0.0656 g, 0.2922 mmol) in toluene (12 mL) and stirred at room temperature under a N_2 atmosphere overnight. HPLC grade hexane was then added to precipitate the complex. The yellow solid was filtered and washed with diethyl ether and dried under vacuum. Yield: 0.1514 g, 93%.

^{1}H NMR (400 MHz, $CDCl_3$): δ 8.71 (d, *J* = 5.5 Hz, 2H), 8.00 (s, 2H), 7.75 (d, *J* = 5.5 Hz, 2H), 6.64-7.57 (m, 6H), 7.56-7.49 (m, 4H), 2.23 (s, 6H); ^{13}C NMR (100 MHz, $CDCl_3$): δ 178.8, 152.0, 150.2, 147.6, 135.3, 130.3, 129.6, 129.5, 128.4, 125.6, 125.4, 23.6. NMR data is consistent with literature.[13] HRMS (ESI$^+$) Calc. for $C_{26}H_{19}N_2O_2Pd$ [M-OAc]$^+$ 497.0481, found: 497.0488

Synthesis of (2,2'-biquinoline)Pd(OAc)$_2$

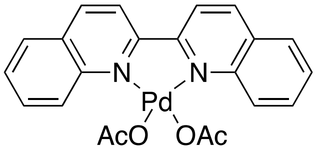

A solution of 2,2'-biquinoline (0.1282 g, 0.5 mmol) in dichloromethane (6 mL) was added to a solution of Pd(OAc)$_2$ (0.1123 g, 0.5 mmol) in toluene (10 mL) and stirred at room temperature under a N_2 atmosphere overnight. HPLC grade hexane was then added to precipitate the complex. The

yellow solid was filtered and washed with diethyl ether and dried under vacuum. Yield: 0.1793 g, 73%

^{1}H NMR (400 MHz, $CDCl_3$): δ 8.79 (d, *J* = 8.7 Hz, 2H), 8.65 (d, *J* = 8.7, 2H), 8.38 (d, *J* = 8.6 Hz, 2H), 7.87-7.80 (m, 2H), 7.67-7.58 (m, 4H), 2.03 (s, 6H); ^{13}C NMR (100 MHz, $CDCl_3$): δ 177.9, 158.1, 148.1, 142.1, 131.9, 129.3, 129.1, 128.0, 127.6, 120.5, 23.3. HRMS (ESI^+) Calc. for $C_{20}H_{15}N_2O_2Pd$ $[M-OAc]^+$ 421.0168, found: 421.0175.

Synthesis of (2-(2-pyridyl)benzoxazole)Pd(OAc)$_2$

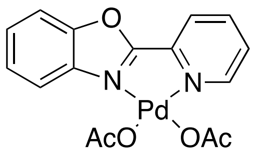

A solution of 2-(2-pyridyl)benzoxazole (0.0490 g, 0.25 mmol) in dichloromethane (2 mL) was added to a solution of $Pd(OAc)_2$ (0.0561 g, 0.25 mmol) in toluene (6 mL) and stirred at room temperature under a N_2 atmosphere overnight. HPLC grade hexane was then added to precipitate the complex. The yellow solid was filtered and washed with diethyl ether and dried under vacuum. Yield: 0.0749 g, 71%.

^{1}H NMR (400 MHz, $CDCl_3$): δ 8.47 (ddd, *J* = 5.5, 1.3, 0.6 Hz, 1H), 8.20 (td, *J* = 7.8, 1.5 Hz, 1H), 8.08 (ddd, *J* = 7.8, 1.3, 0.6 Hz, 1H), 7.78-7.73 (m, 1H), 7.72-7.65 (m, 2H), 7.64-7.50 (m, 2H), 2.20 (s, 3H), 2.16 (s, 3H); ^{13}C NMR (100 MHz, $CDCl_3$): δ 179.0, 178.8, 162.5, 151.8, 150.2, 143.8, 140.6, 136.5, 129.1, 128.9, 128.1, 123.6, 120.1, 112.0, 23.2, 22.8. HRMS (ESI^+) Calc. for $C_{14}H_{11}N_2O_3Pd$ $[M-OAc]^+$ 360.9804, found: 360.9829.

Synthesis of (1-benzyl-2-(2'-pyridyl)benzoimidazole)Pd(OAc)$_2$

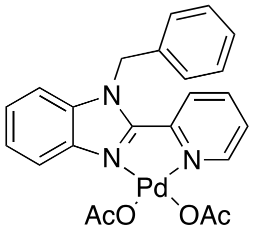

A solution of 1-benzyl-2-(2'-pyridyl)benzoimidazole (0.1425 g, 0.5 mmol) in dichloromethane (2 mL) was added to a solution of $Pd(OAc)_2$ (0.1123 g, 0.5 mmol) in toluene (10 mL) and stirred at room temperature under a N_2 atmosphere overnight. HPLC grade hexane was then added to precipitate the complex. The yellow solid was filtered and washed with diethyl ether and dried under vacuum. Yield: 0.2038, 80%.

^{1}H NMR (400 MHz, DMSO- d^6): δ 8.29-8.22 (m, 2H), 8.15 (d, *J* = 8.0 Hz, 1H), 7.94 (dd, J = 6.9, 1.9 Hz, 1H), 7.78-7.67 (m, 2H), 7.58-7.49 (m, 2H), 7.40-7.29 (m, 3H), 7.20-7.15 (m, 2H), 6.16 (s, 2H), 1.98 (s, 3H), 1.92 (s, 3H). HRMS (ESI$^+$) Calc. for $C_{21}H_{18}N_3O_2Pd$ [M-OAc]$^+$ 450.0433, found: 450.0524.

Synthesis of (2,2'-bipyridine)Pd(OAc)$_2$

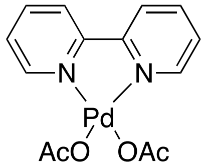

A solution of 2,2'-bipyridine (0.0781 g, 0.5 mmol) in dichloromethane (2 mL) was added to a solution of $Pd(OAc)_2$ (0.1123 g, 0.5 mmol) in toluene (10 mL) and stirred at room temperature under a N_2 atmosphere overnight. HPLC grade hexane was then added to precipitate the complex. The yellow solid was filtered and washed with diethyl ether and dried under vacuum. Yield: 0.1840 g, 97%.

^{1}H NMR (400 MHz, $CDCl_3$): δ 8.39 (d, *J* = 7.8, 2H), 8.19 (dd, J = 5.6, 1.3 Hz, 2H), 8.11 (td, *J* = 7.9, 1.6 Hz, 2H), 7.38 (dt, 7.9, 5.6, 1.3 Hz, 2H), 2.14 (s, 6H); ^{13}C NMR (100 MHz, $CDCl_3$): δ 178.5, 155.3, 150.0, 140.4, 126.3, 123.2, 23.4. HRMS (ESI$^+$) Calc. for $C_{12}H_{11}N_2O_2Pd$ [M-OAc]$^+$ 320.9855, found: 320.9877.

Synthesis of (Neocuproine)Pd(OAc)$_2$

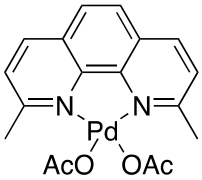

(Neocuproine)Pd(OAc)$_2$ was synthesized using a method reported by Sheldon and co-workers.[11] A solution of neocuproine (0.25 g, 1.1 mmol) in dichloromethane (4 mL) was added to a solution of Pd(OAc)$_2$ (0.2240 g, 1 mmol) in toluene (10 mL) and stirred at room temperature under a N_2 atmosphere overnight. HPLC grade hexane was then added to precipitate the complex. The yellow solid was filtered and washed with diethyl ether and dried under vacuum. Yield: 0.3338 g, 75%.

^{1}H NMR (400 MHz, $CDCl_3$): δ 8.34 (d, J = 8.4 Hz, 2H), 7.85 (s, 2H), 7.45 (d, J = 8.4 Hz, 2H), 2.92 (s, 6H), 2.04 (s, 6H); ^{13}C NMR (100 MHz, $CDCl_3$): δ 178.7, 165.6, 147.6, 138.4, 128.1, 127.0, 126.4, 24.7, 23.1. NMR data is consistent with literature values.[11] HRMS (ESI$^+$) Calc. for $C_{16}H_{15}N_2O_2Pd$ [M-OAc]$^+$ 373.0168, found: 373.0138.

Synthesis of (Quinox)Pd(OAc)$_2$

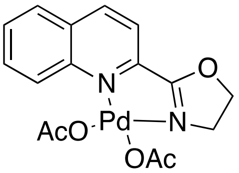

A solution of Quinox (0.0495 g, 0.25 mmol) in dichloromethane (2 mL) was added to a solution of Pd(OAc)$_2$ (0.0562 g, 0.25 mmol) in dichloromethane (6 mL) and stirred at room temperature under a N_2 atmosphere overnight. HPLC grade hexane was then added to precipitate the complex. The yellow solid was filtered and washed with diethyl ether and dried under vacuum. Yield: 0.0673 g, 64%.

^{1}H NMR (400 MHz, $CDCl_3$): δ 9.03 (d, *J* = 8.3 Hz, 1H), 8.56 (d, *J* = 8.3 1H), 7.94-7.83 (m, 2H), 7.79-7.73 (m, 2H), 4.90 (t, *J* = 9.9 Hz, 2H), 4.13 (t, *J* = 9.9 Hz, 2H), 2.09 (d, *J* = 4.7 Hz, 6H); ^{13}C NMR (100 MHz, $CDCl_3$): δ 178.8, 152.0, 150.2, 147.6, 135.3, 130.3, 129.6, 129.5, 129.2, 128.4, 128.3, 125.6, 125.4, 23.6. HRMS (ESI^+) Calc. for $C_{14}H_{13}N_2O_3Pd$ $[M\text{-}OAc]^+$ 362.9961, found: 362.9949.

Synthesis of (Phen)PdI$_2$

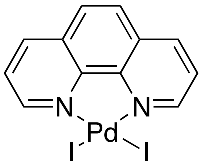

This compound was synthesized by a method previously reported.[14] To 0.111 g (0.50 mmol) of Pd(OAc)2 dissolved in EtOH (10 mL) were added KI (0.2 g, 1.2 mmol; I/Pd = 2.4, dissolved in 10 mL EtOH) and phenanthroline (0.10 g, 0.5 mmol, dissolved in 10 mL of EtOH). After 30 min string, the resulting precipitated pink-brown product was filtered, washed with H_2O/EtOH mixture (1:1, v/v), and dried in *vacuo*. Yield: 20% 0.0538 g. Elemental Analysis: Predicted: C, 26.67; H, 1.49; N, 5.18; Found: C, 28.30; H, 1.66; N, 5.12.

Synthesis of 1,1’-Dimethyl-3,3’-methylenediimidazolium dibromide

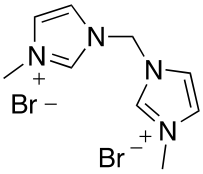

1-Methylimidazole (2.0 g, 24.4 mmol) and dibromomethane (1.5 g, 8.6 mmol) was added to an oven dried two-necked round bottomed flask and stirred magnetically under reflux for 48 hours. After cooling, the resulting light orange precipitate was filtered under suction, washed with dry tetrahydrofuran and recrystallised from methanol/tetrahydrofuran, then dried in vacuo to give white crystals. Yield = 2.68 g, 88 %.

^{1}H NMR (400 MHz, DMSO- d^6): δ 9.55 (s, 2H), 8.08 (t, *J* = 1.8 Hz, 2H), 7.81 (t, *J* = 1.8 Hz, 2H), 6.76 (s, 2H), 3.90 (s, 6H); ^{13}C NMR (100 MHz,

DMSO- d^6): δ 138.0, 124.3, 121.9, 57.8, 36.2. NMR data is consistent with literature data.[15] HRMS (ESI^+) Calc. for $C_9H_{13}N_4$ [M – 2Br – H]$^+$ 177.1140, found: 177.1130.

Synthesis of (1,1'-Dimethyl-3,3'-methylenediimidazol-2,2'-diylidene) palladium(II) dibromide - [PdBr$_2$(*cis*-CH$_2${NC(H)=C(H)N(Me)C}$_2$)]

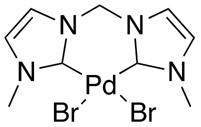

Prepared by an adapted procedure from Slootweg and Chen:[15] To a solution of $Pd(OAc)_2$ (0.3320 g, 1.47 mmol) and dimethylsulfoxide in an oven dried round bottomed flask, the diimidazolium bromide salt (0.5050 g, 1.49 mmol) was added. The mixture was magnetically stirred for four hours at room temperature, heated to 40 °C for 14 hours and then lastly heated to 120 °C for a further two hours, all of which was carried out under a dry N_2 gas atmosphere. The solution was subsequently cooled, reduced in volume *in vacuo* to a few milliliters and precipitated with acetonitrile (10 mL) to give a light yellow powder after filtration. Yield: 0.4300 g, 66%.

^{1}H NMR (400 MHz, DMSO- d^6): δ 7.60 (s, 2H), 7.34 (s, 2H), 6.28 (s, 2H), 3.91 (s, 6H). NMR data is consistent with previously reported values.[15] HRMS (ESI^+) Calc. for $C_9H_{12}BrN_4Pd$ [M – Br^-]$^+$ 362.9280, found 362.9273.

Synthesis of (1,1'-Dimethyl-3,3'-methylenediimidazolin-2,2'-diylidene)palladium(II) diacetate - [Pd(OAc)$_2$(*cis*-CH$_2${NC(H)=C(H)N(Me)C}$_2$)]

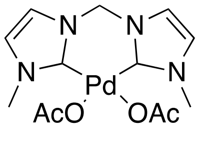

Prepared by an adapted procedure from Slootweg and Chen:[15] To a solution of [PdBr$_2$(*cis*-CH$_2${NC(H)=C(H)N(Me)C}$_2$)] (0.2000 g, 0.45 mmol) in acetonitrile (15 mL), AgOAc (0.1509 g, 0.9 mmol) was added. The mixture was magnetically stirred at 60 °C overnight under a dry N_2 gas atmosphere in the exclusion of light. Once cooled, the mixture was filtered

through Celite under suction and eluted with additional dry acetonitrile to remove the precipitated AgBr. Then, the filtrate was evaporated to dryness *in vacuo* to yield a white powder. Yield: 0.1528 g, 85%.

^{1}H NMR (400 MHz, DMSO- d^6): δ 7.54 (d, 2H, *J* = 2.0 Hz), 7.26 (d, 2H, *J* = 2.0 Hz), 6.17 (s, 2H), 3.74 (s, 6H), 1.73 (s, 6H). NMR data is consistent with literature values.[15] HRMS (ESI$^+$) Calc. for $C_{11}H_{17}N_4O_2Pd$ [M-OAc]$^+$ 343.0386, found: 343.0216.

Synthesis of (Phenanthroline)Pd($CF_3COO)_2$

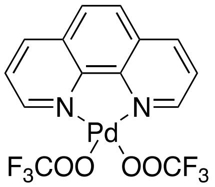

A solution of 1,10-phenanthroline (0.573 g, 0.318 mmol) in dichloromethane (2 mL) was added to a solution of Pd($CF_3COO)_2$ (0.1060g, 0.318 mmol) in acetonitrile (4 mL) at room temperature under N_2. The reaction mixture was stirred overnight, and then HPLC grade hexane was added to precipitate the complex. Then the yellow solid was filtered off and washed with diethyl ether and dried under vacuum. Yield: 0.1310 g, 80%.

^{1}H NMR (400 MHz, DMSO- d^6): δ 9.06 (dd, *J* = 8.3, 1.2 Hz, 2H), 8.64-8.37 (m, 2H), 8.33 (s, 2H), 8.15 (dd, *J* = 8.3, 5.4 Hz, 2H); ^{19}F NMR (376 MHz, DMSO- d^6): δ 73.29 (s, 6F). NMR data is consistent with previously published data.[13]

Synthesis of (1,10-phenanthroline)Pd(MeCN)$_2$(OTf)$_2$

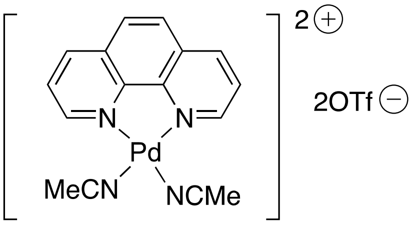

(1,10- phenanthroline)Pd(MeCN)$_2$(OTf)$_2$ was synthesized using a method from the literature:[16] To a slurry of (1,10-phenanthroline)Pd(OAc)$_2$ (0.2361 g, 0.5892 mmol) in acetonitrile (1 mL) was added a solution of triflic acid in acetonitrile (0.33 M, 4 mL, 2.5 equiv). The solution was stirred for 1 hour at room temperature and then precipitated by the addition of diethyl ether to give a yellow solid. The crude product was filtered and then re-dissolved in acetonitrile (1 mL) and a solution of triflic acid in acetonitrile (0.33 M, 4 mL 2.5 equiv) was added again. After stirring for 1 hour, diethyl ether was once again used to precipitate the complex. The light yellow solid was filtered, washed with diethyl ether (80 mL) and dried under vaccum. Yield: 0.3377 g, 86%.

^{1}H-NMR (400 MHz, DMSO-d$_6$): δ (ppm) 2.03 (s, 6H), 8.10 (dd, J = 8.2, 5.4 Hz 2H), 8.31 (s, 2H), 8.66 (d, J = 4.8 Hz, 2H), 9.03 (d, J = 8.3 Hz, 2H).
^{13}C NMR (100 MHz, DMSO-d$_6$): δ (ppm) 1.1, 118.1, 120.7 (q, J = 320.1Hz, $CF_3SO_3^-$), 126.1, 127.8, 130.5, 141.3, 146.6, 149.7. ESI-MS: [M-MeCN - OTf]$^+$: $C_{15}H_{11}F_3O_3N_3PdS$, calcd m/z 475.9508, found 475.9655; [M-2MeCN-OTf]$^+$: $C_{13}H_8F_3N_2O_3PdS$, calcd m/z 434.9242, found 434.9938; [OTf]-:$CF_3SO_3^-$, calcd m/z 148.9520, found 148.9687.

Synthesis of 2-(2-pyridyl)benzoxazole

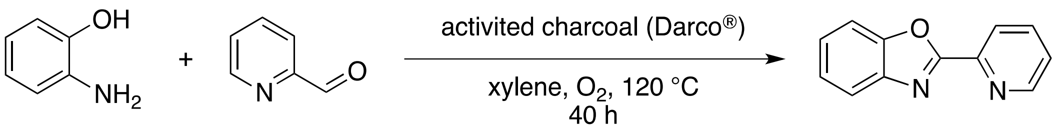

The 2-(2-pyridyl)benzoxazole was prepared using the method developed by Hayashi and coworkers.[17,18] A mixture of 2-aminophenol (1 g, 9.1633

mmol), 2-pyridylaldehyde (1.15 g, 10.74 mmol), activated charcoal (Darco®, -100 mesh particle size, powder, 1 g) and xylene (20 mL) were placed in a 100 mL round bottom flask. The flask was connected to a water cooled condenser, which had rubber septum and a 1 L balloon filled with O_2 on the top. The reaction mixture was heated to 120 °C for 40 hours (note that it has previously been discussed by Hayashi and coworkers that shorter reaction times are possible depending on the type of activated carbon used). The mixture was filtered using Celite and washed with methanol. The filtrate was concentrated and then purified by flash chromatography twice (ethyl acetate: hexane = 1:2, and then ethyl acetate: dichloromethane = 1:20). The product is a light yellow crystalline solid (Yield: 1.2738 g, 71%).

^{1}H-NMR (400 MHz, DMSO-d_6): δ (ppm) 7.45-7.53 (m, 2H), 7.65 (dd, *J* =7.2, 4.8 Hz, 1H), 7.86 – 7.89 (m, 2H), 8.08 (ddd, *J* = 9.0, 6.8, 1.6, 1H), 8.36 (d, *J* = 8.0 Hz, 1H), 8.81 (d, J = 4.8 Hz, 1H). ^{13}C NMR (100 MHz, DMSO-d_6): δ (ppm) 111.3, 120.3, 123.6, 125.1, 126.2, 137.7, 141.2, 145.2, 150.2, 150.4, 161.3. NMR Data is consistent with literature values.[17,18]

Synthesis of 1-benzyl-2-(2-pyridyl)benzoimidazole

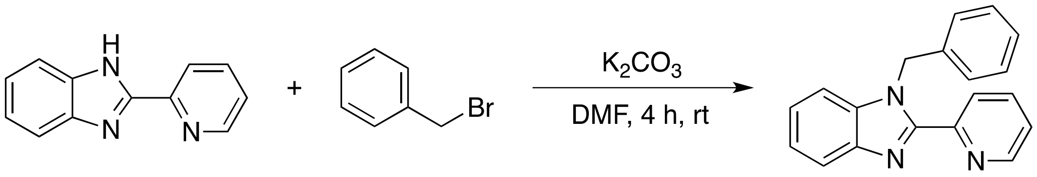

The 1-benzyl-2-(2-pyridyl)benzoimidazole was prepared using the method developed by Diau and coworkers.[19] 2-(2-pyridyl)benzimidazole (0.4375 g, 2.24 mmol) and K_2CO_3 (0.4960 g, 3.6 mmol) were dissolved in DMF (5 mL) and stirred for 30 min; (bromomethyl)benzene (0.5011 g, 3 mmol) was added to the reaction mixture that was then stirred at room temperature for 4 h. After evaporation of the solvent under reduced pressure, H_2O (25 mL) and ethyl acetate (30 mL) were added. The organic layer was separated and the aqueous phase extracted further with ethyl acetate (30 mL) twice. The organic layers were combined and dried over $MgSO_4$. The crude

product was purified using column chromatography with ethyl acetate and hexane (1:3) as the eluent. The product is a light purple crystalline solid (0.5912 g, 92.4%).

^{1}H-NMR (400 MHz, DMSO-d_6): δ (ppm) 6.23 (s, 2H), 7.13-7.29 (m, 7H), 7.51 (m, 1H), 7.56 (m, 1H), 7.75 (m, 1H), 8.01 (ddd, *J* = 8.0, 8.0, 1.6 Hz, 1H), 8.38 (d, *J* = 7.6 Hz, 1H), 8.70 (d, *J* = 4.0 Hz, 1H). ^{13}C NMR (100 MHz, DMSO-d_6): δ (ppm) 47.9, 111.3, 119.6, 122.6, 123.4, 124.4, 126.7, 127.2, 128.5, 136.5, 137.50, 137.7, 142.2, 148.8, 149.2, 150.0. NMR data is consistent with literature values.[19]

Synthesis of 1,2-bis[(2,6-diisopropylphenyl)imino]acenaphthene (dpp-BIAN)

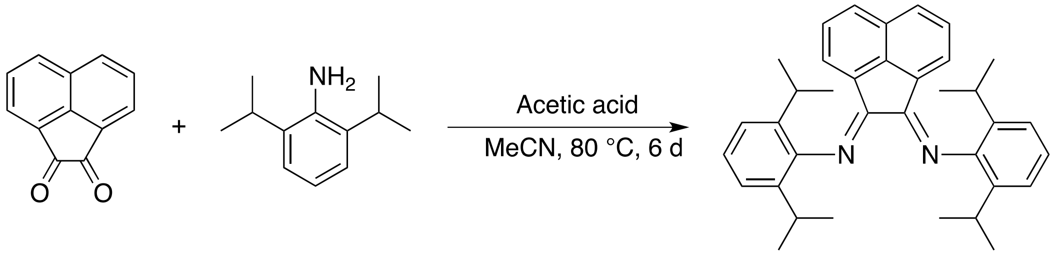

In an oven dried round bottomed flask, with a magnetic stirrer bar, acenaphthenequinone (0.551 g, 3.02 mmol) and 2,6-diisopropylaniline (1.354 g, 7.64 mmol) were mixed in dry acetonitrile (40 mL) and glacial acetic acid (2 mL). The mixture was heated to 80 °C and stirred under reflux for 6 days. The solution was then cooled, filtered under vacuum and the retentate washed with *n*-hexane, before drying overnight *in vacuo* to yield a yellow powder. (Yield = 1.315 g, 87%).

^{1}H-NMR (400 MHz, $CDCl_3$): δ (ppm) 7.88 (d, 2H, *J* = 8.4 Hz), 7.37 (t, 2H, *J* = 7.7 Hz), 7.27 (s, 6H), 6.64 (d, 2H, *J* = 7.1 Hz), 3.15 – 2.93 (m, 4H), 1.24 (d, 12H, *J* =6.9 Hz), 0.98 (d, 12H, *J* = 6.9 Hz). ^{13}C NMR (100 MHz, $CDCl_3$): δ (ppm) 161.1, 147.6, 141.0, 135.6, 131.3, 129.7, 129.0, 128.0, 124.4, 123.6, 123.5, 28.8, 23.6, 23.3. NMR analysis is in agreement with literature data.[20]

6 Example of IR Spectra

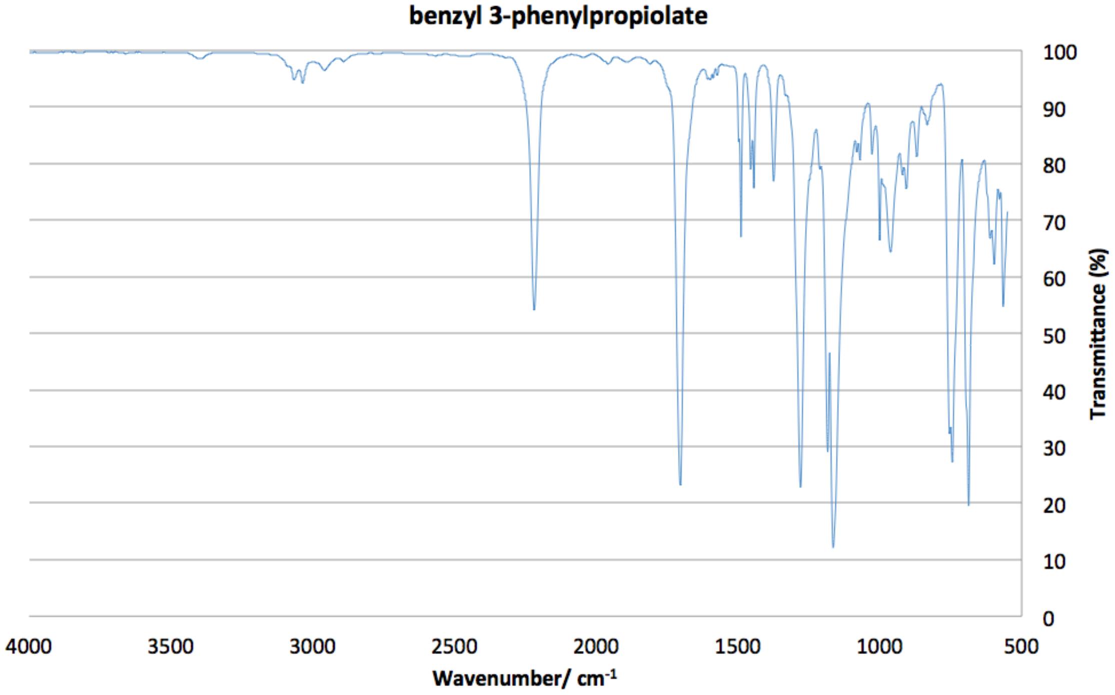

7 NMR Spectra

^{1}H NMR spectra were recorded on a Bruker AVX400 (400 MHz) spectrometer at ambient temperature. ^{13}C NMR spectra were recorded on a Bruker AVX400 (100 MHz) spectrometer at ambient temperature.

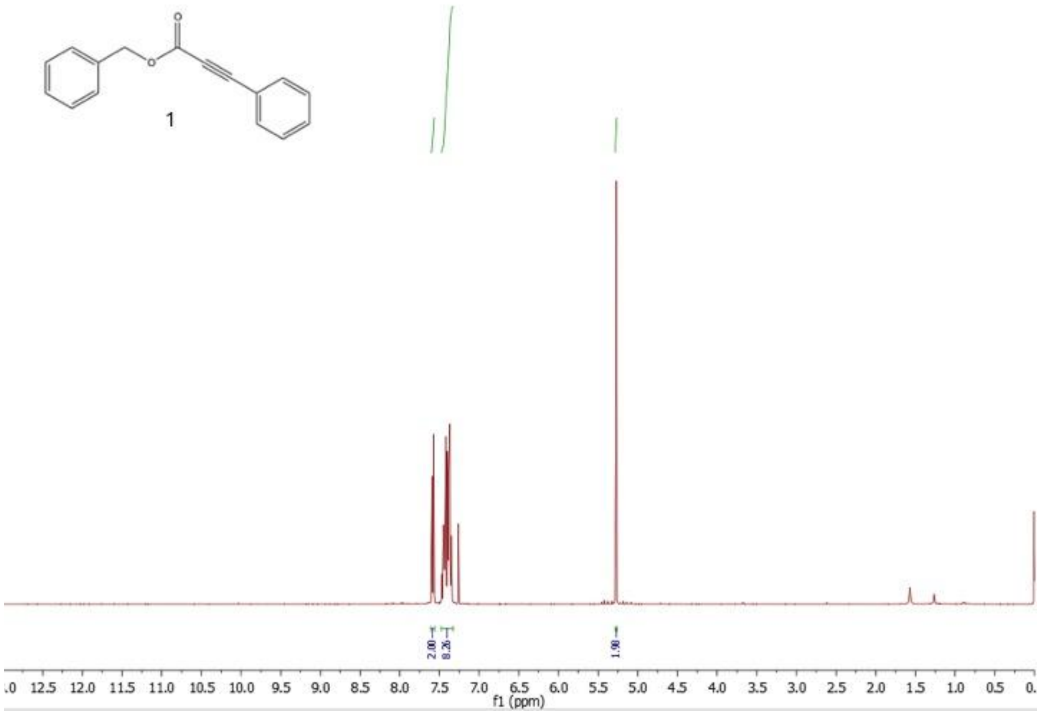

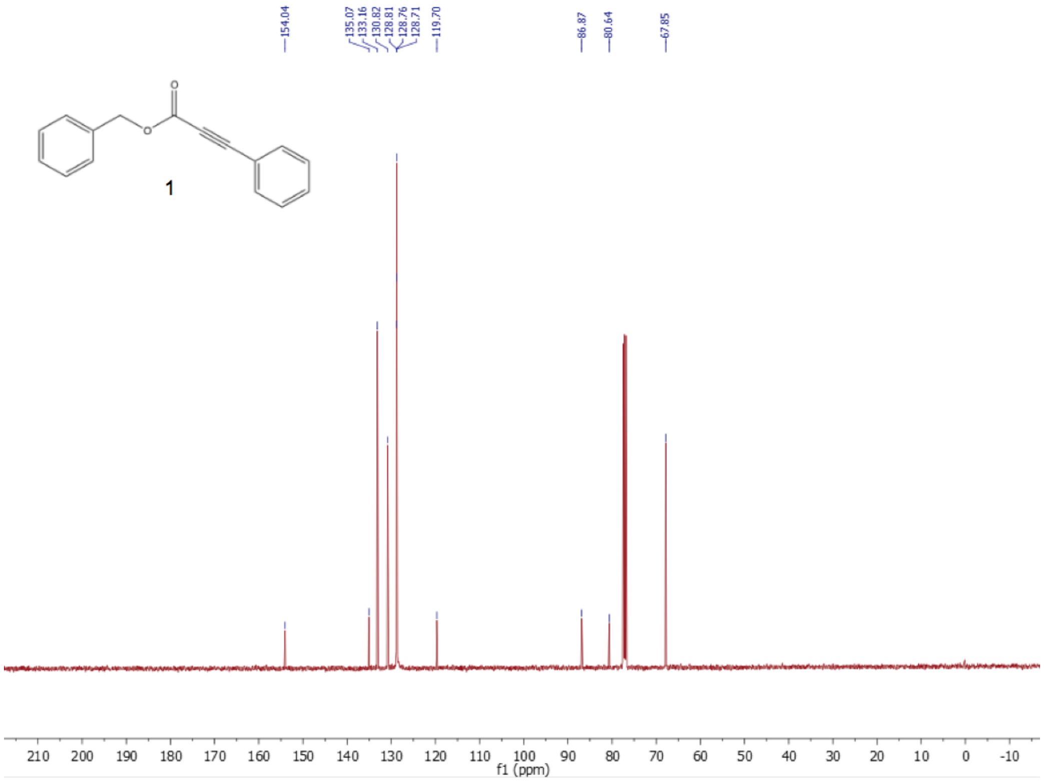

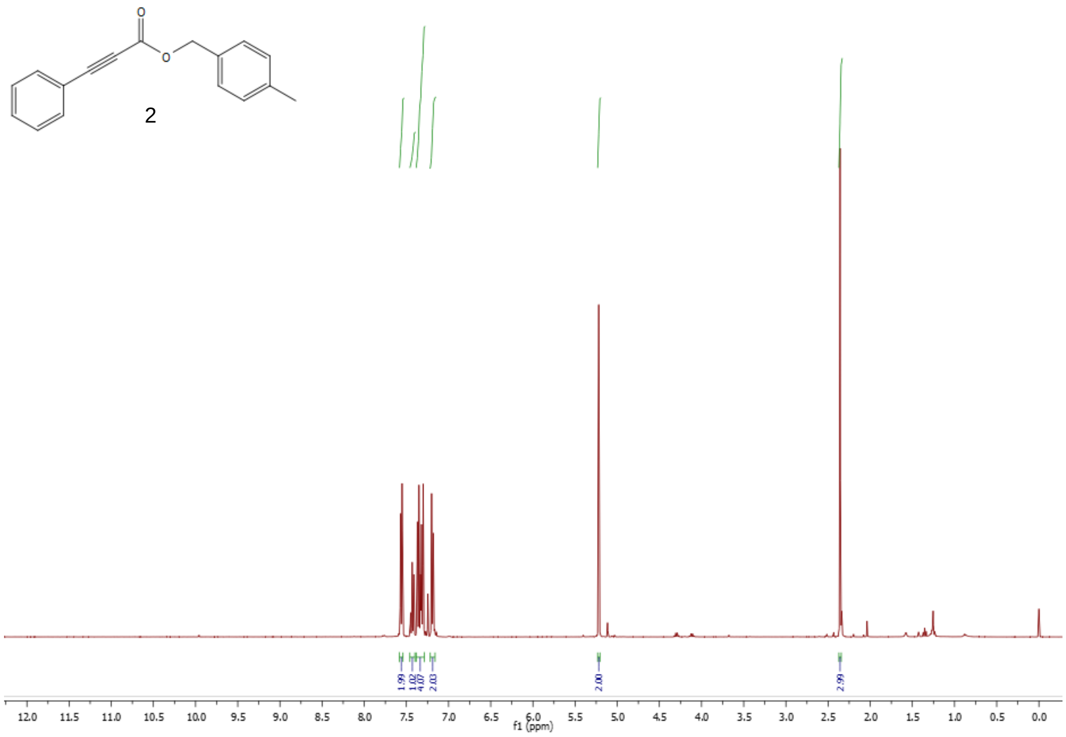

O
O
2
1.99
1.02
4.07
2.03
2.00
2.99
12.0 11.5 11.0 10.5 10.0 9.5 9.0 8.5 8.0 7.5 7.0 6.5 6.0 5.5 5.0 4.5 4.0 3.5 3.0 2.5 2.0 1.5 1.0 0.5 0.0
f1 (ppm)

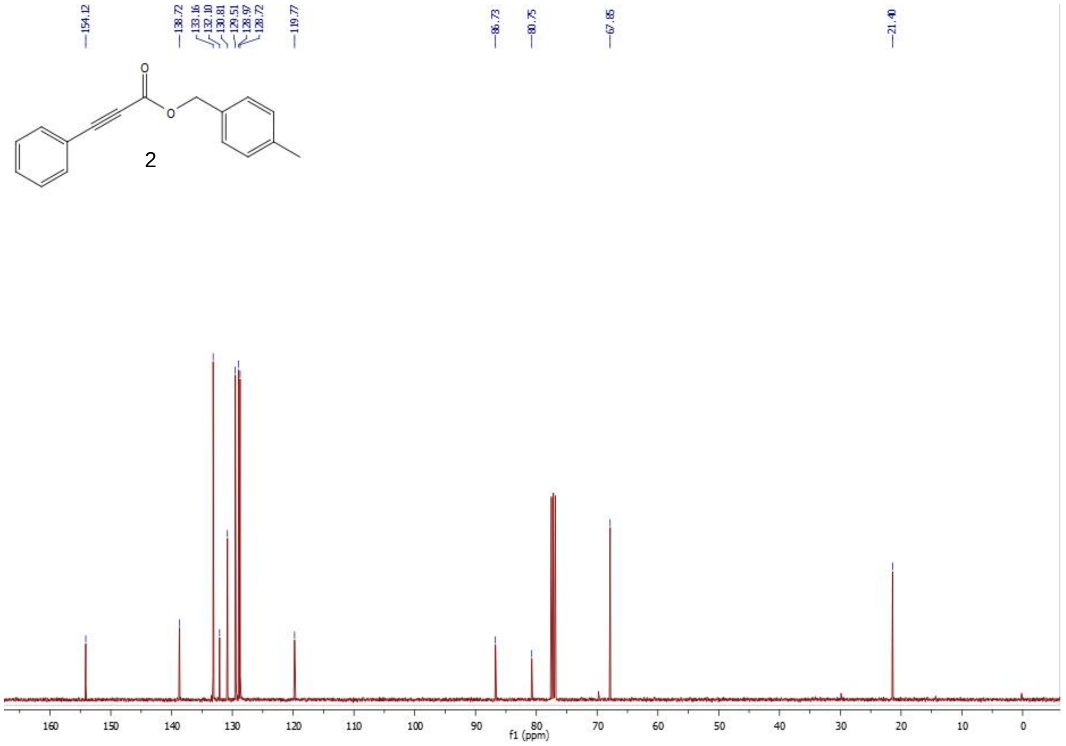

154.12
138.72
133.16
132.10
130.81
129.51
128.97
128.72
119.77
86.73
80.75
67.85
21.40
O
O
2
160 150 140 130 120 110 100 90 80 70 60 50 40 30 20 10 0
f1 (ppm)

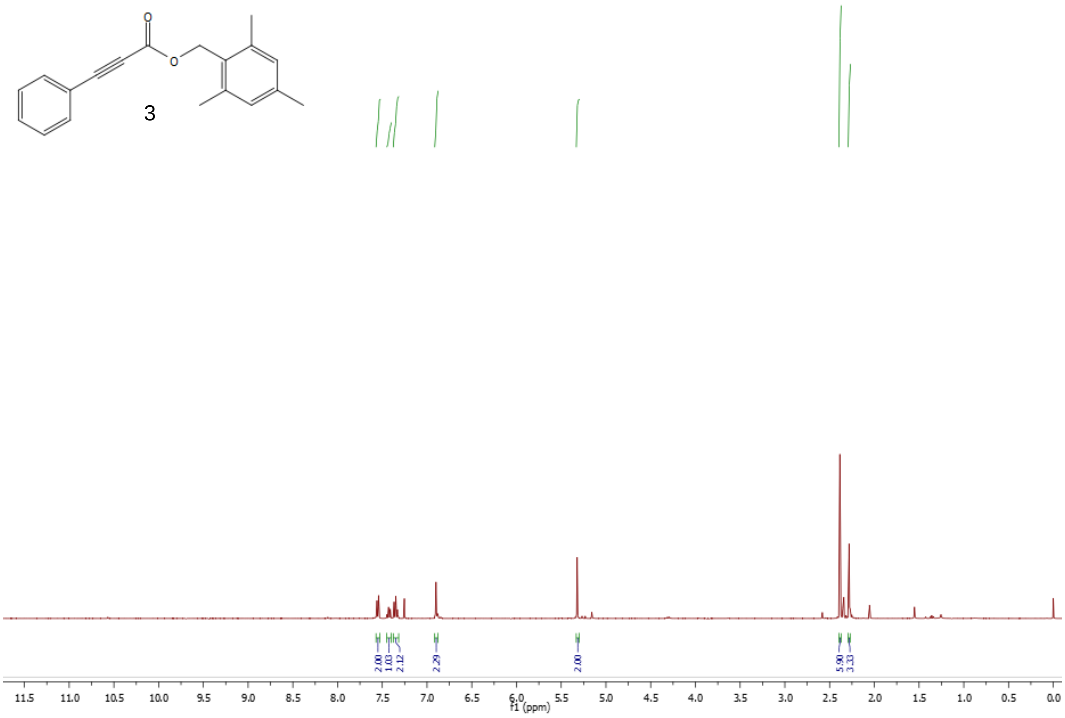
O
O
3
2.00
1.03
2.12
2.29
2.00
5.90
3.33
f1 (ppm)

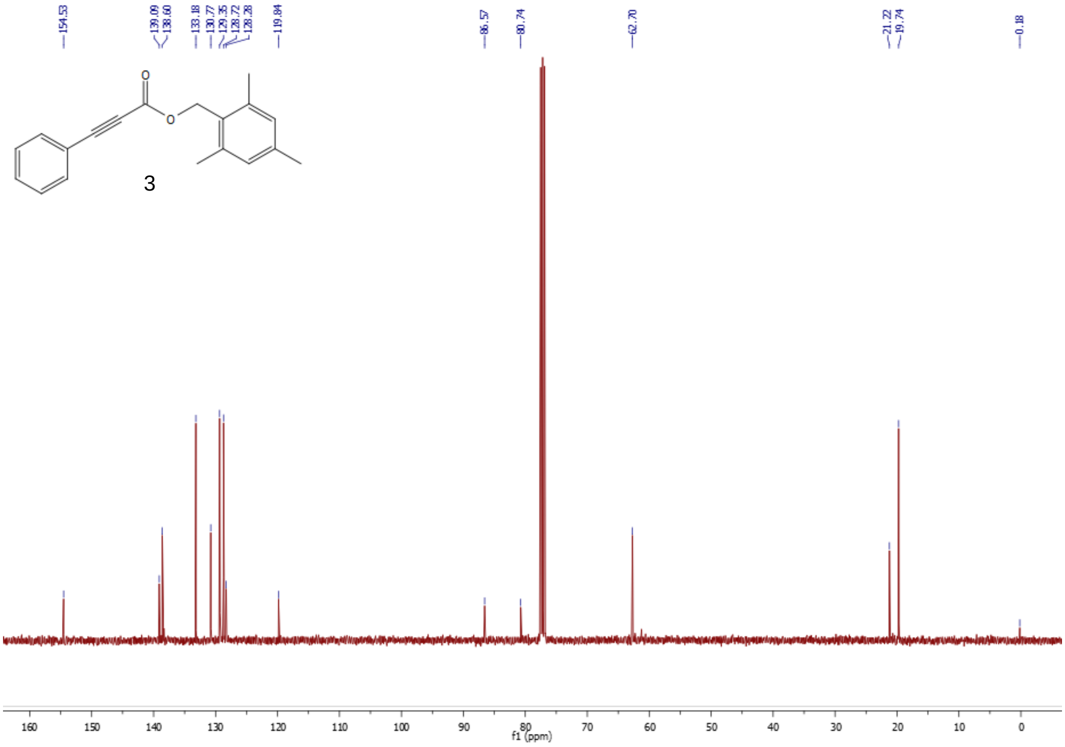
154.53
139.09
138.60
133.18
130.77
129.35
128.72
128.28
119.84
86.57
80.74
62.70
21.22
19.74
0.18
O
O
3
f1 (ppm)

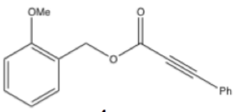
OMe
O
O
Ph

4

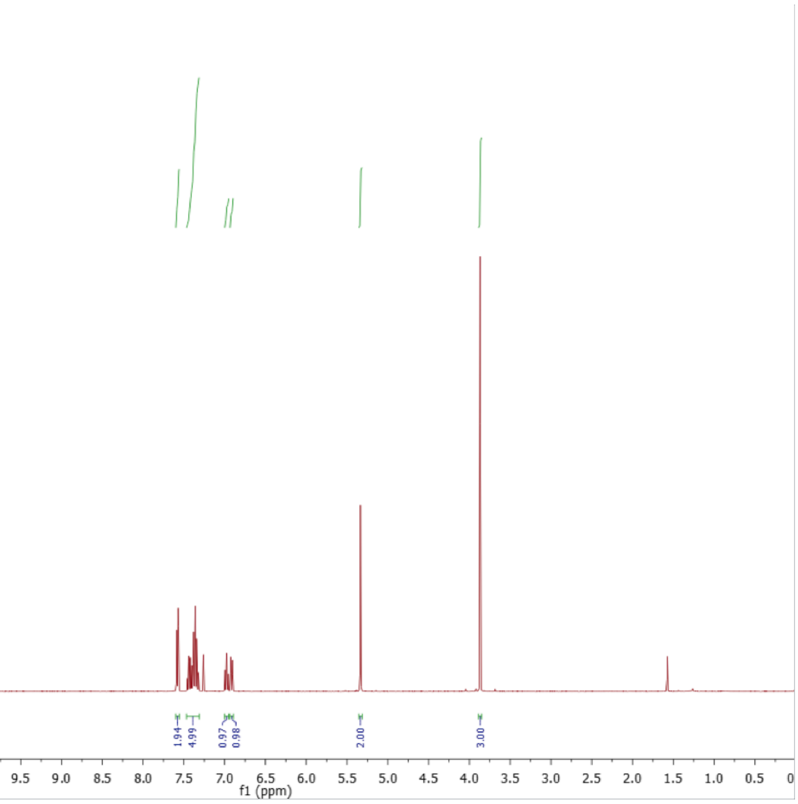
1.94
4.99
0.97
0.98
2.00
3.00
f1 (ppm)

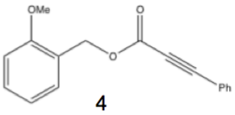
OMe
O
O
Ph

4

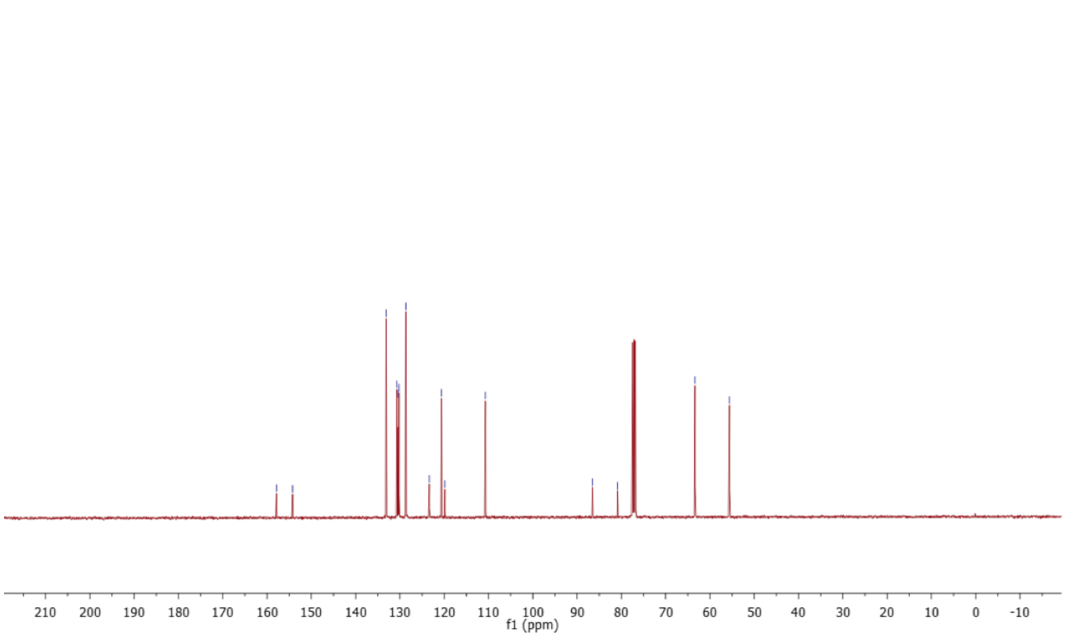
157.86
154.21
133.12
130.70
130.40
130.20
128.67
123.37
120.62
119.85
110.69
86.51
80.86
63.37
55.61
f1 (ppm)

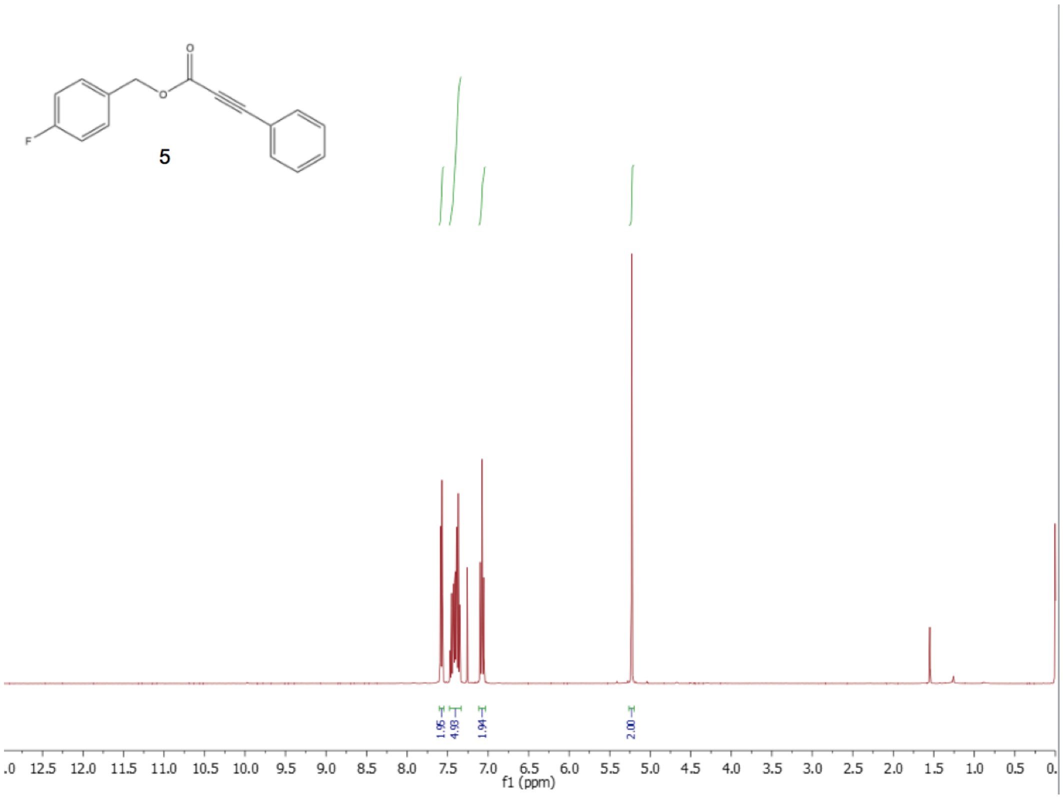
5
F
O
O
1.95
4.93
1.94
2.00
f1 (ppm)

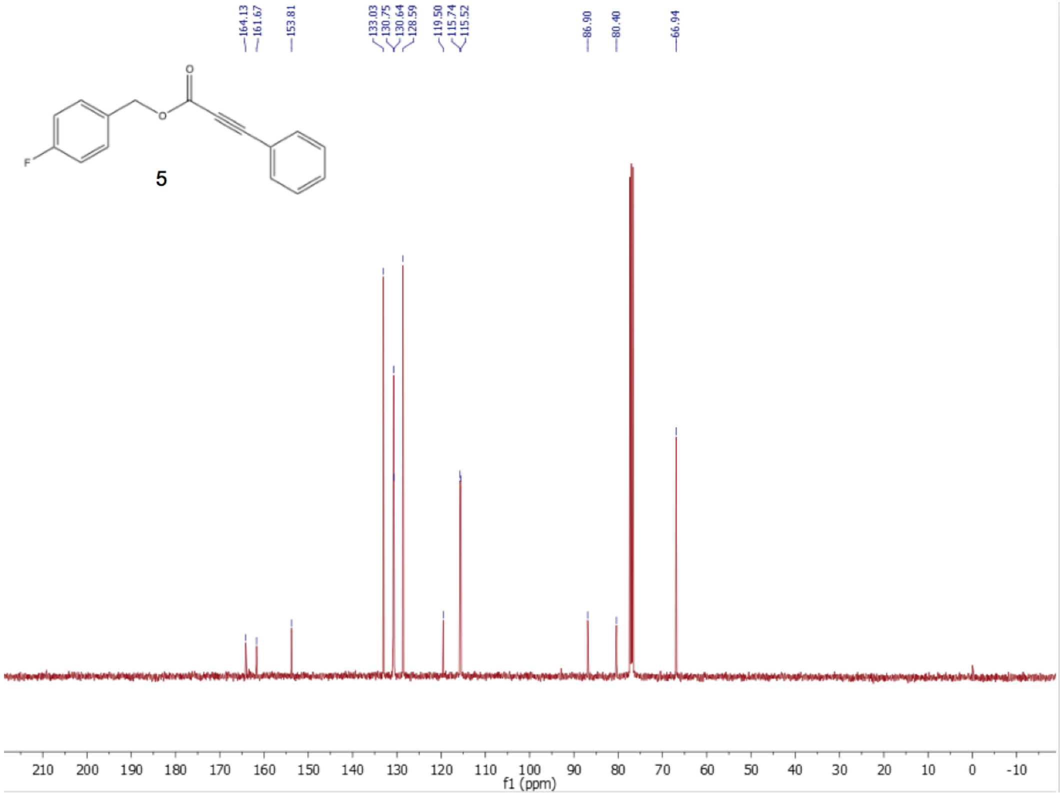
164.13
161.67
153.81
133.03
130.75
130.64
128.59
119.50
115.74
115.52
86.90
80.40
66.94
5
F
O
O
f1 (ppm)

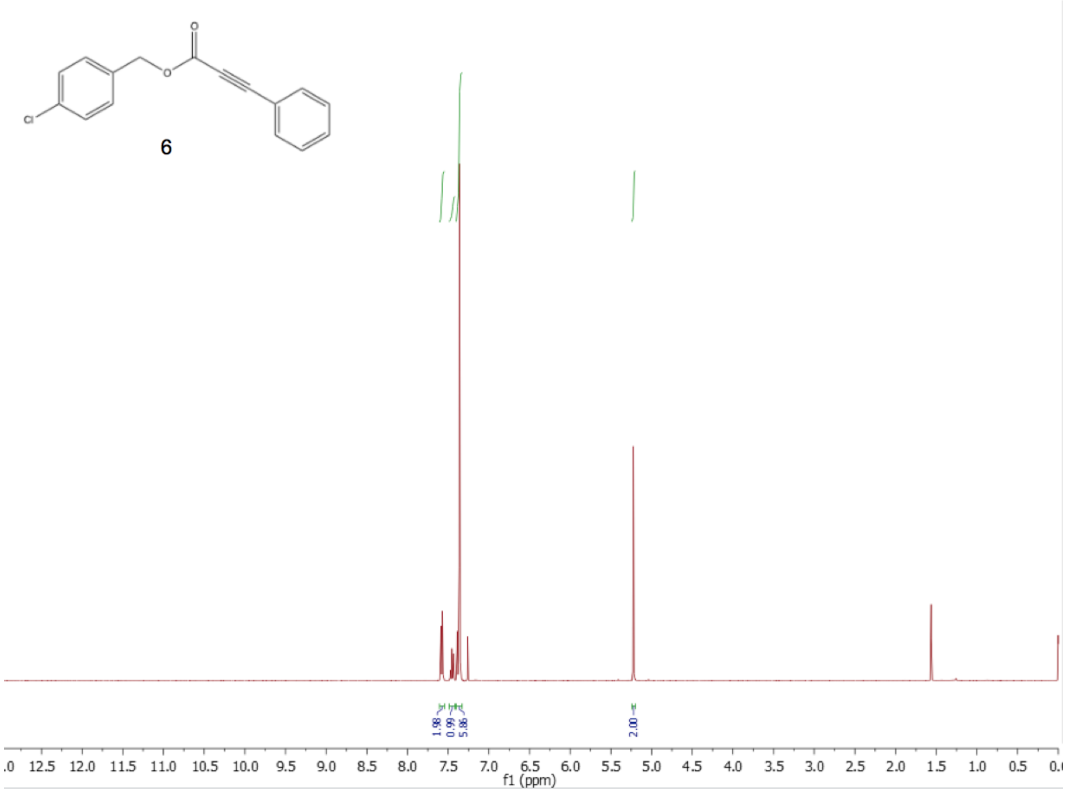
6
Cl
O
1.98
0.99
5.86
2.00
f1 (ppm)

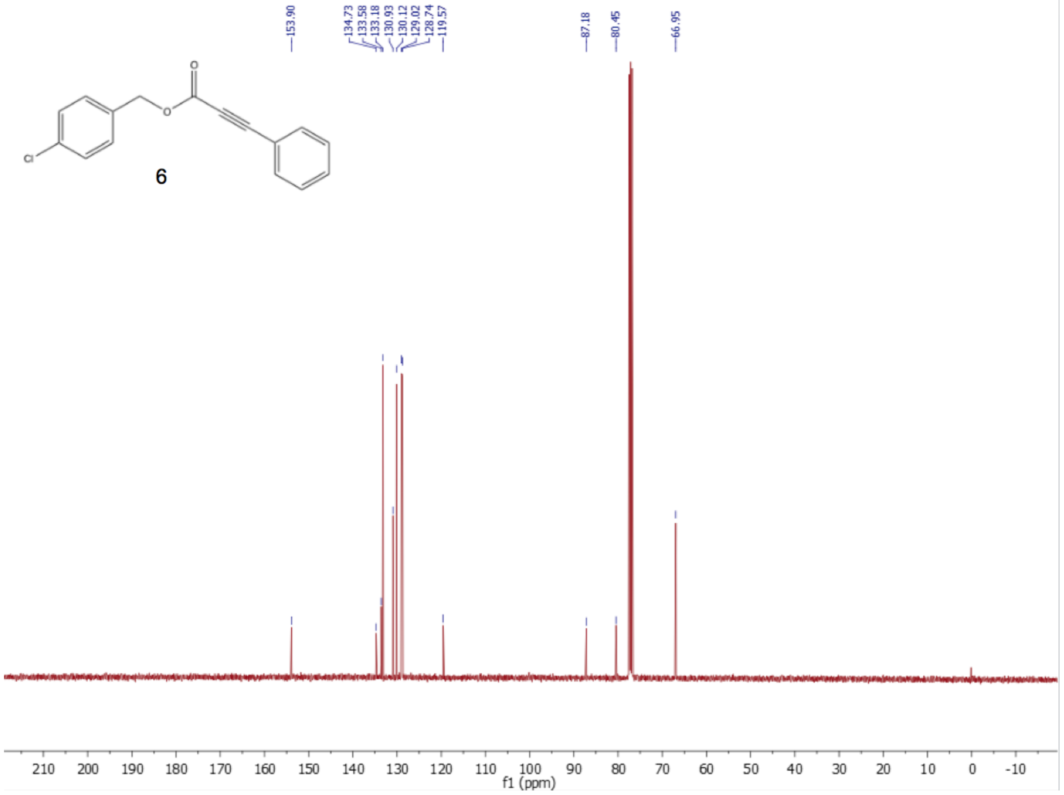
153.90
134.73
133.58
133.18
130.93
130.12
129.02
128.74
119.57
87.18
80.45
66.95
6
Cl
O
f1 (ppm)

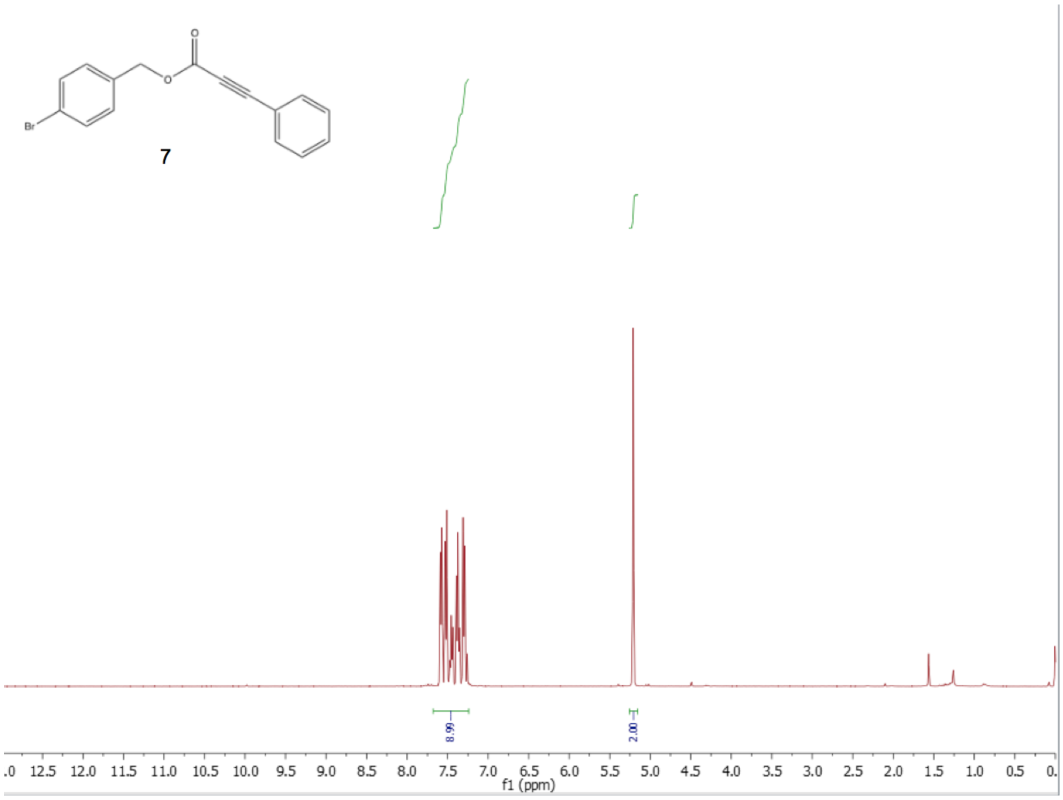

Br
O
O
7
8.99
2.00
f1 (ppm)

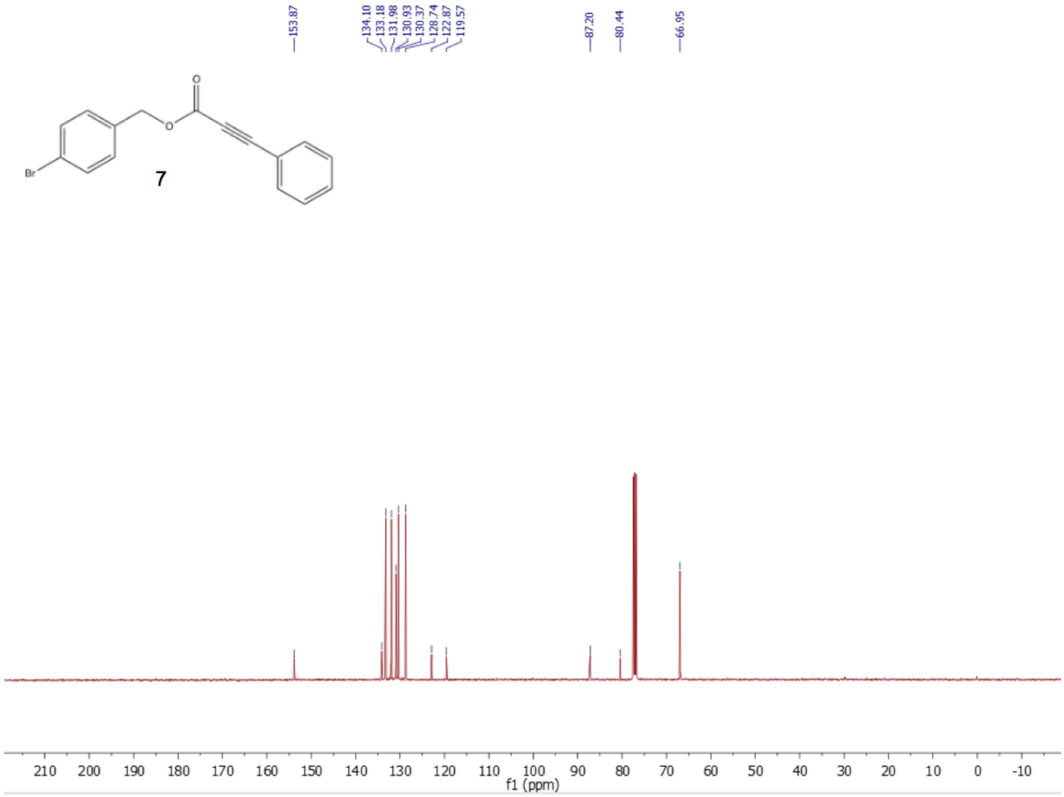

153.87
134.10
133.18
131.98
130.93
130.37
128.74
122.87
119.57
87.20
80.44
66.95
Br
O
O
7
f1 (ppm)

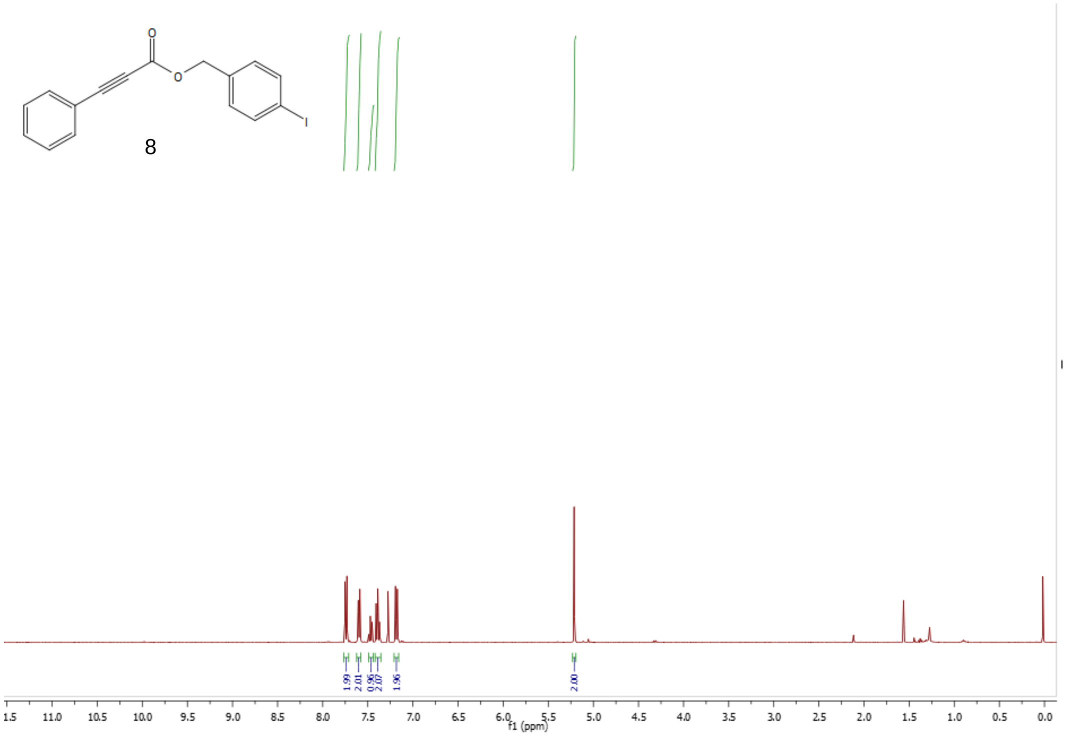
8
1.99
2.01
0.96
2.07
1.96
2.00
f1 (ppm)

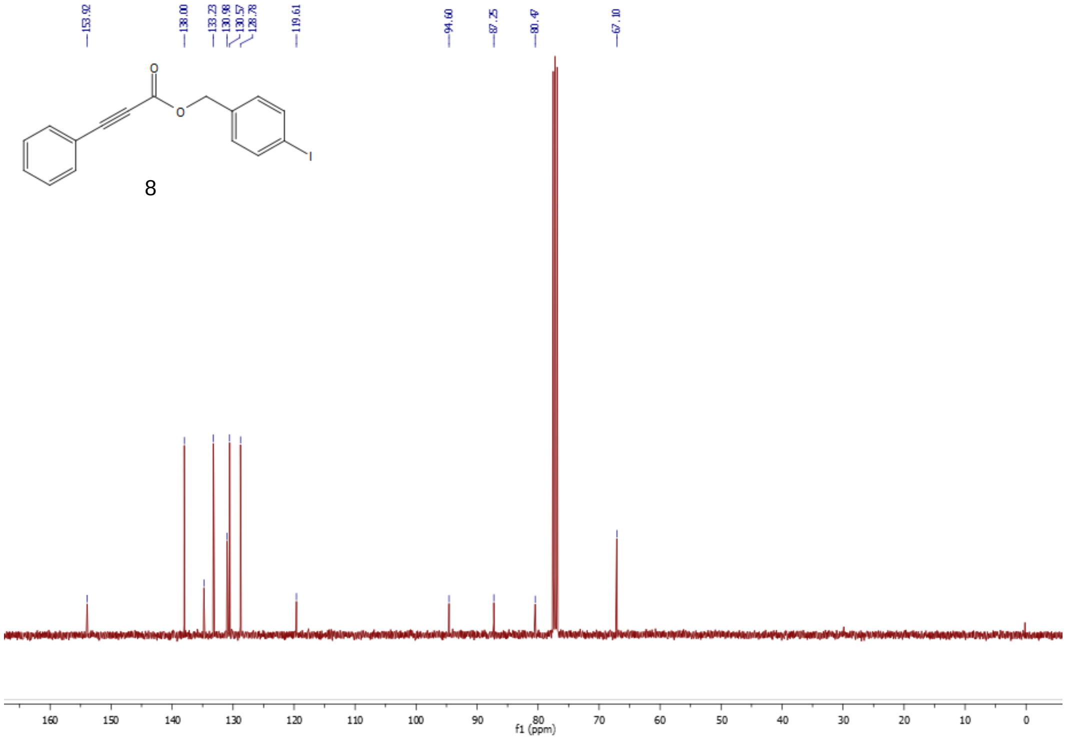
153.92
138.00
133.23
130.98
130.57
128.78
119.61
94.60
87.25
80.47
67.10
8
f1 (ppm)

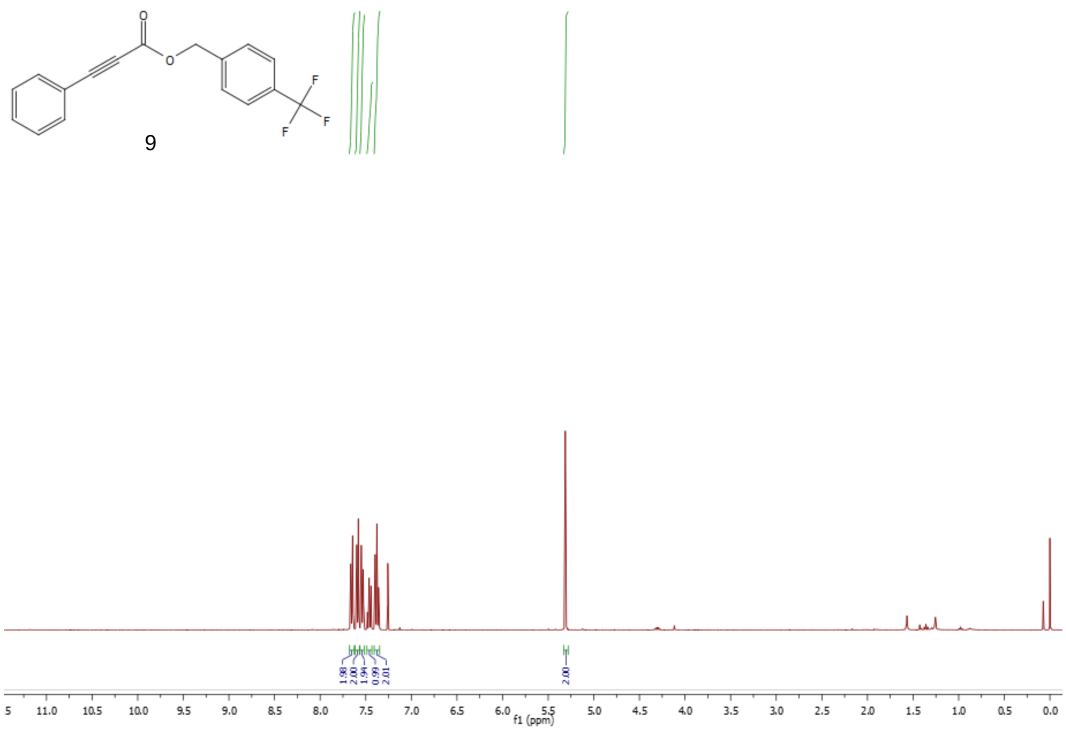
9
f1 (ppm)

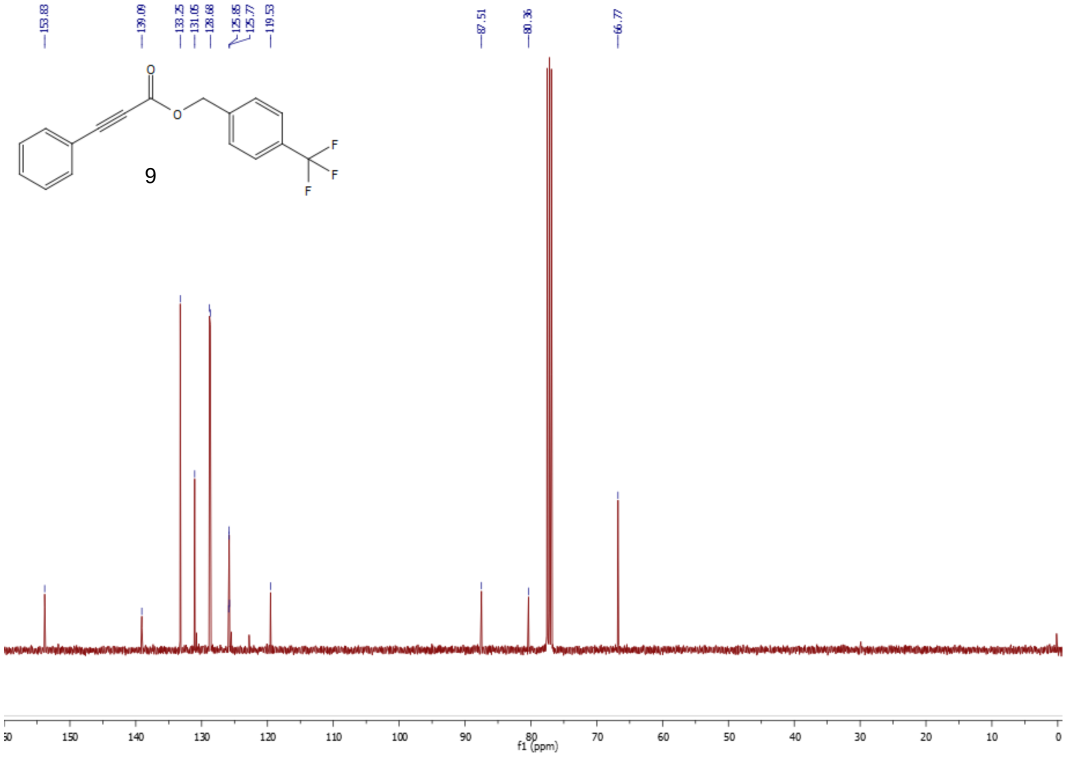
9
f1 (ppm)

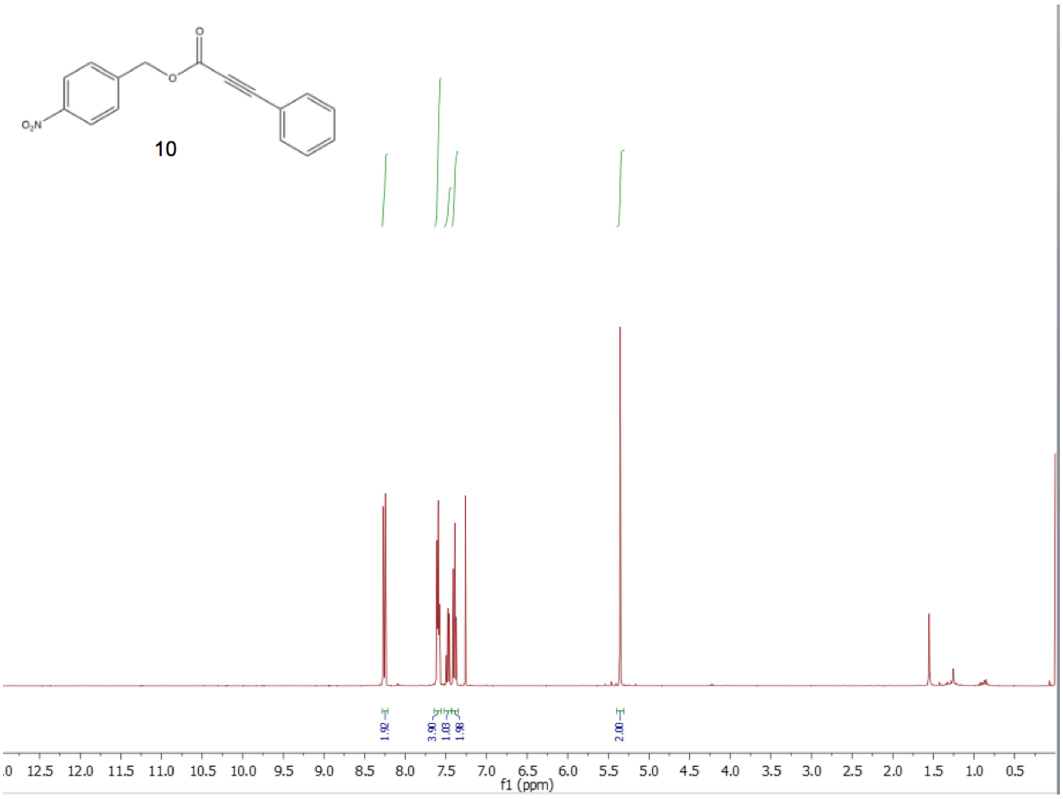
O
O_2N
10
1.92
3.90
1.03
1.98
2.00
f1 (ppm)

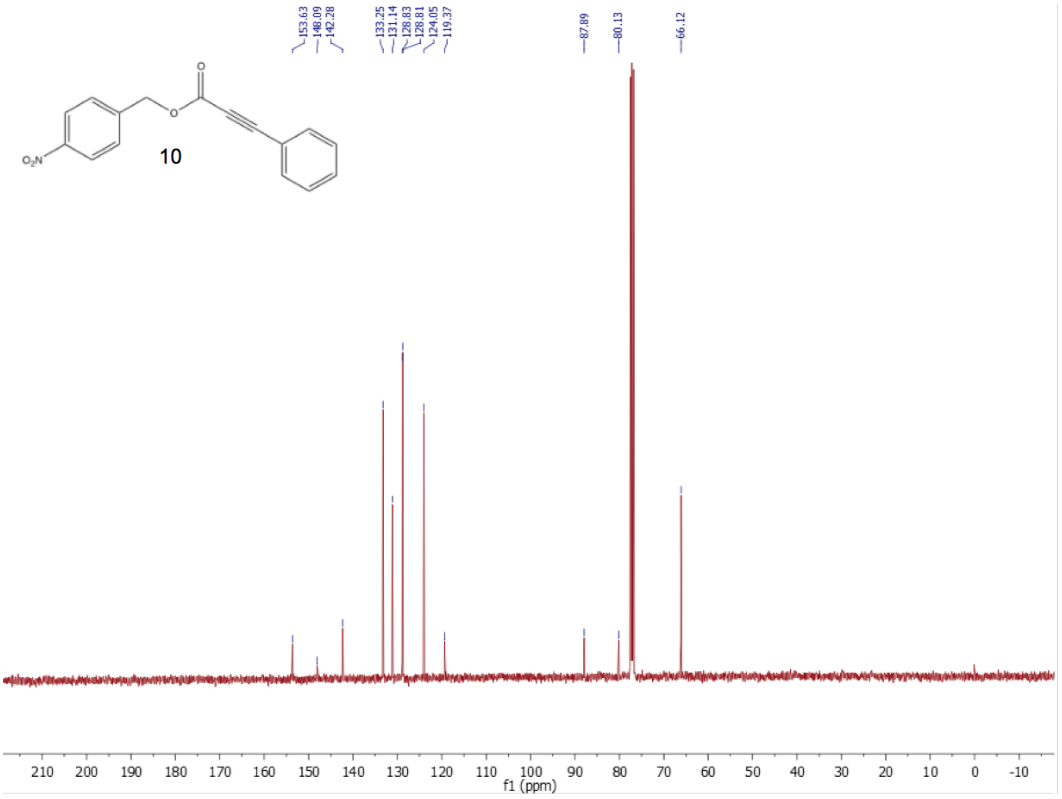
153.63
148.09
142.28
133.25
131.14
128.83
128.81
124.05
119.37
87.89
80.13
66.12
O
O_2N
10
f1 (ppm)

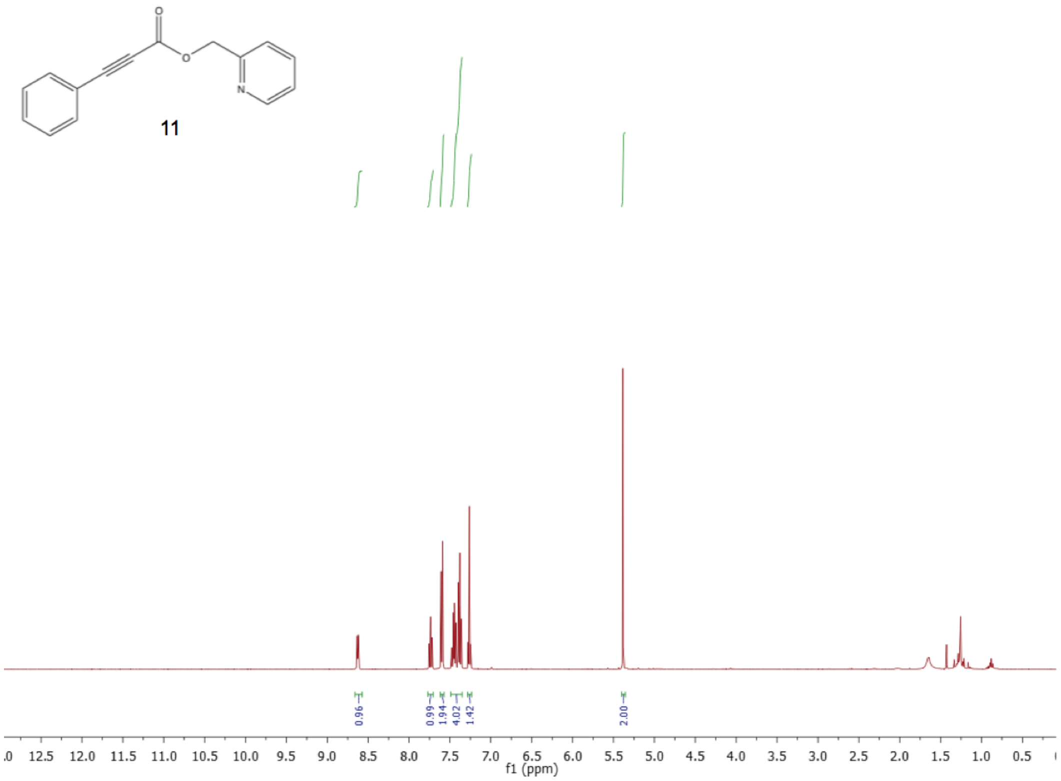
11
O
O
N
0.96
0.99
1.94
4.02
1.42
2.00
f1 (ppm)

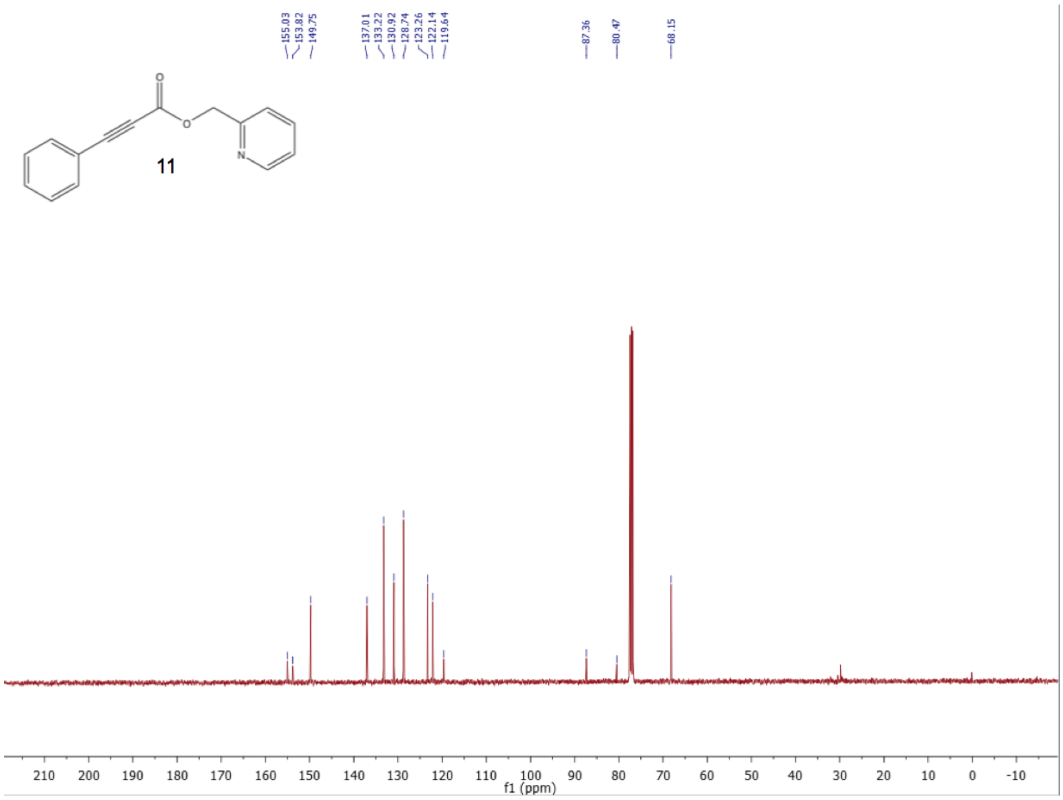
155.03
153.82
149.75
137.01
133.22
130.92
128.74
123.26
122.14
119.64
87.36
80.47
68.15
11
O
O
N
f1 (ppm)

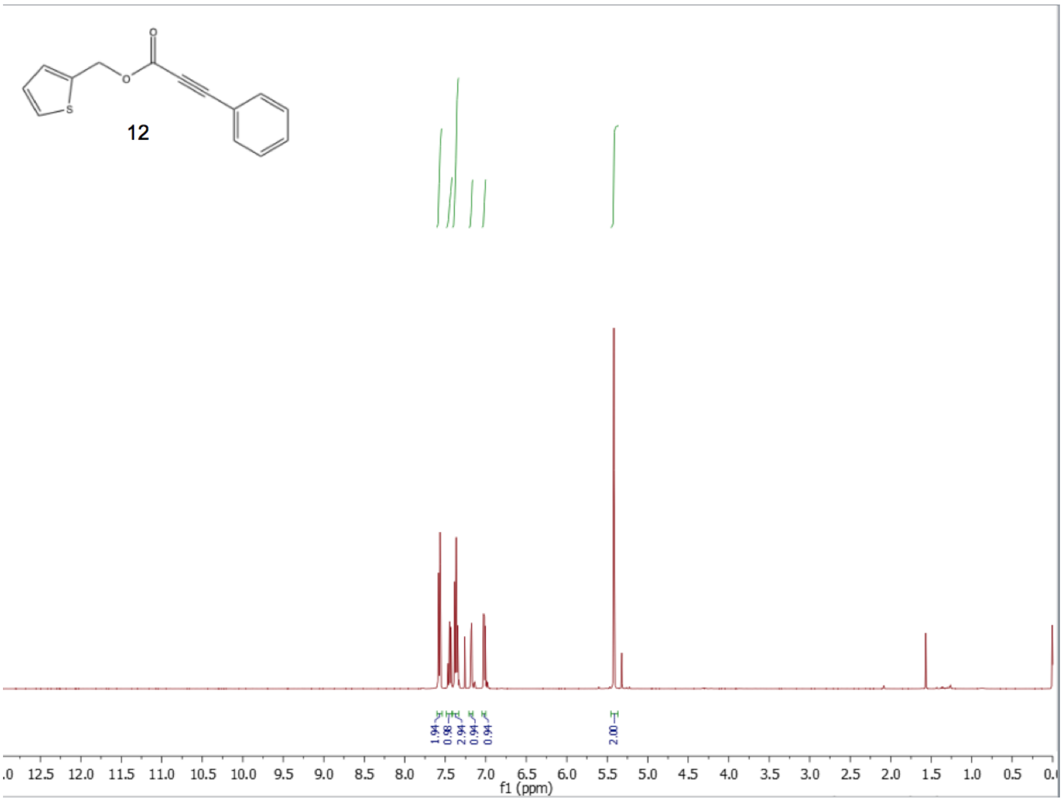
12
1.94
0.98
2.94
0.94
0.94
2.00
f1 (ppm)

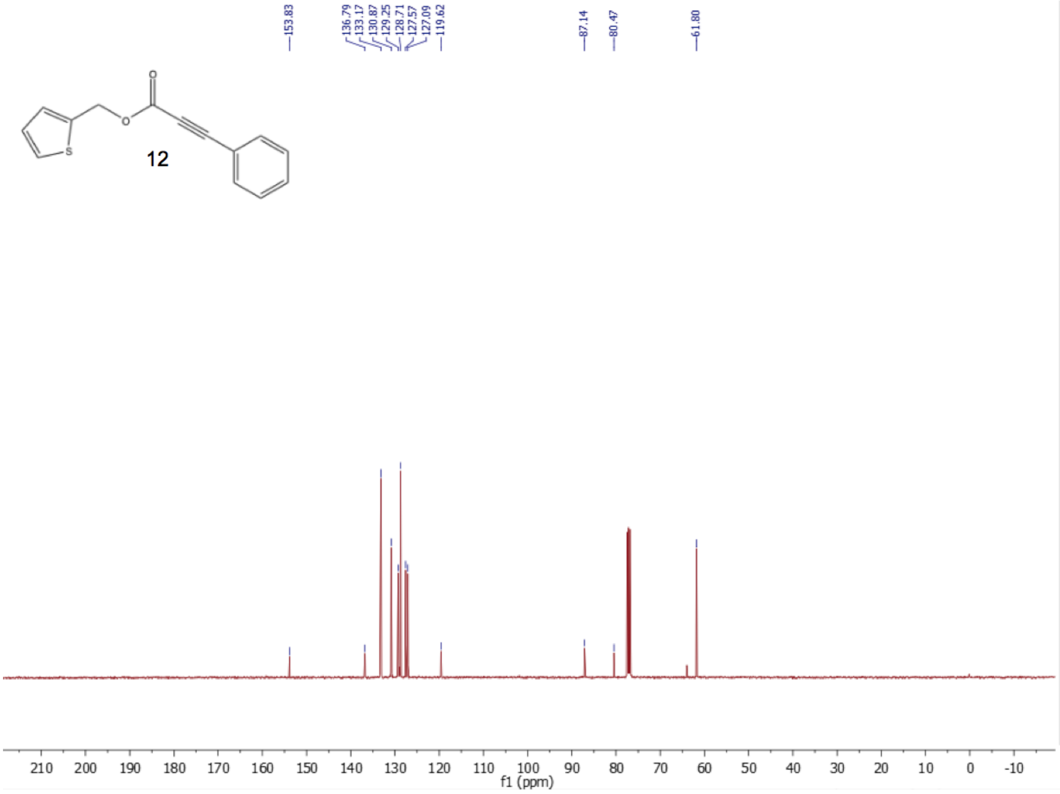
153.83
136.79
133.17
130.87
129.25
128.71
127.57
127.09
119.62
87.14
80.47
61.80
12
f1 (ppm)

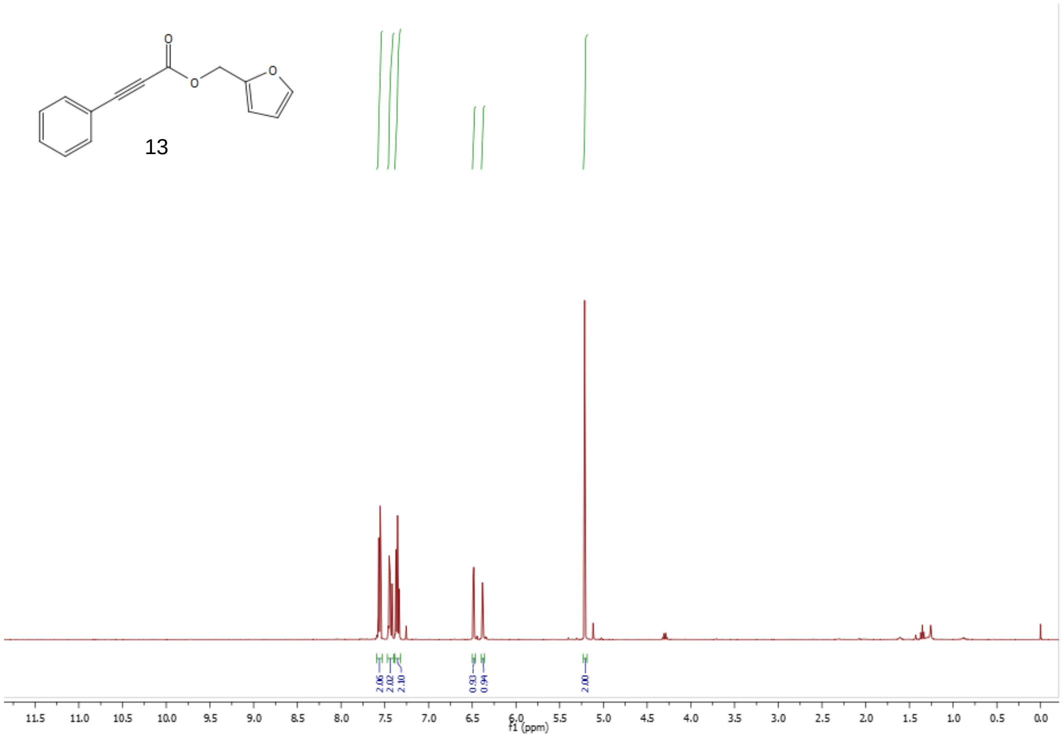
13
2.06
2.02
2.10
0.93
0.94
2.00
f1 (ppm)

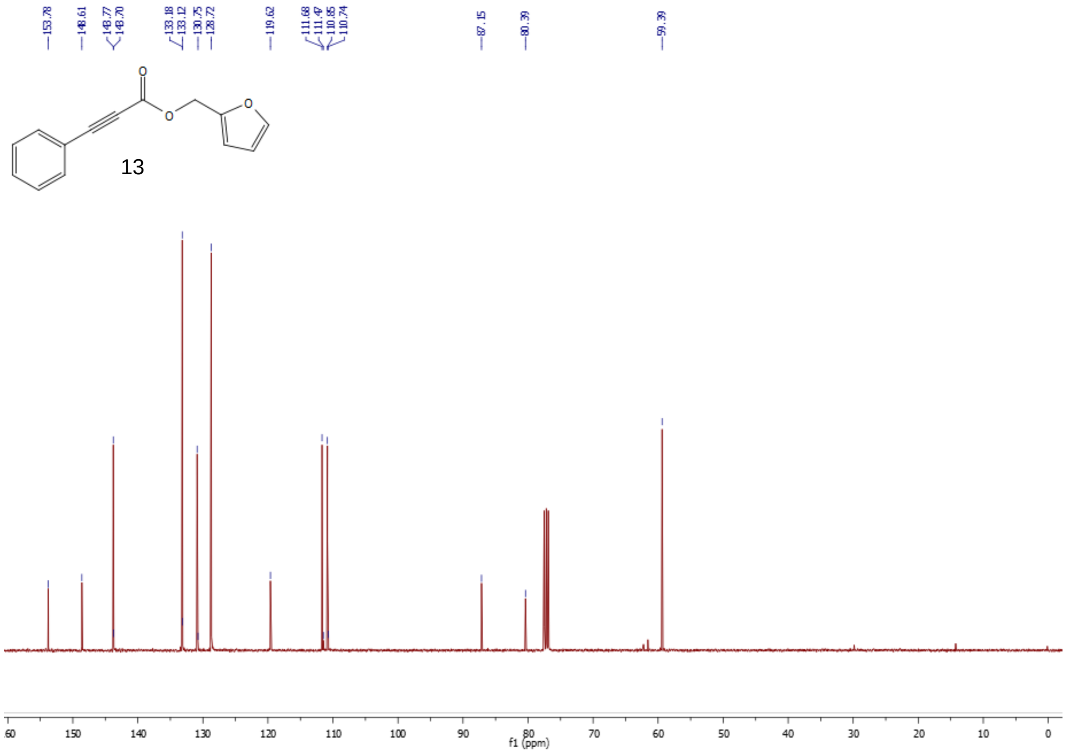
153.78
148.61
143.77
143.70
133.18
133.12
130.75
128.72
119.62
111.68
111.47
110.85
110.74
87.15
80.39
59.39
13
f1 (ppm)

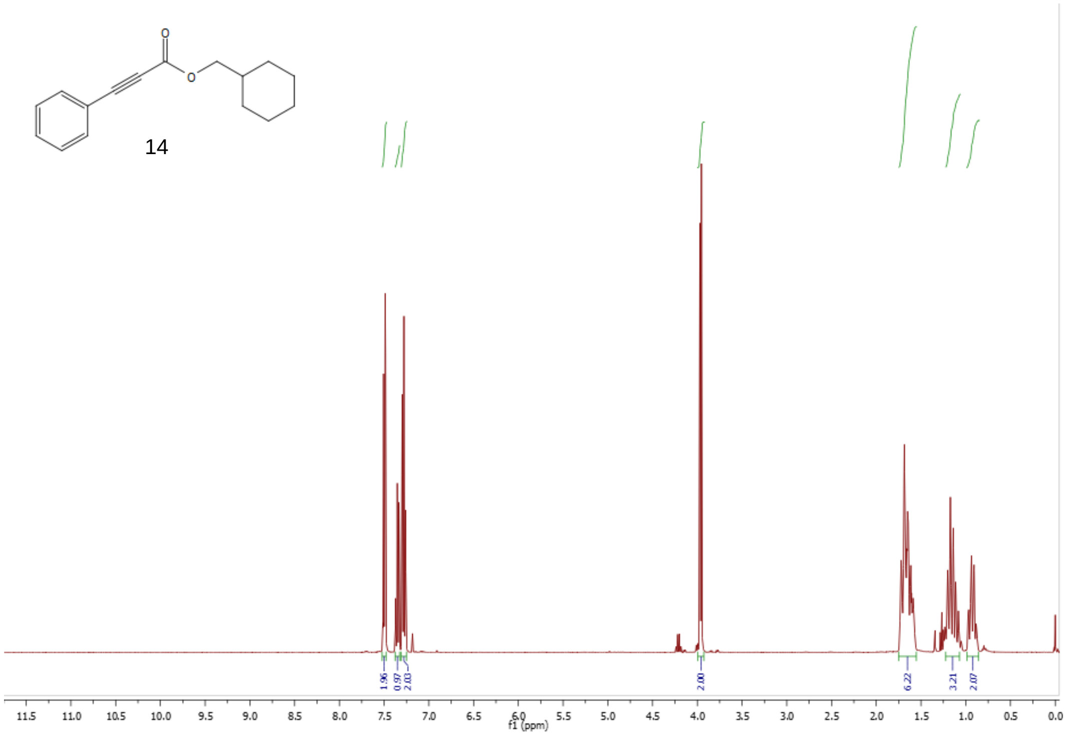
14
f1 (ppm)

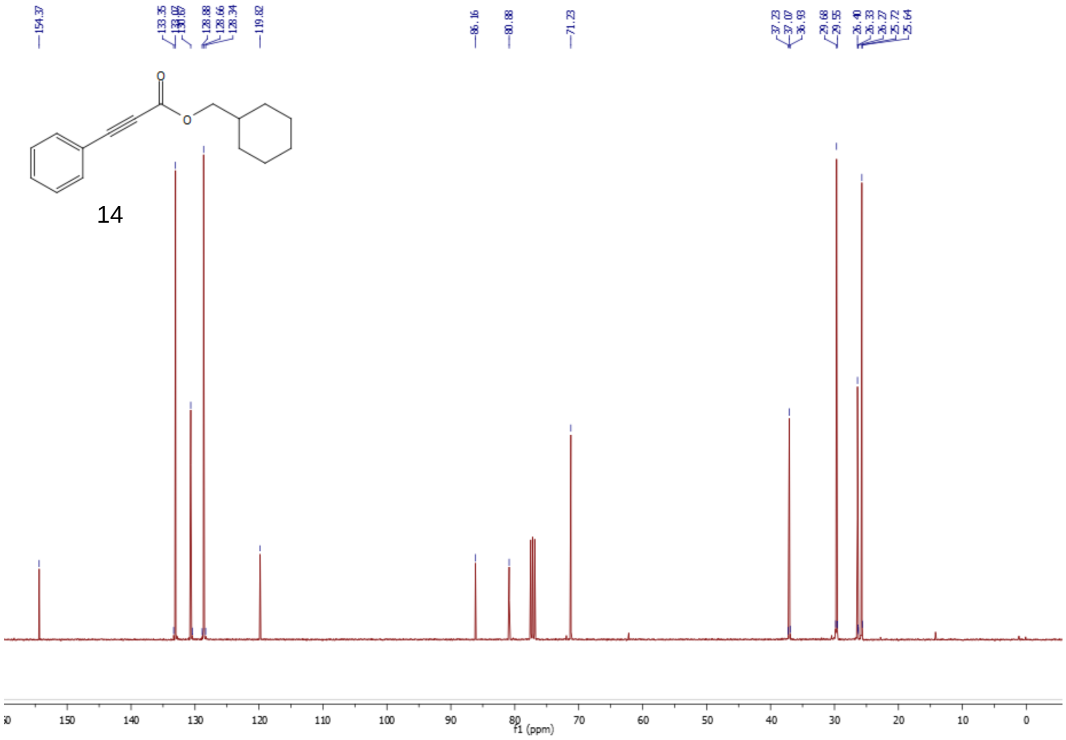
14
f1 (ppm)

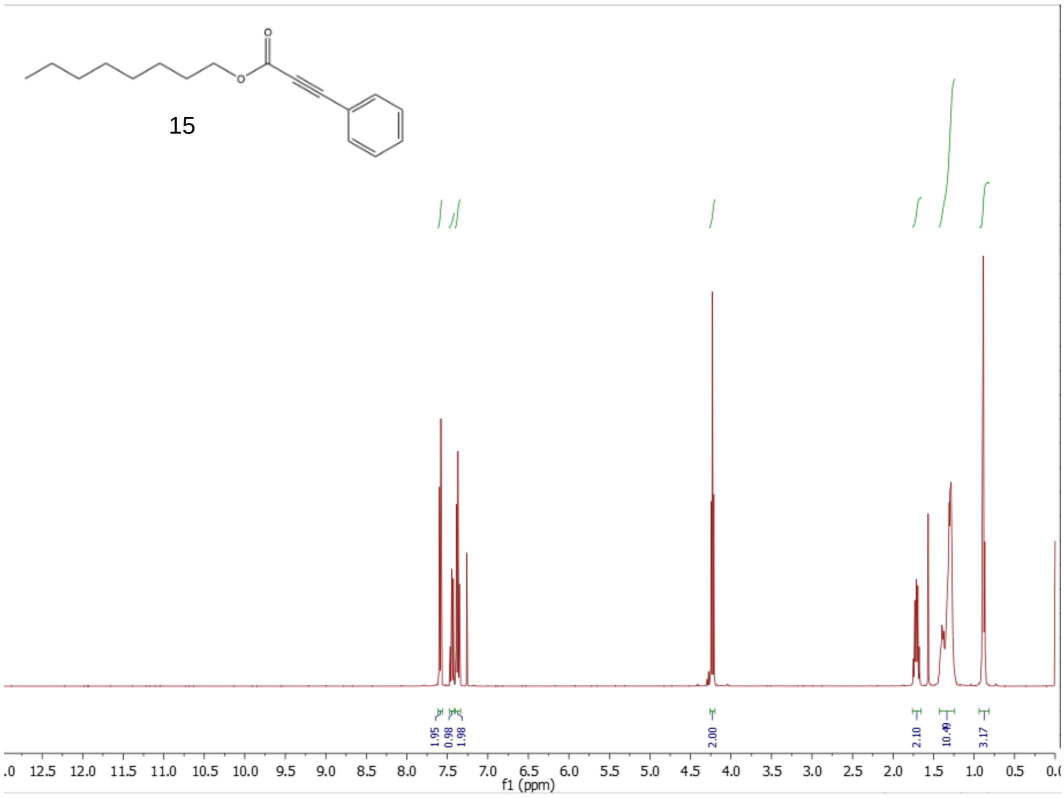
15
1.95
0.98
1.98
2.00
2.10
10.49
3.17
f1 (ppm)

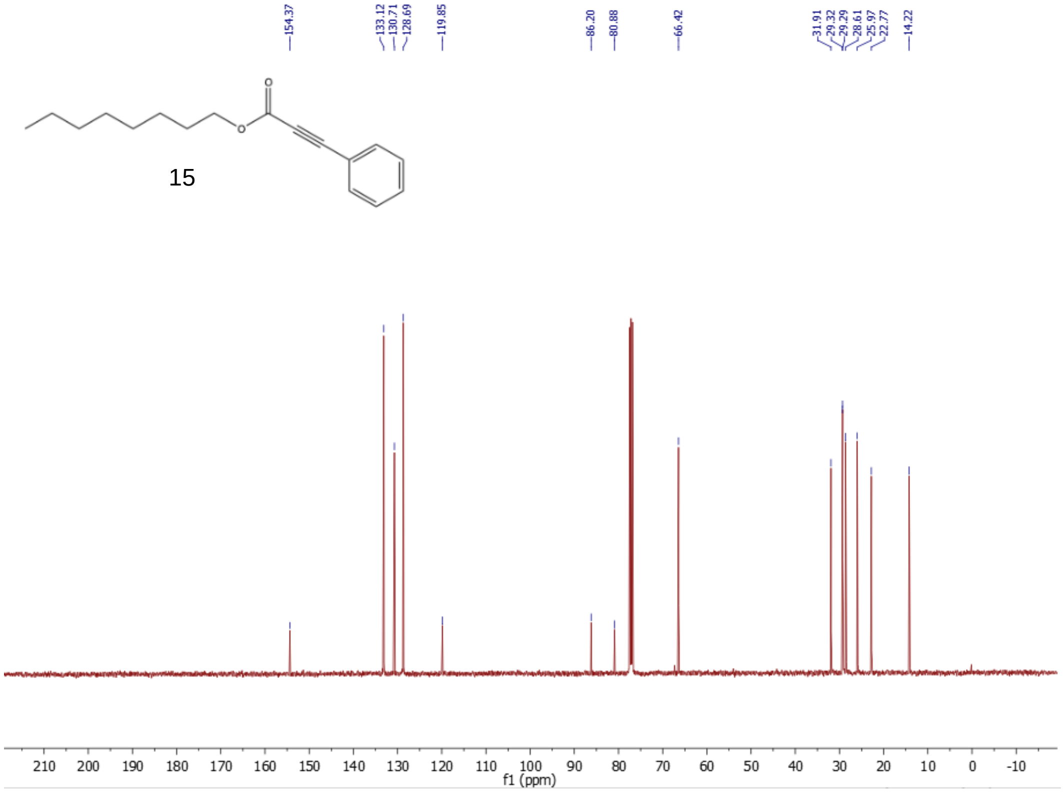
154.37
133.12
130.71
128.69
119.85
86.20
80.88
66.42
31.91
29.32
29.29
28.61
25.97
22.77
14.22
15
f1 (ppm)

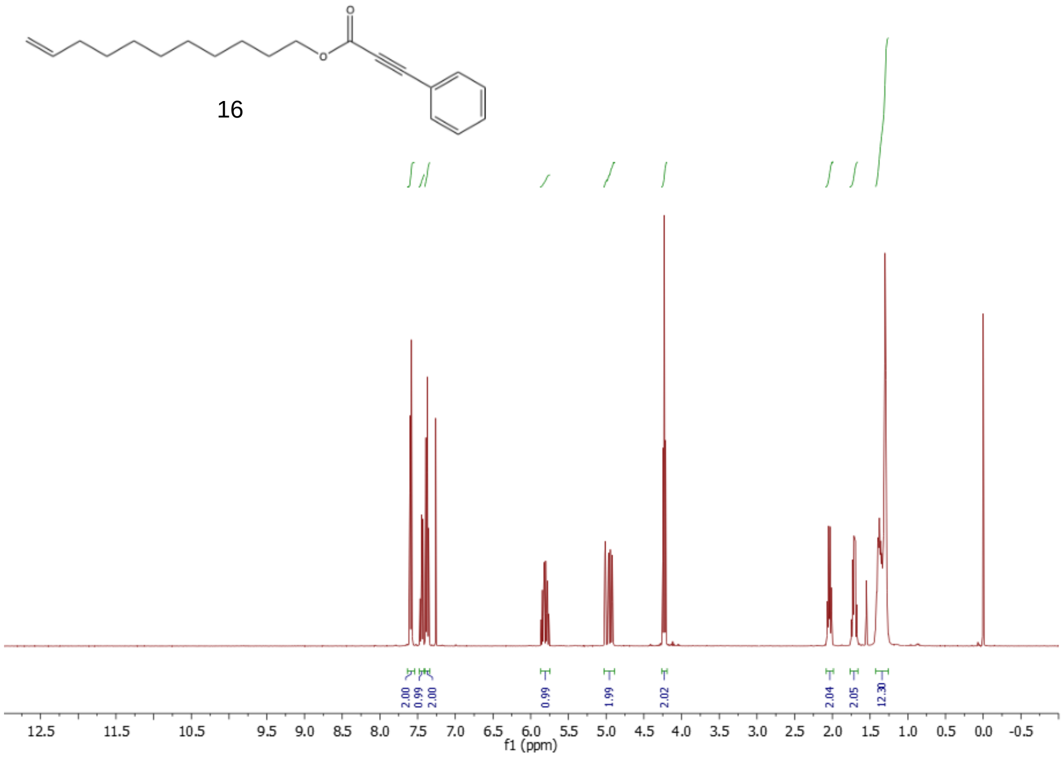
16
2.00 0.99 2.00
0.99
1.99
2.02
2.04
2.05
12.30
f1 (ppm)

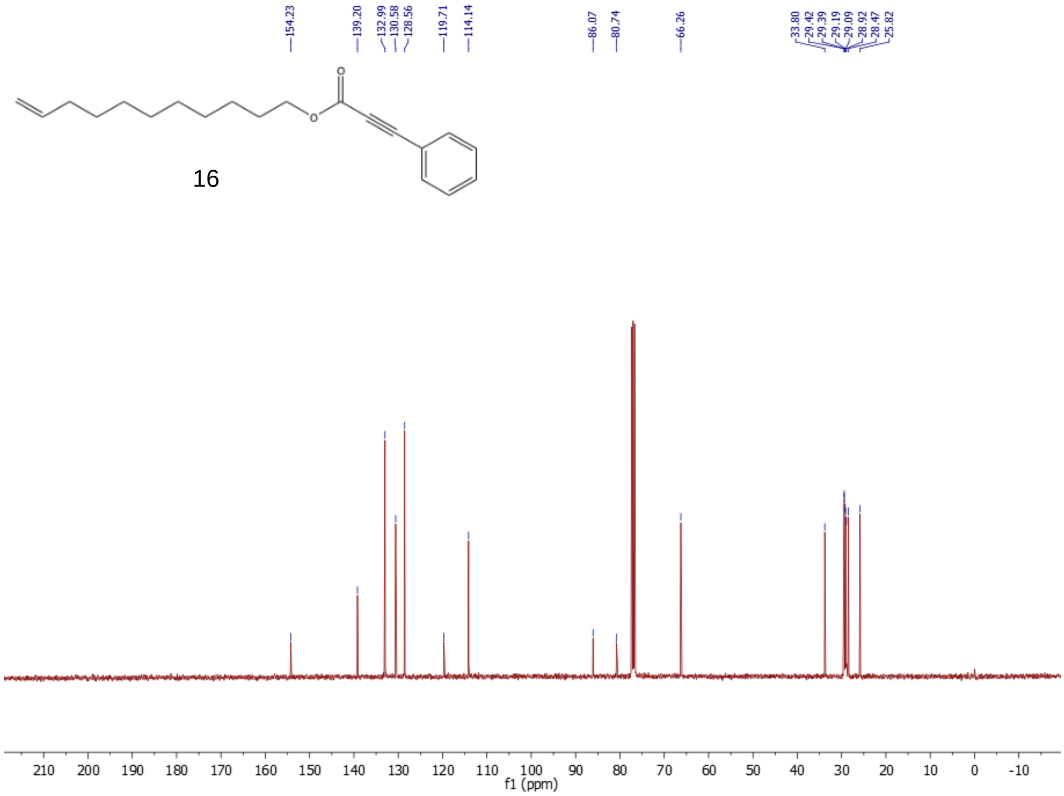
154.23
139.20
132.99
130.58
128.56
119.71
114.14
86.07
80.74
66.26
33.80
29.42
29.39
29.19
29.09
28.92
28.47
25.82
16
f1 (ppm)

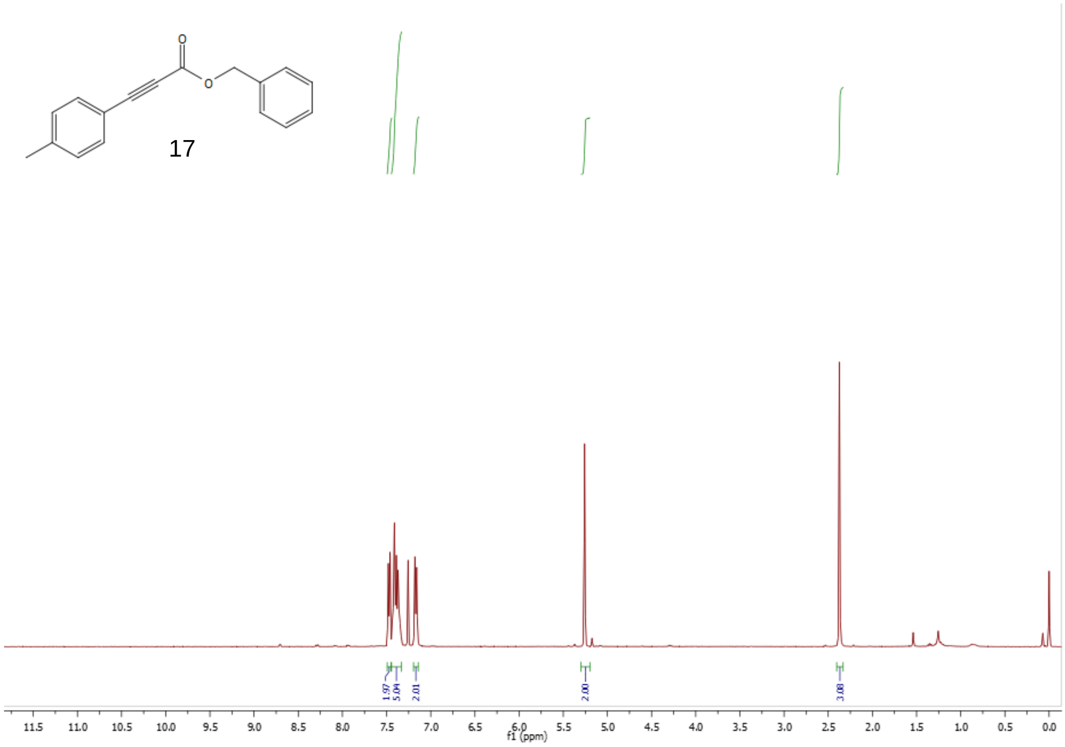

O
O
17
1.97
5.04
2.01
2.00
3.08
f1 (ppm)

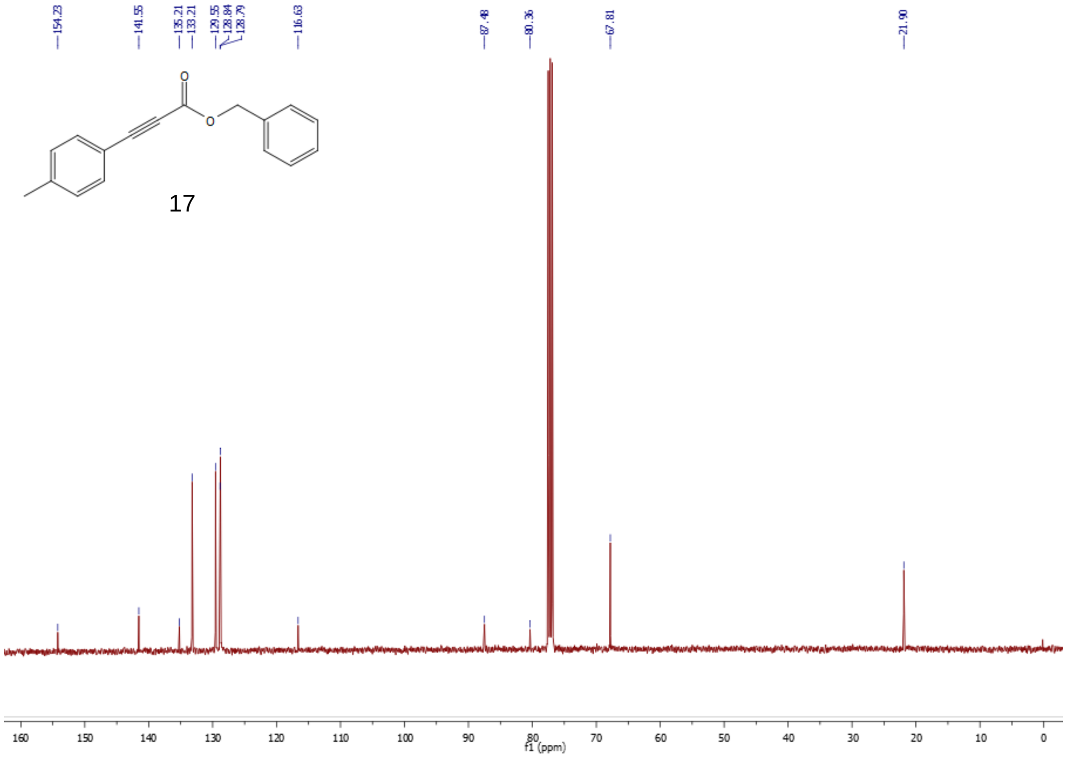

154.23
141.55
135.21
133.21
129.55
128.84
128.79
116.63
87.48
80.36
67.81
21.90
O
O
17
f1 (ppm)

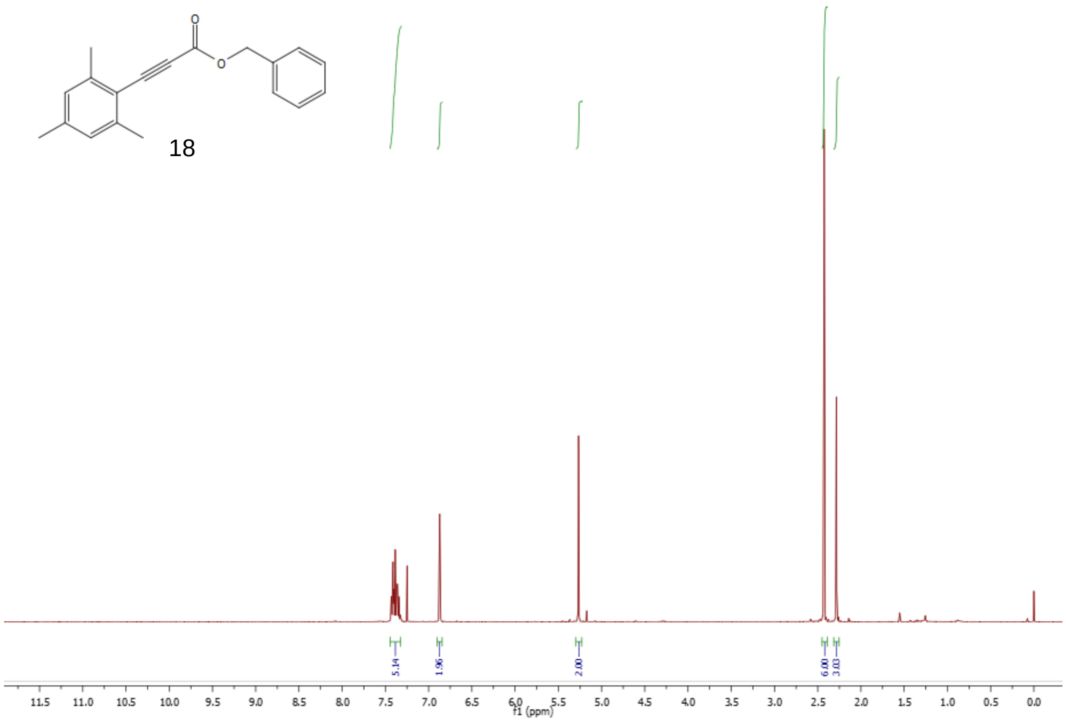
18
5.14
1.96
2.00
6.00
3.03
f1 (ppm)

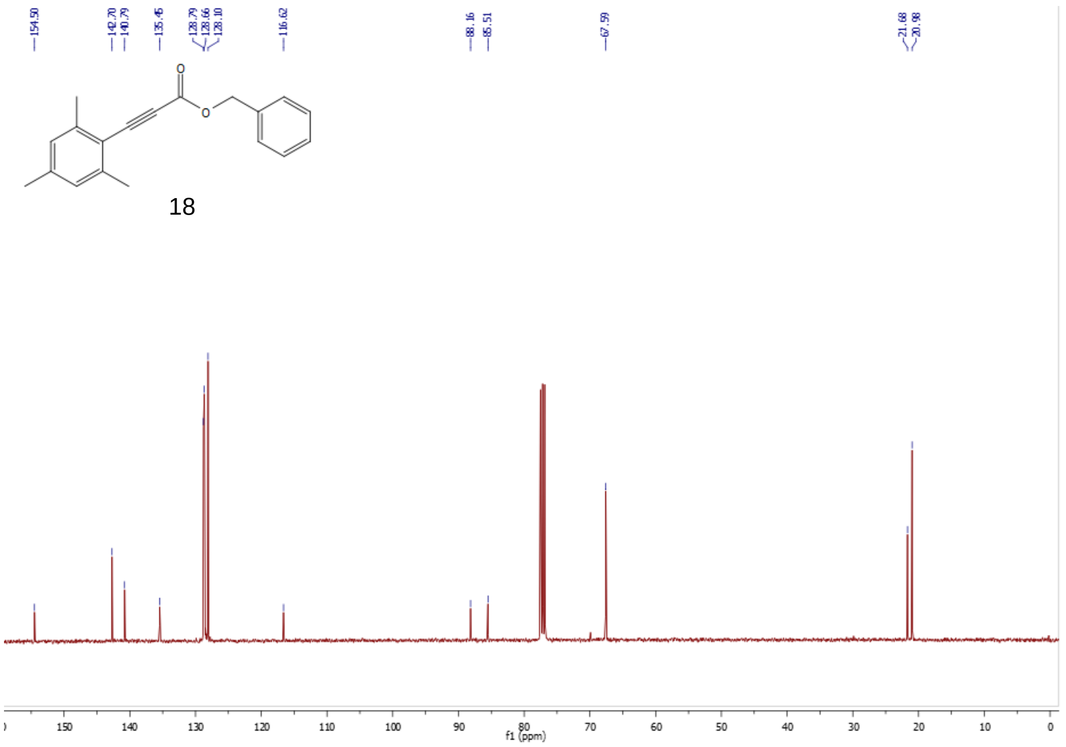
154.50
142.70
140.79
135.45
128.79
128.66
128.10
116.62
88.16
85.51
67.59
21.68
20.98
18
f1 (ppm)

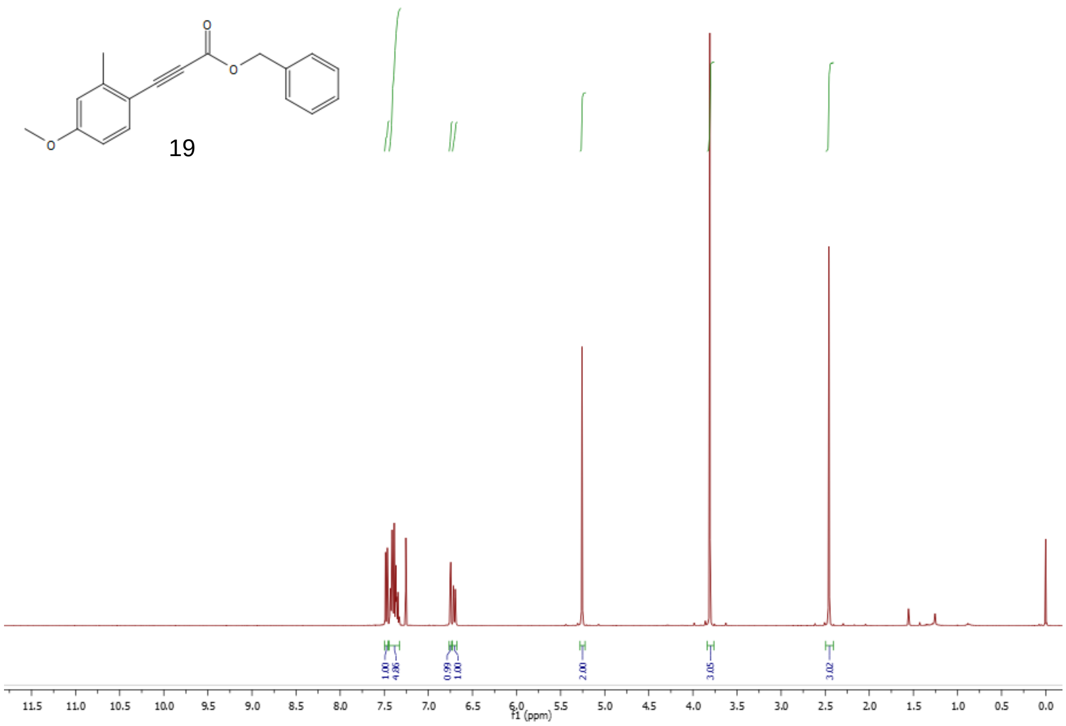
19
1.00
4.86
0.99
1.00
2.00
3.05
3.02
f1 (ppm)

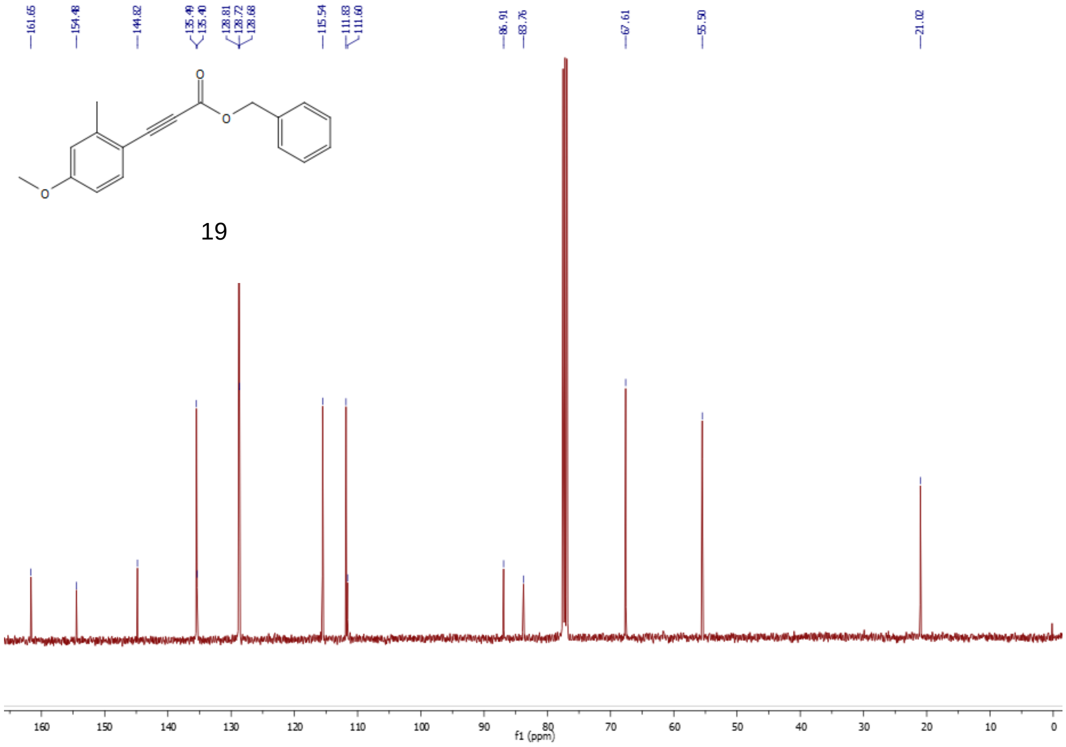
161.65
154.48
144.82
135.49
135.40
128.81
128.72
128.68
115.54
111.83
111.60
86.91
83.76
67.61
55.50
21.02
19
f1 (ppm)

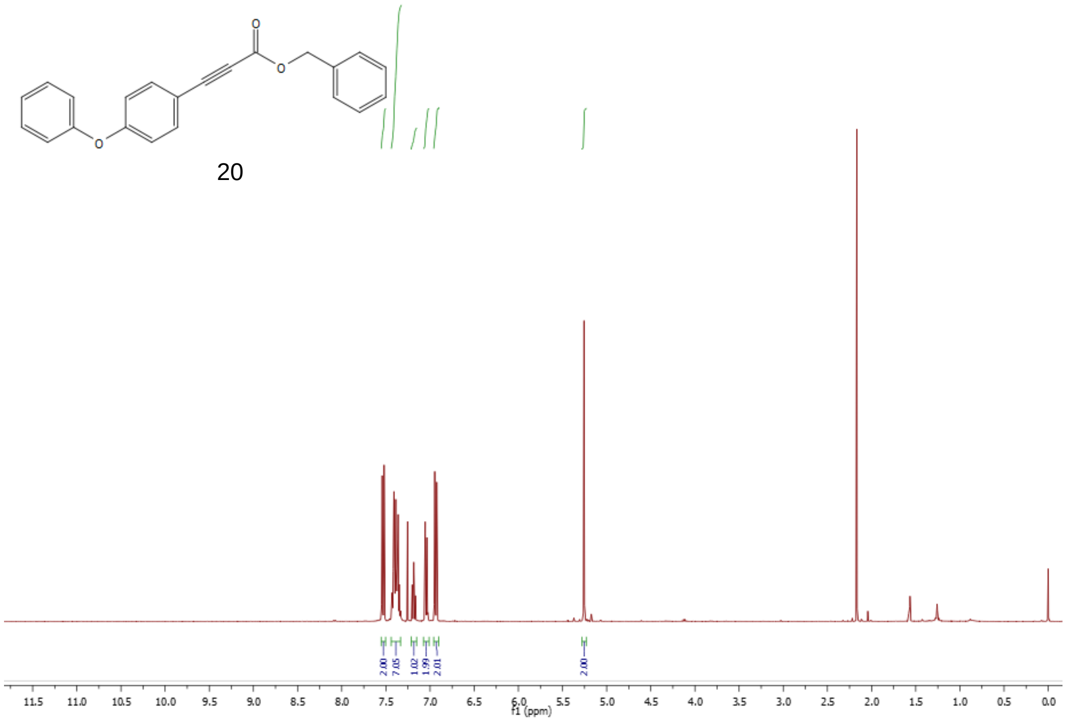
20
2.00
7.05
1.02
1.99
2.01
2.00
f1 (ppm)

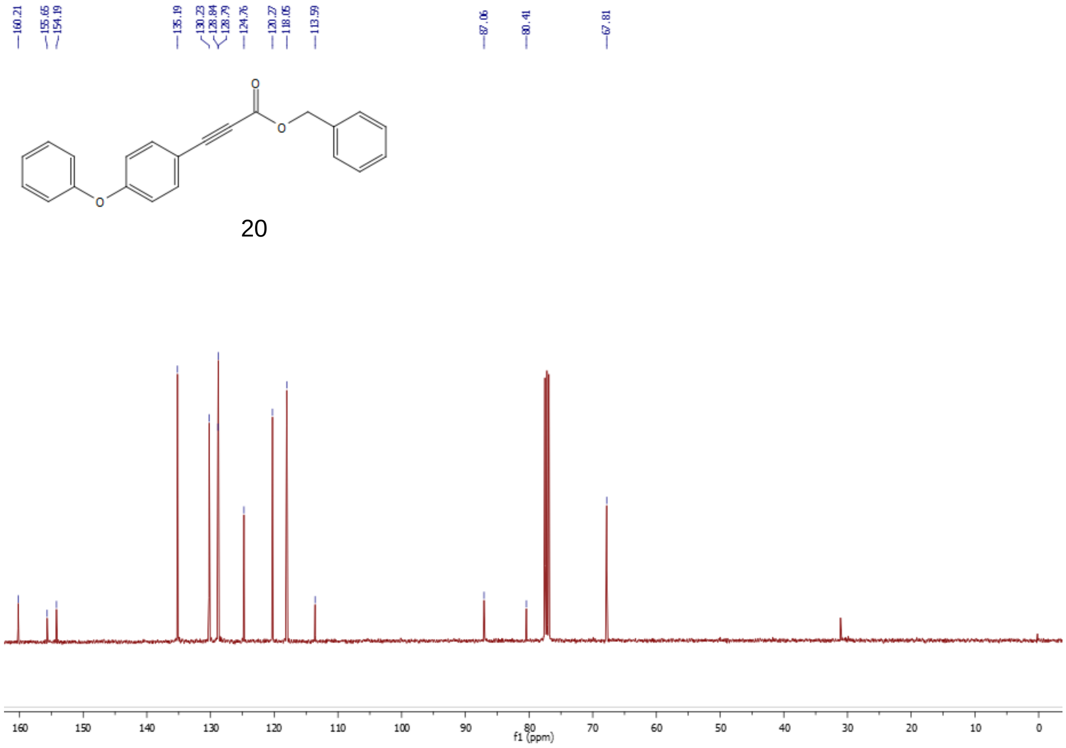
160.21
155.65
154.19
135.19
130.23
128.84
128.79
124.76
120.27
118.05
113.59
87.06
80.41
67.81
20
f1 (ppm)

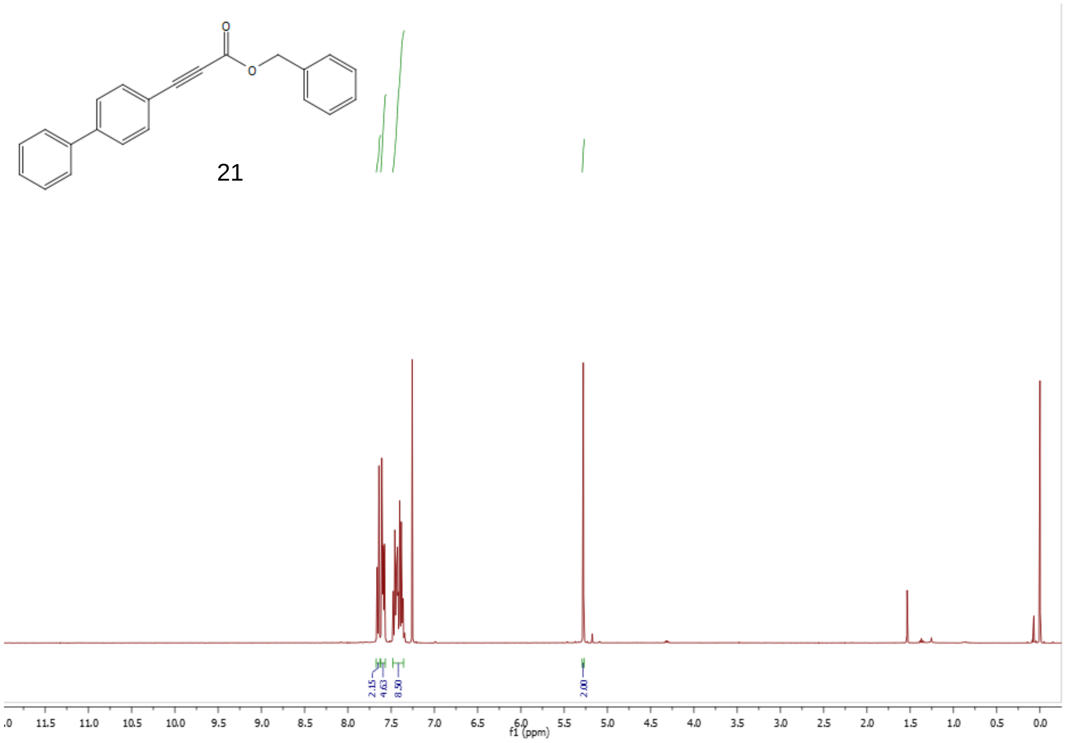
O
O
21
2.15
4.63
8.50
2.00
f1 (ppm)

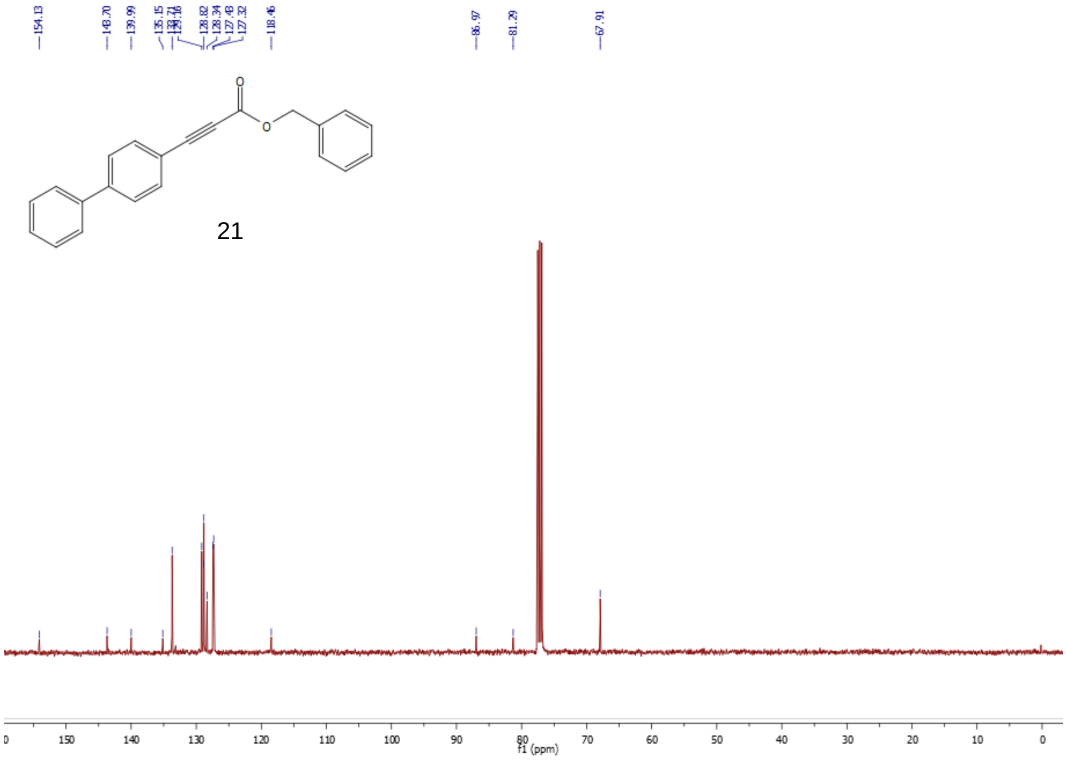
154.13
143.70
139.99
135.15
128.82
128.34
127.43
127.32
118.46
86.97
81.29
67.91
O
O
21
f1 (ppm)

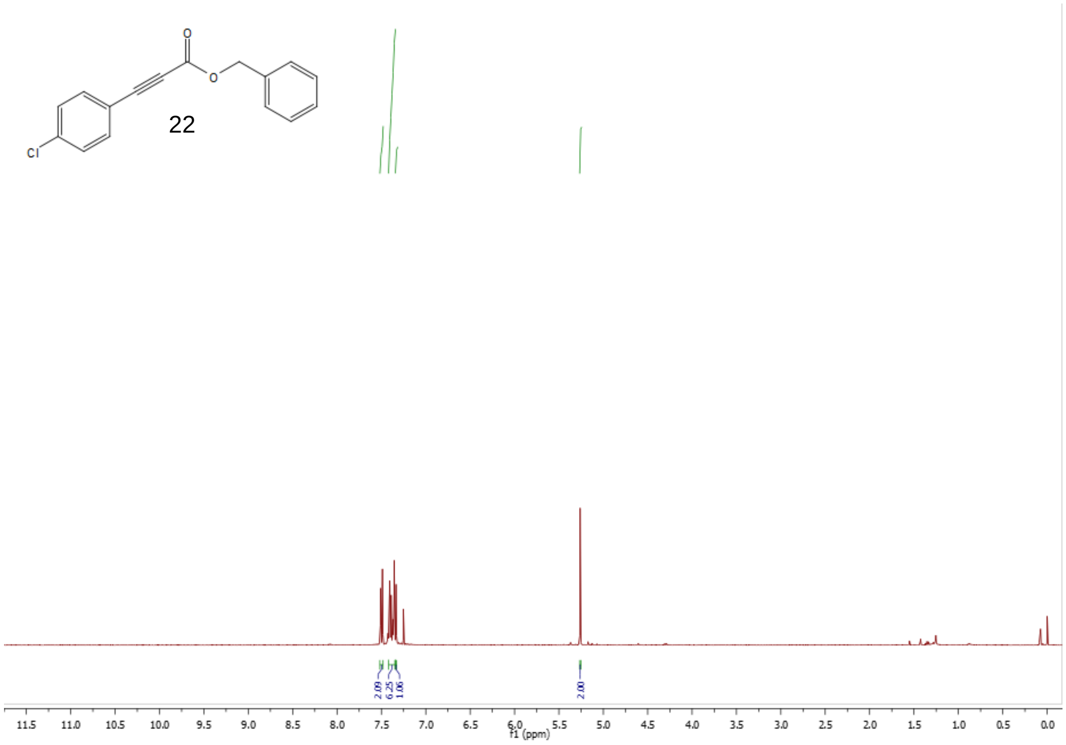
22
Cl
O
2.09
6.25
1.06
2.00
f1 (ppm)

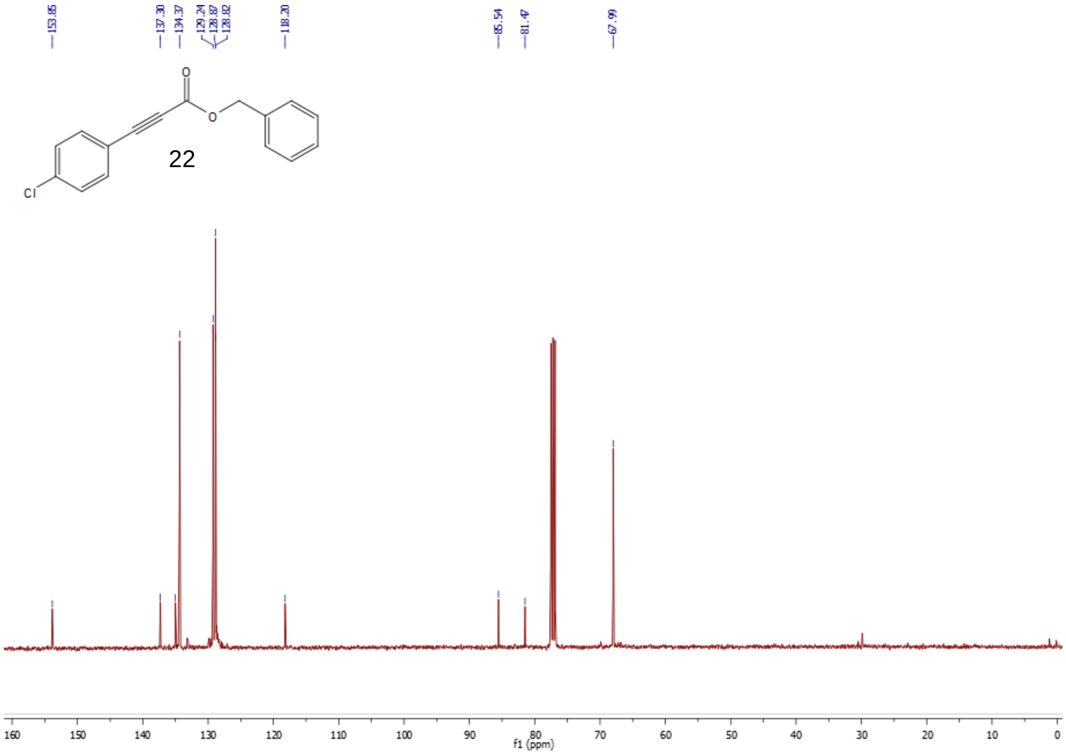
153.85
137.30
134.37
129.24
128.87
128.82
118.20
85.54
81.47
67.99
22
Cl
O
f1 (ppm)

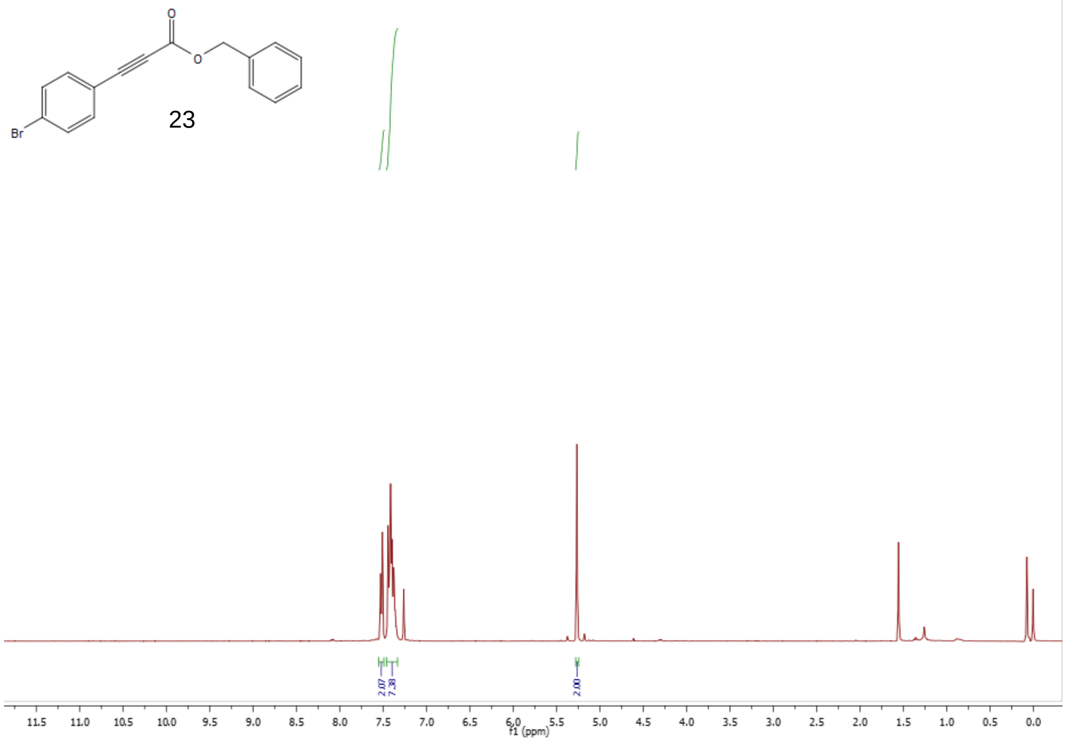
23
2.07
7.38
2.00
f1 (ppm)

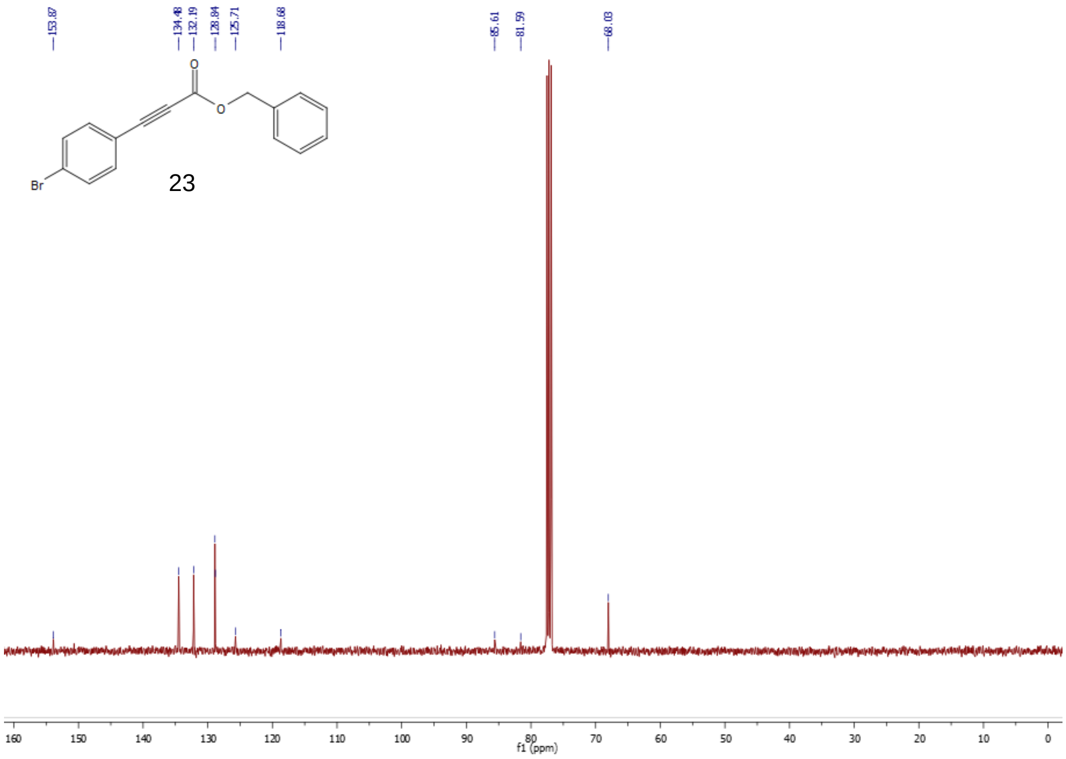
153.87
134.48
132.19
128.84
125.71
118.68
85.61
81.59
68.03
23
f1 (ppm)

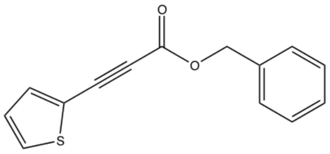

24

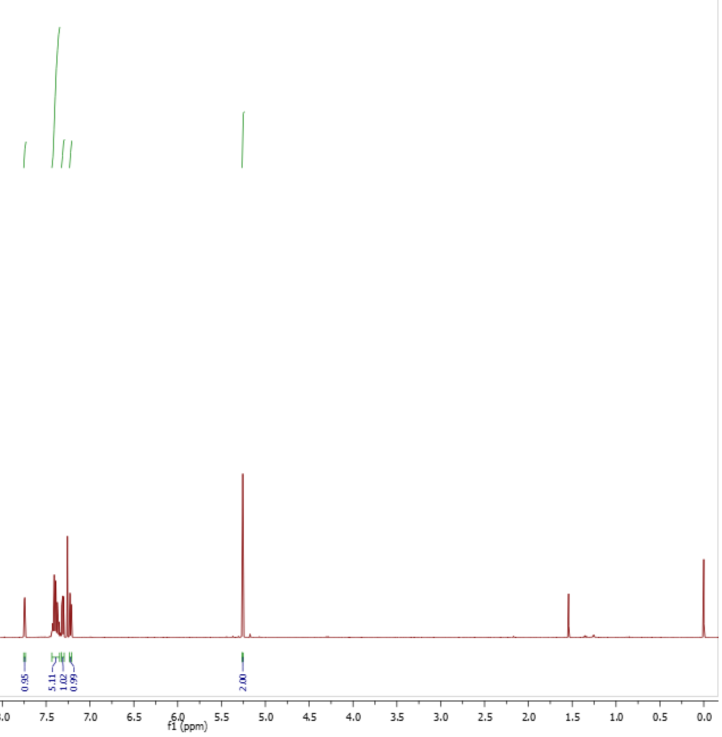
0.95
5.11
1.02
0.99
2.00
11.5 11.0 10.5 10.0 9.5 9.0 8.5 8.0 7.5 7.0 6.5 6.0 5.5 5.0 4.5 4.0 3.5 3.0 2.5 2.0 1.5 1.0 0.5 0.0
f1 (ppm)

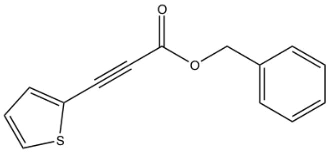

24

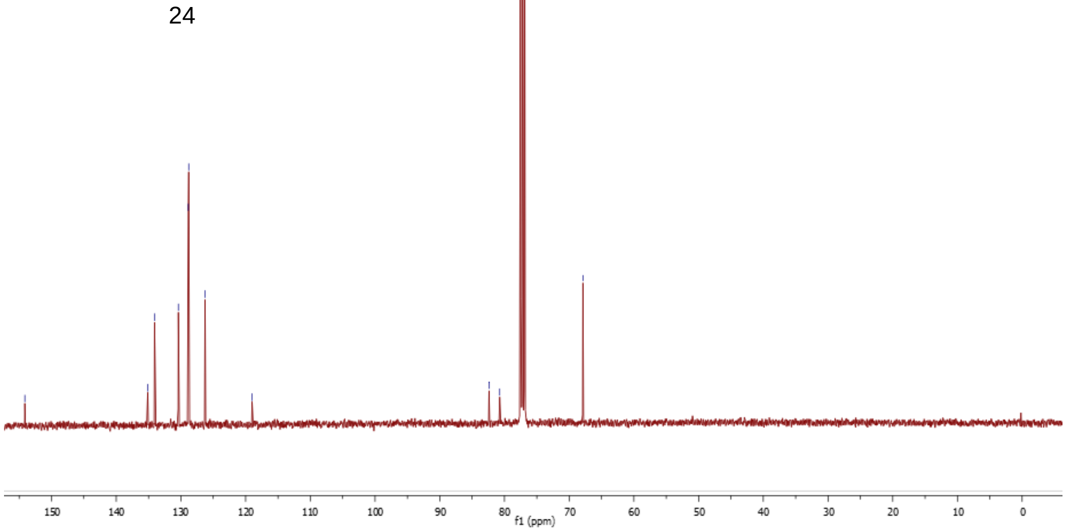
154.12
135.12
134.08
128.80
126.27
119.01
82.39
80.74
67.88
150 140 130 120 110 100 90 80 70 60 50 40 30 20 10 0
f1 (ppm)

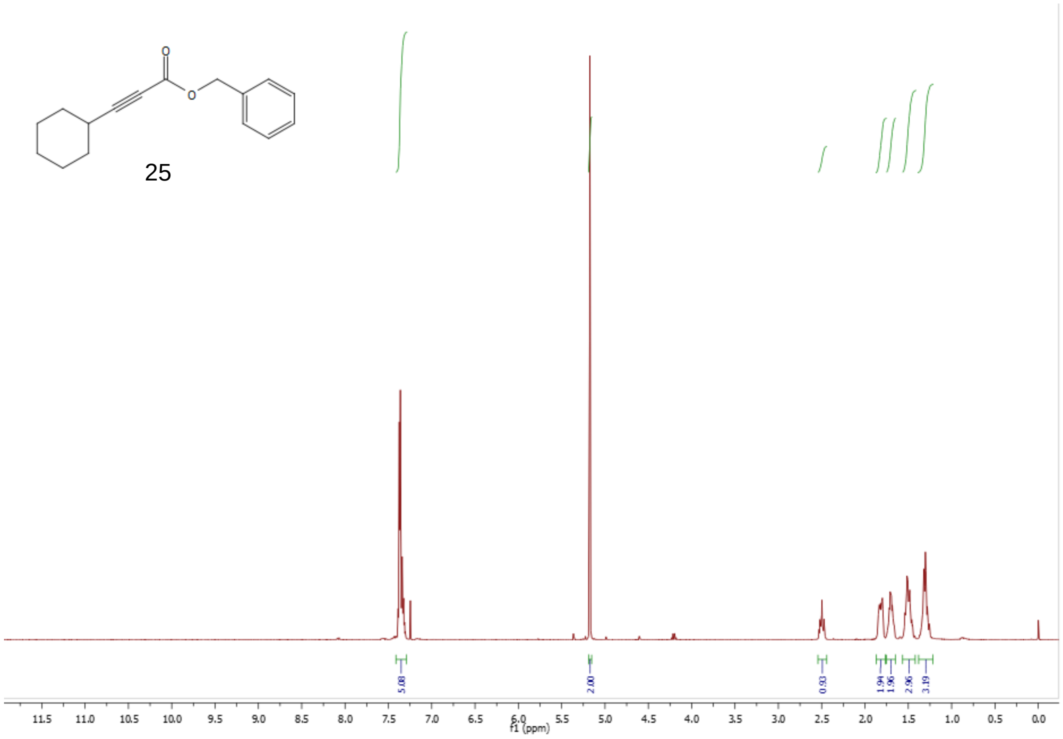
25
5.08
2.00
0.93
1.94
1.96
2.96
3.19
f1 (ppm)

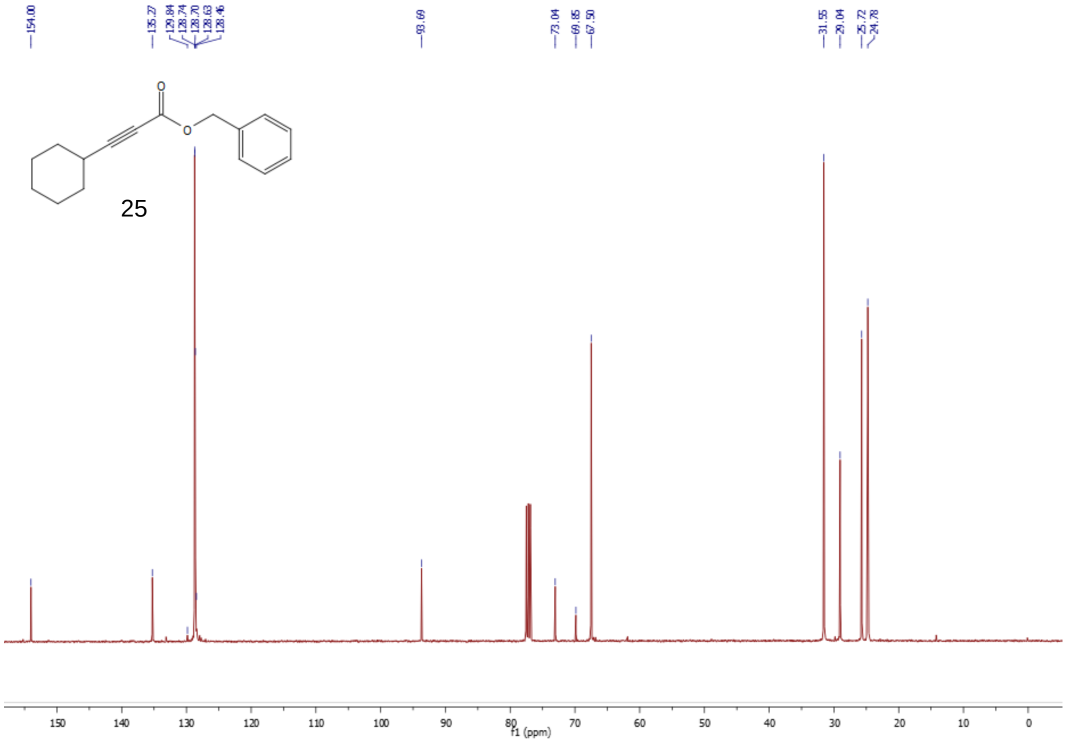
154.00
135.27
129.84
128.74
128.70
128.63
128.46
93.69
73.04
69.85
67.50
31.55
29.04
25.72
24.78
25
f1 (ppm)

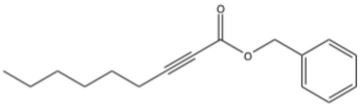

26

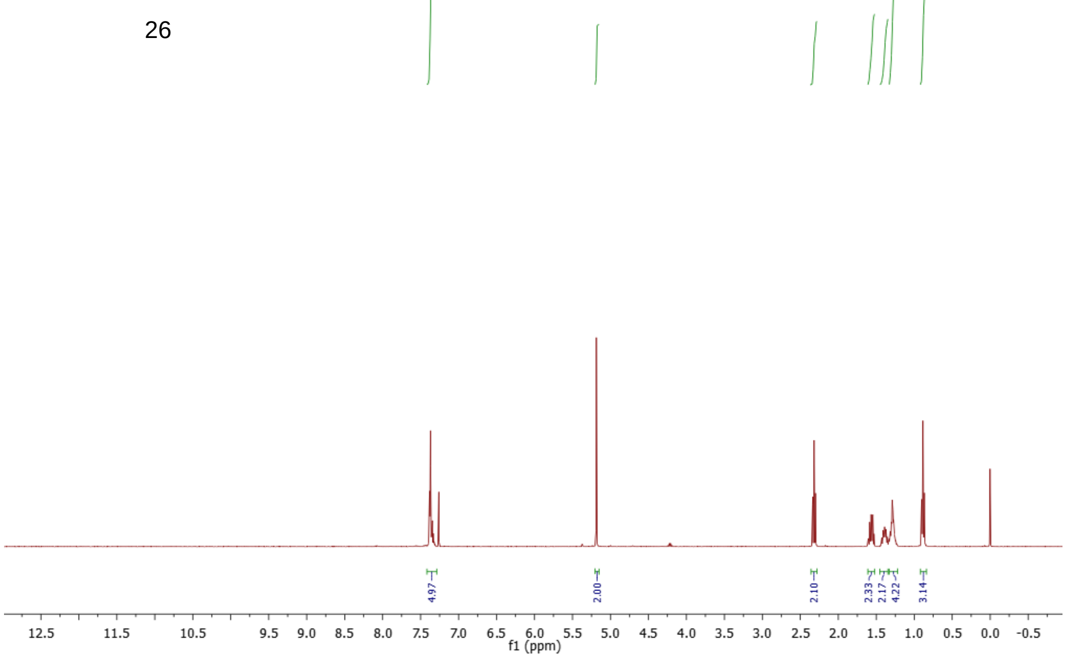
4.97
2.00
2.10
2.33
2.17
4.22
3.14
12.5 11.5 10.5 9.5 9.0 8.5 8.0 7.5 7.0 6.5 6.0 5.5 5.0 4.5 4.0 3.5 3.0 2.5 2.0 1.5 1.0 0.5 0.0 -0.5
f1 (ppm)

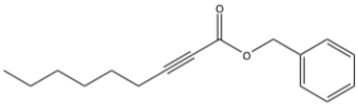

26

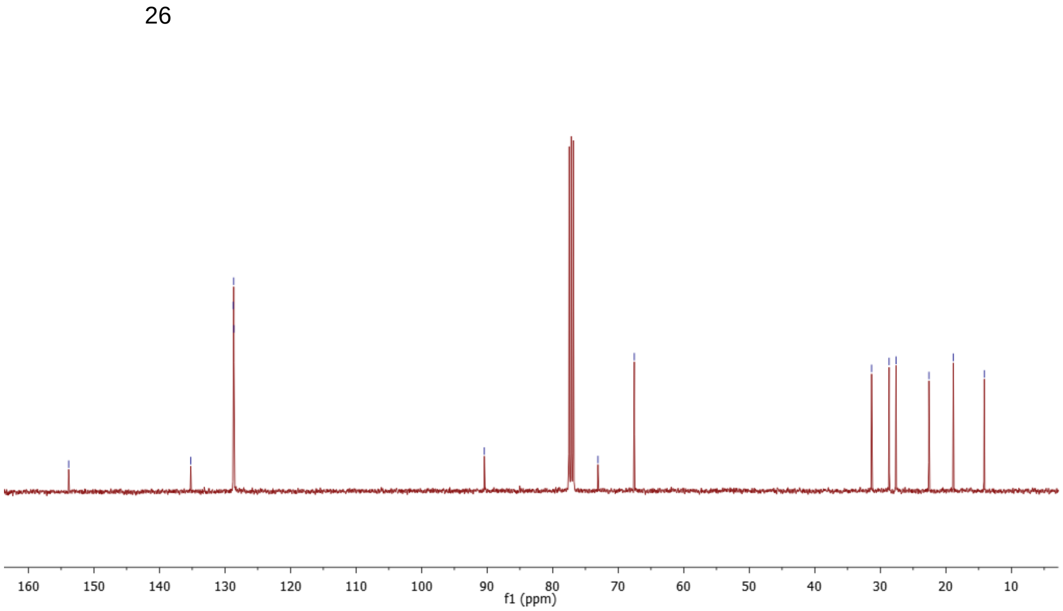
153.85
135.22
128.75
128.67
128.65
90.42
73.09
67.54
31.33
28.66
27.61
22.58
18.86
14.14
160 150 140 130 120 110 100 90 80 70 60 50 40 30 20 10
f1 (ppm)

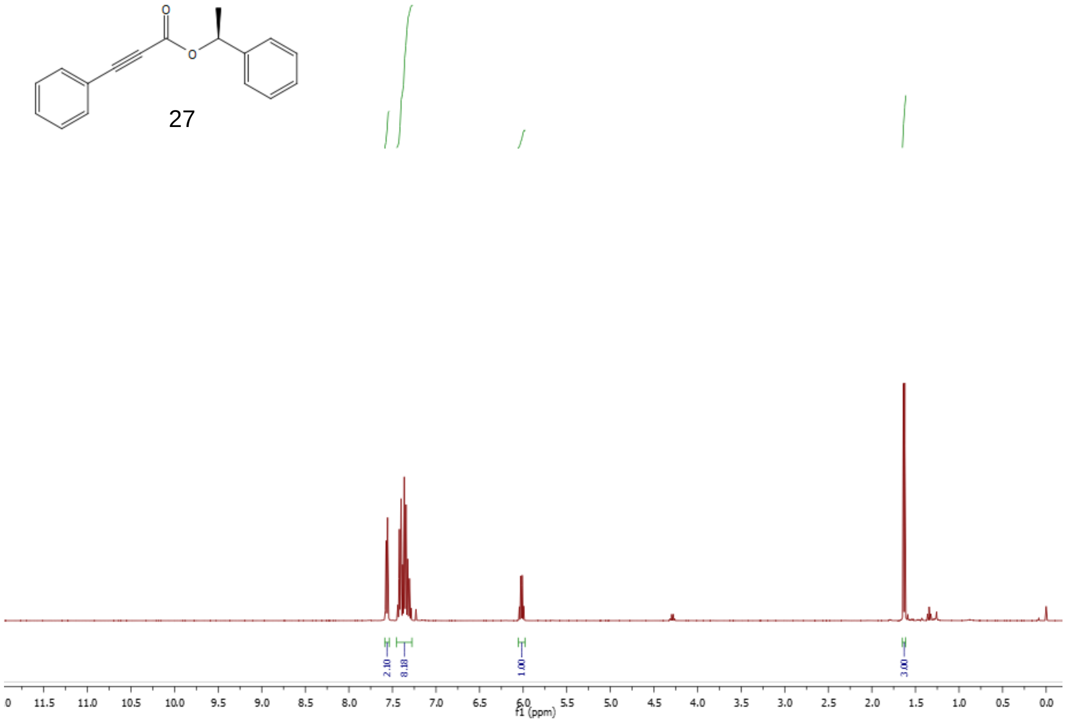
27
2.10
8.18
1.00
3.00
f1 (ppm)

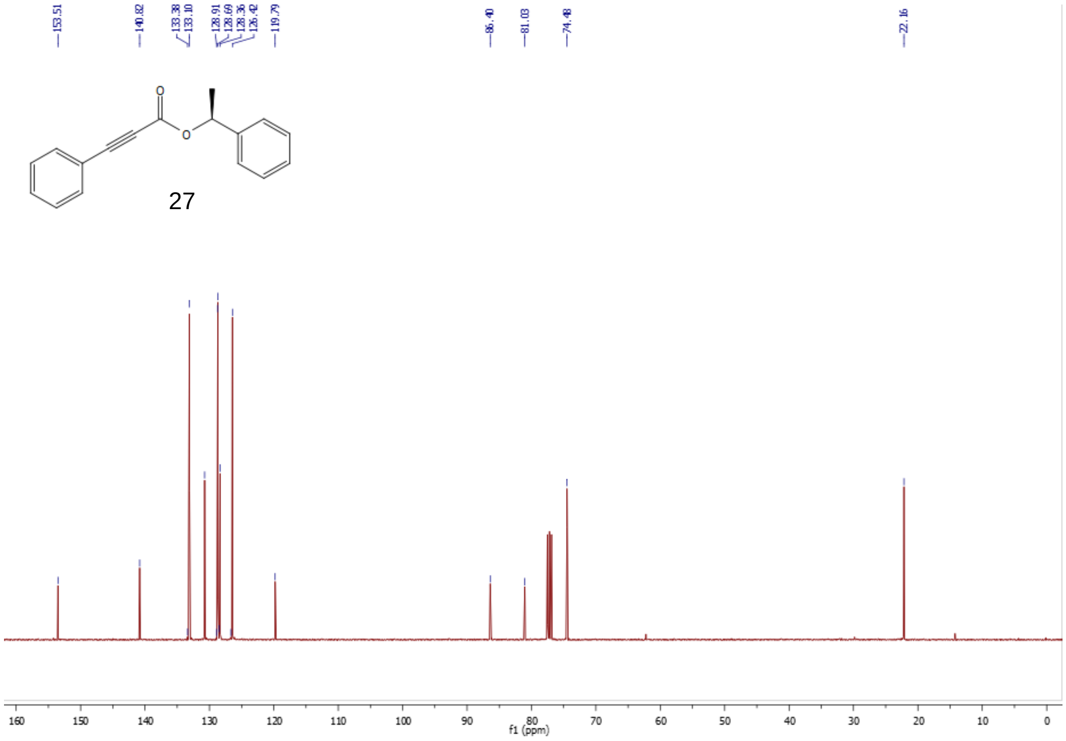
153.51
140.82
133.38
133.10
128.91
128.69
128.36
126.42
119.79
86.40
81.03
74.48
22.16
27
f1 (ppm)

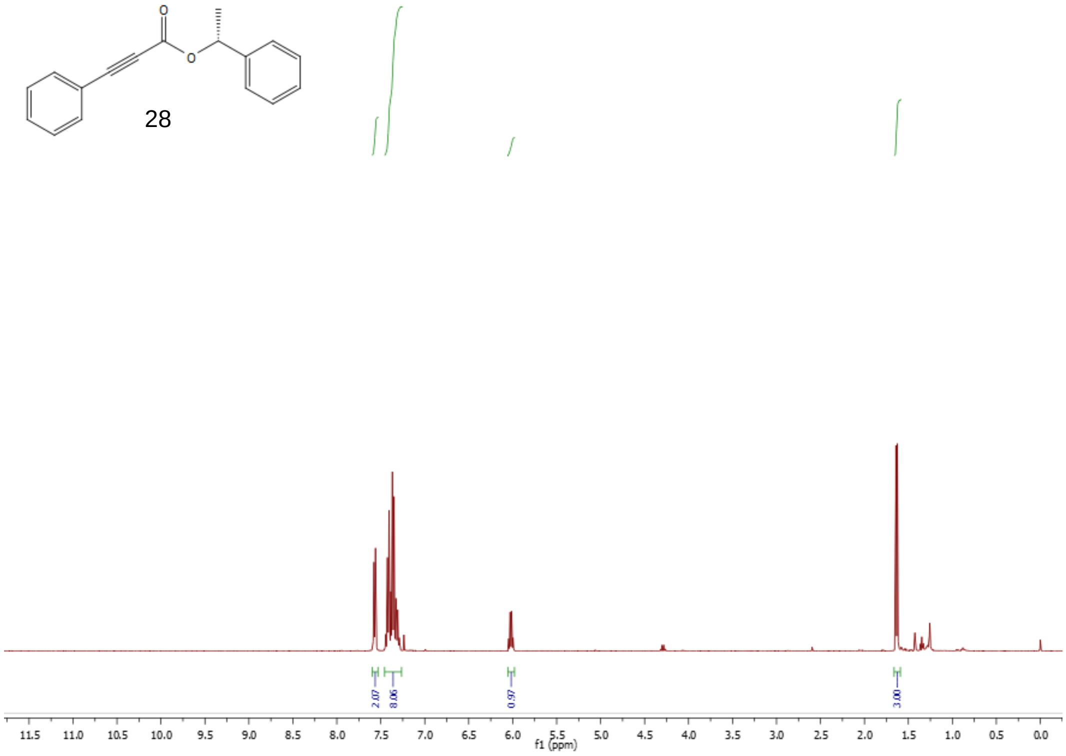
O
O
2.07
8.06
0.97
3.00
11.5 11.0 10.5 10.0 9.5 9.0 8.5 8.0 7.5 7.0 6.5 6.0 5.5 5.0 4.5 4.0 3.5 3.0 2.5 2.0 1.5 1.0 0.5 0.0
f1 (ppm)

28

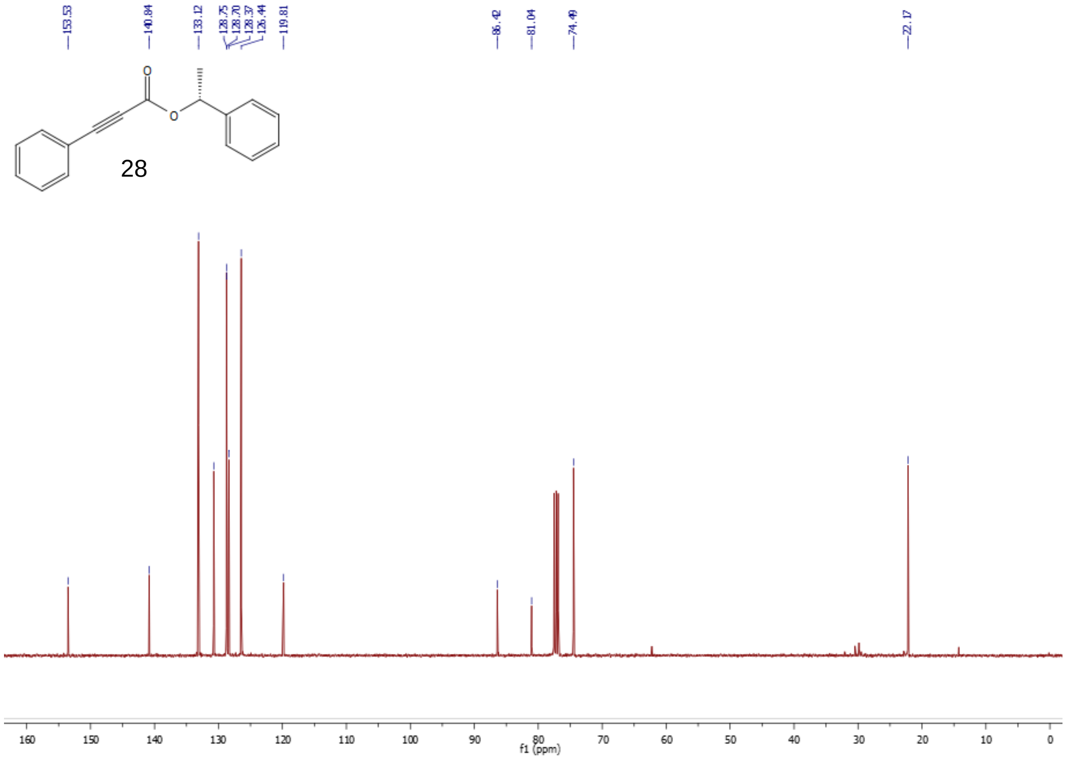
153.53
140.84
133.12
128.75
128.70
128.37
126.44
119.81
86.42
81.04
74.49
22.17
O
O
160 150 140 130 120 110 100 90 80 70 60 50 40 30 20 10 0
f1 (ppm)

28

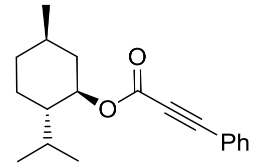
O
O
Ph

29

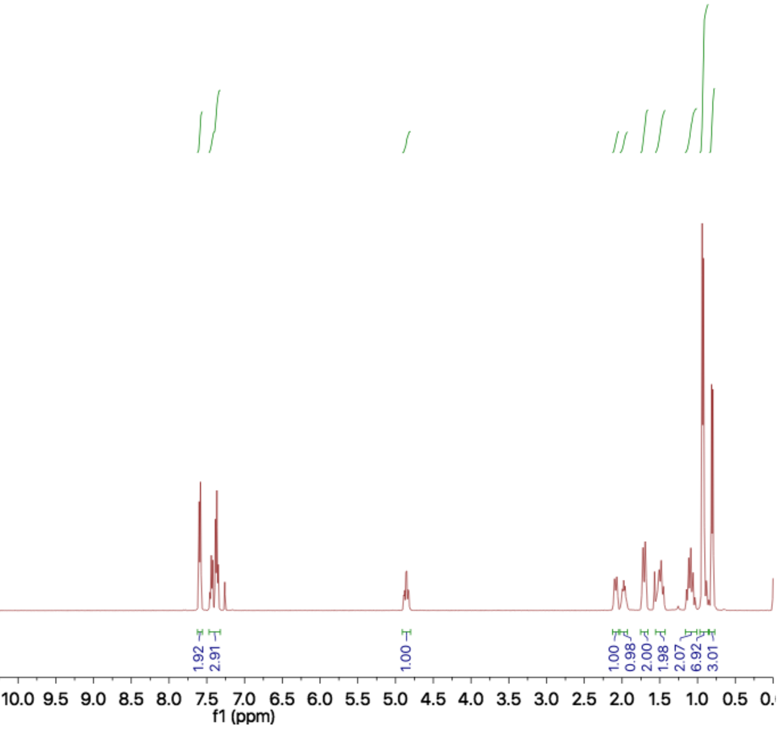
1.92
2.91
1.00
1.00
0.98
2.00
1.98
2.07
6.92
3.01
13.5 13.0 12.5 12.0 11.5 11.0 10.5 10.0 9.5 9.0 8.5 8.0 7.5 7.0 6.5 6.0 5.5 5.0 4.5 4.0 3.5 3.0 2.5 2.0 1.5 1.0 0.5
f1 (ppm)

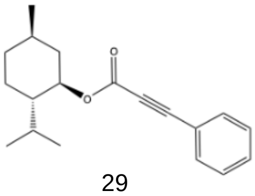
O
O

29

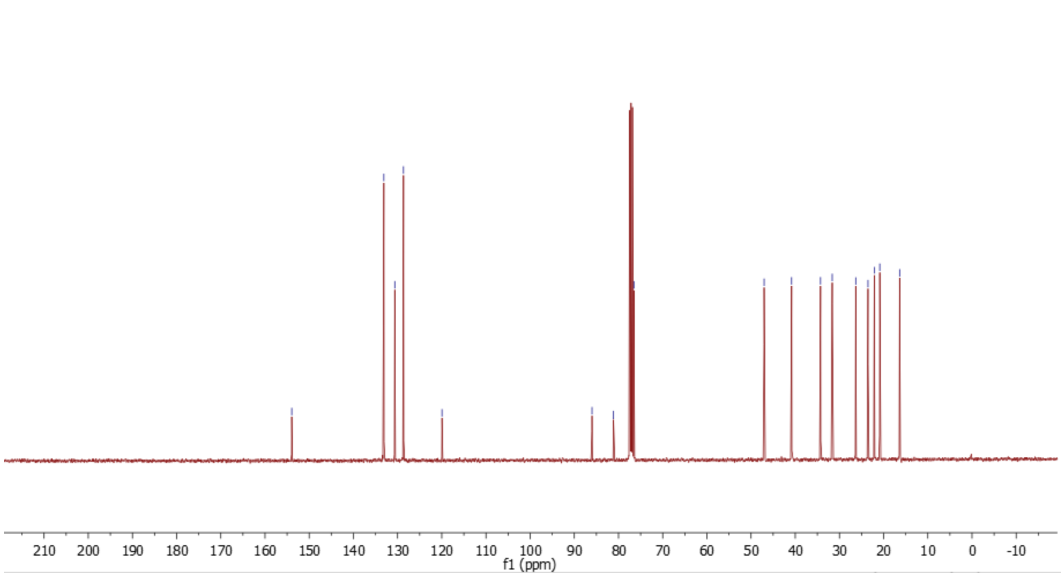
153.95
133.11
130.63
128.66
119.95
86.00
81.15
76.53
47.04
40.83
34.28
31.63
26.32
23.54
22.12
20.88
16.38
210 200 190 180 170 160 150 140 130 120 110 100 90 80 70 60 50 40 30 20 10 0 -10
f1 (ppm)

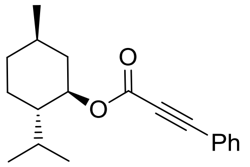

29

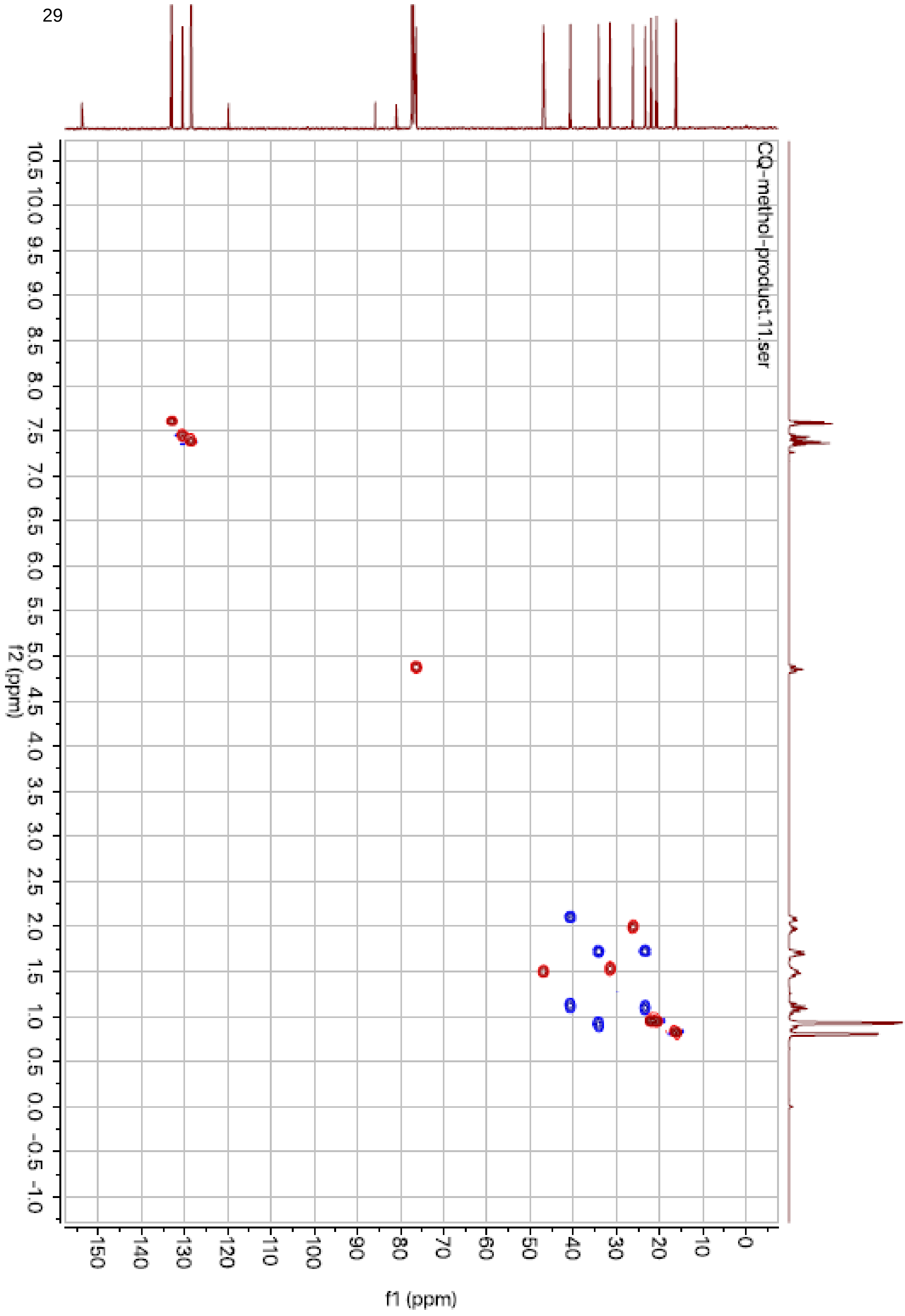

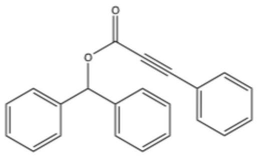
O
O

30

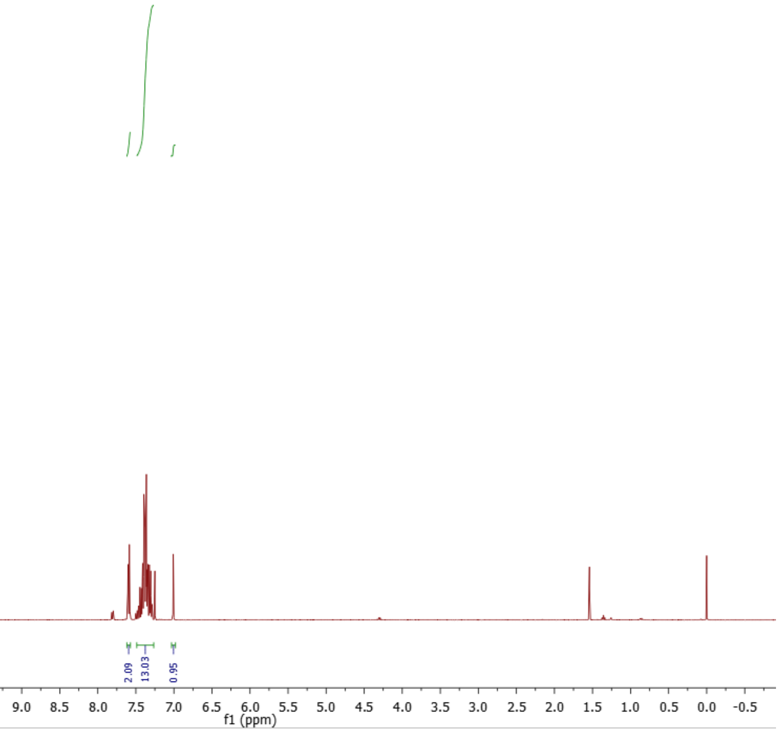
2.09
13.03
0.95
12.5 11.5 10.5 9.5 9.0 8.5 8.0 7.5 7.0 6.5 6.0 5.5 5.0 4.5 4.0 3.5 3.0 2.5 2.0 1.5 1.0 0.5 0.0 -0.5
f1 (ppm)

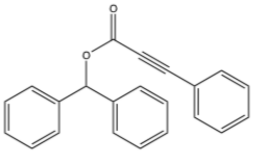
O
O

30

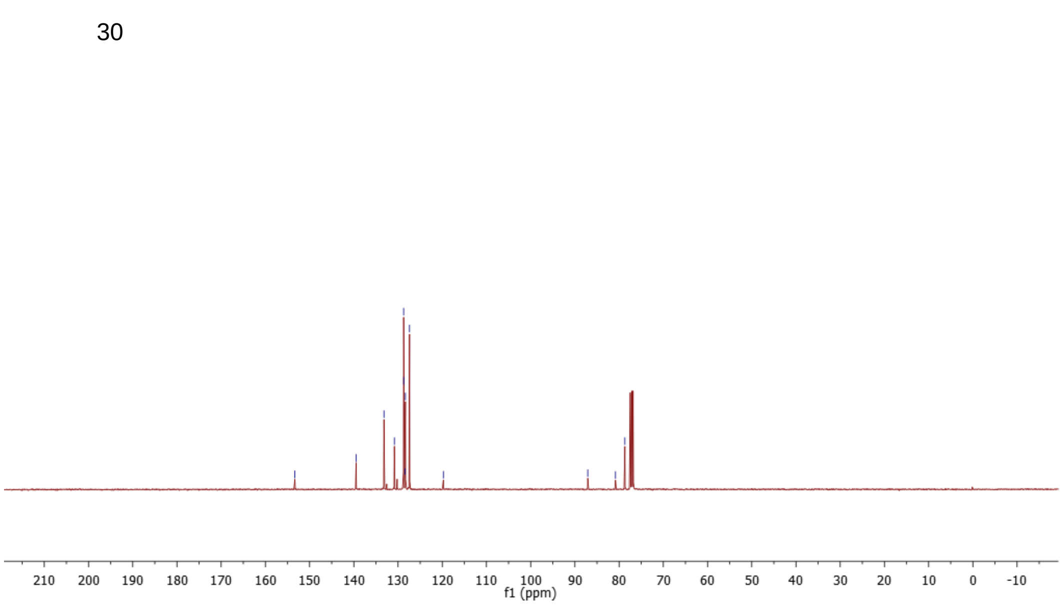
153.33
139.48
133.18
128.73
128.71
128.33
127.40
119.75
87.09
80.82
78.70
210 200 190 180 170 160 150 140 130 120 110 100 90 80 70 60 50 40 30 20 10 0 -10
f1 (ppm)

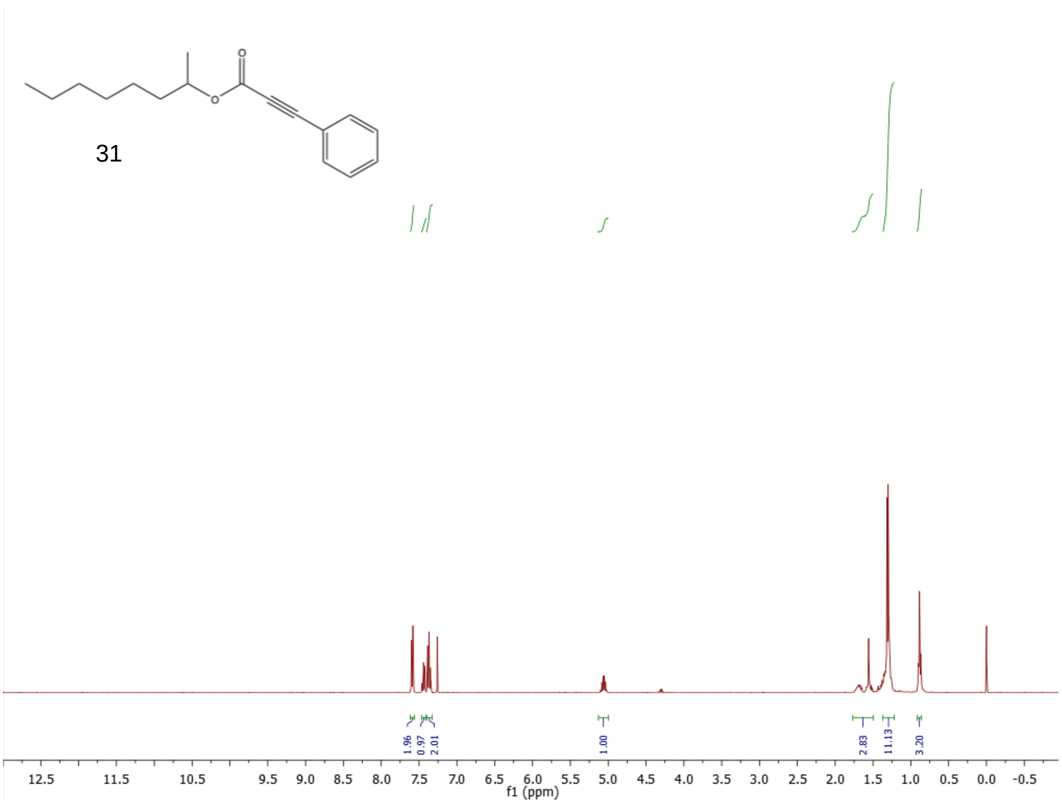
31
1.96
0.97
2.01
1.00
2.83
11.13
3.20
f1 (ppm)

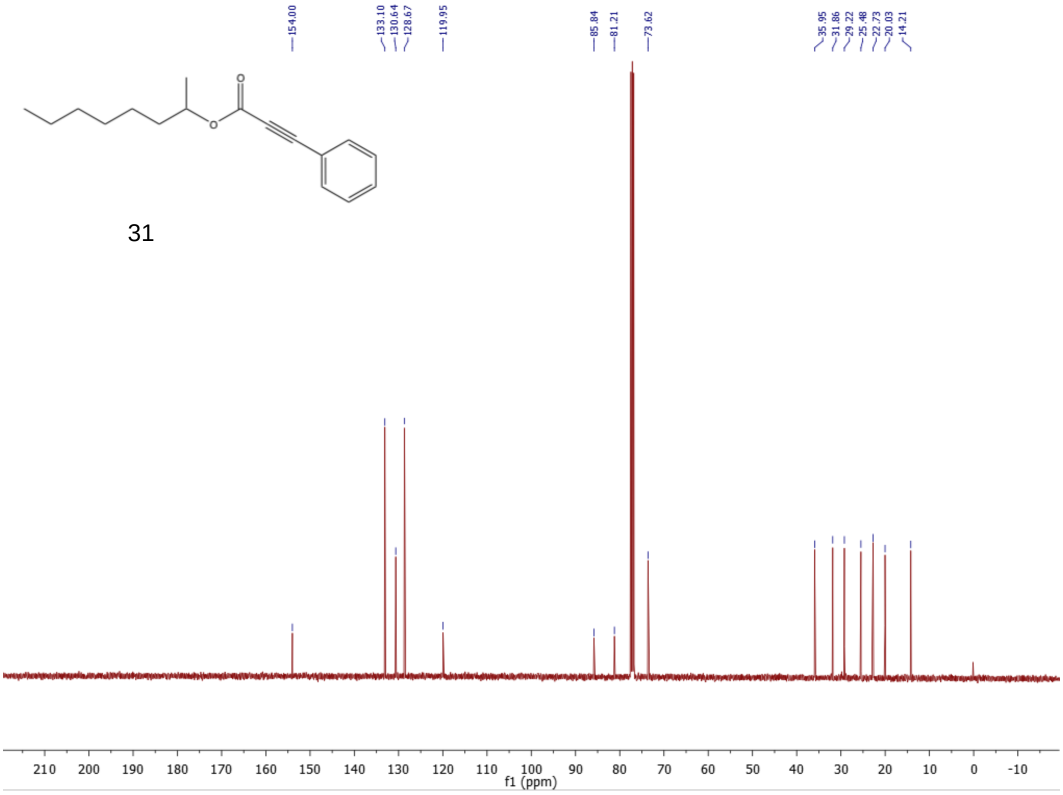
154.00
133.10
130.64
128.67
119.95
85.84
81.21
73.62
35.95
31.86
29.22
25.48
22.73
20.03
14.21
31
f1 (ppm)

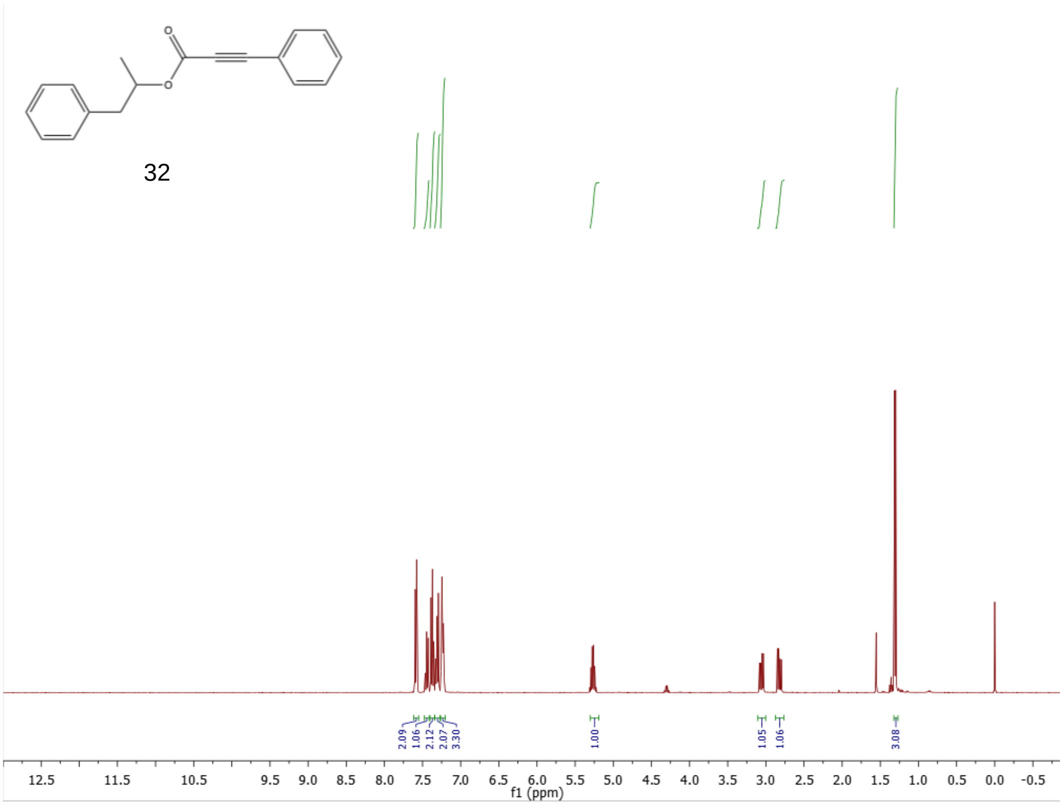
32
2.09
1.06
2.12
2.07
3.30
1.00
1.05
1.06
3.08
12.5 11.5 10.5 9.5 9.0 8.5 8.0 7.5 7.0 6.5 6.0 5.5 5.0 4.5 4.0 3.5 3.0 2.5 2.0 1.5 1.0 0.5 0.0 -0.5
f1 (ppm)

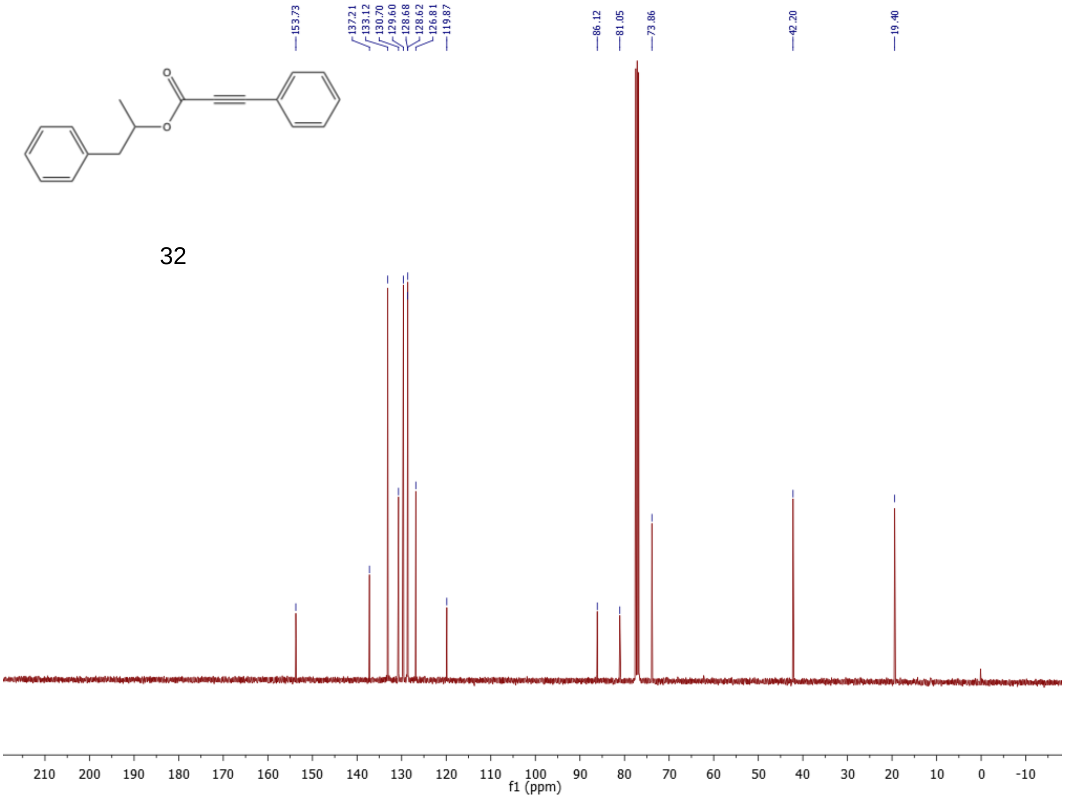
153.73
137.21
133.12
130.70
129.60
128.68
128.62
126.81
119.87
86.12
81.05
73.86
42.20
19.40
32
210 200 190 180 170 160 150 140 130 120 110 100 90 80 70 60 50 40 30 20 10 0 -10
f1 (ppm)

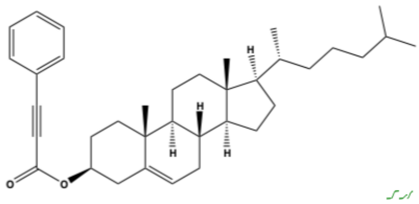

33

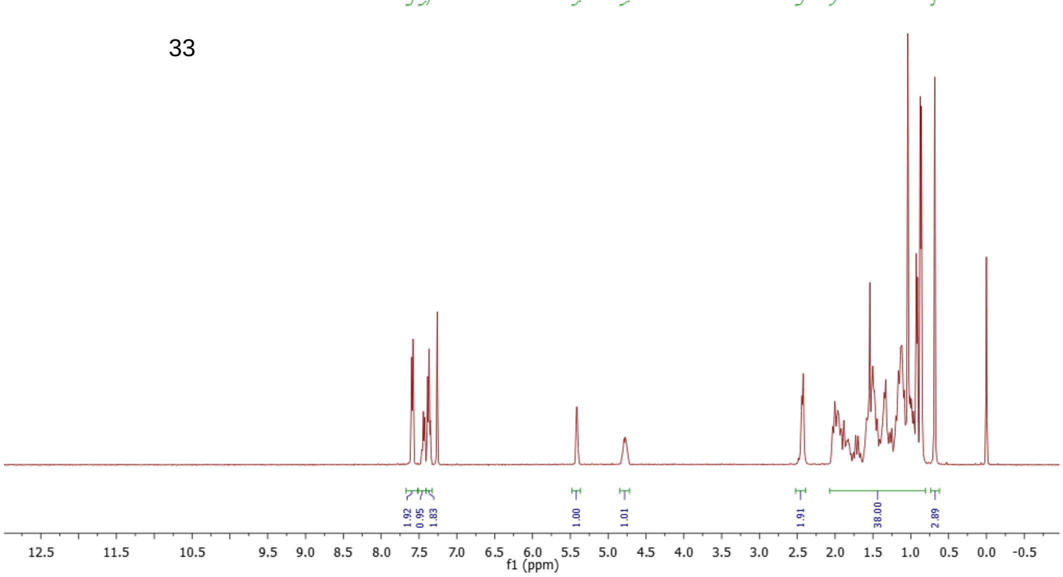
1.92
0.95
1.83
1.00
1.01
1.91
38.00
2.89
f1 (ppm)

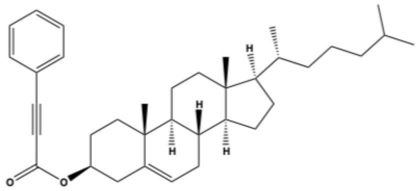

33

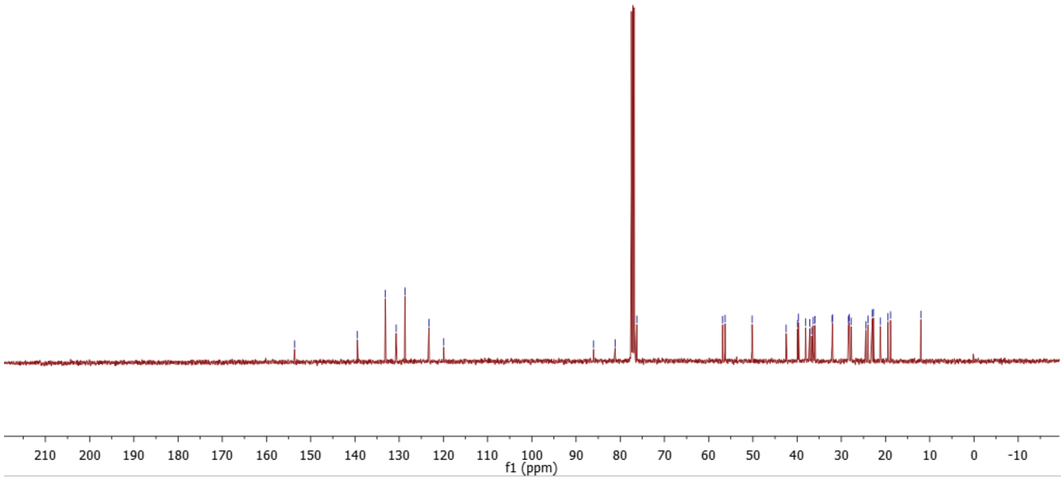
153.68
139.44
133.12
130.68
128.69
123.26
119.91
86.02
81.16
76.25
56.84
56.30
50.18
39.68
35.95
32.07
32.01
28.38
28.17
23.99
22.97
22.72
19.45
f1 (ppm)

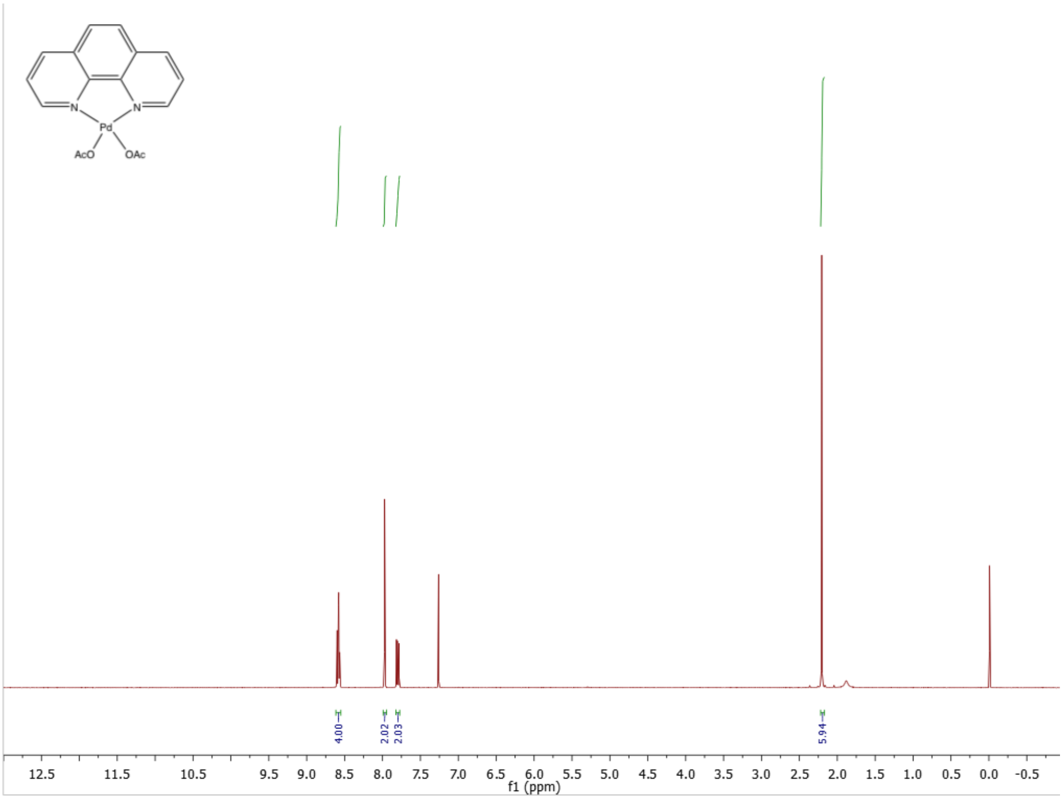
N
Pd
AcO
OAc
4.00
2.02
2.03
5.94
12.5 11.5 10.5 9.5 9.0 8.5 8.0 7.5 7.0 6.5 6.0 5.5 5.0 4.5 4.0 3.5 3.0 2.5 2.0 1.5 1.0 0.5 0.0 -0.5
f1 (ppm)

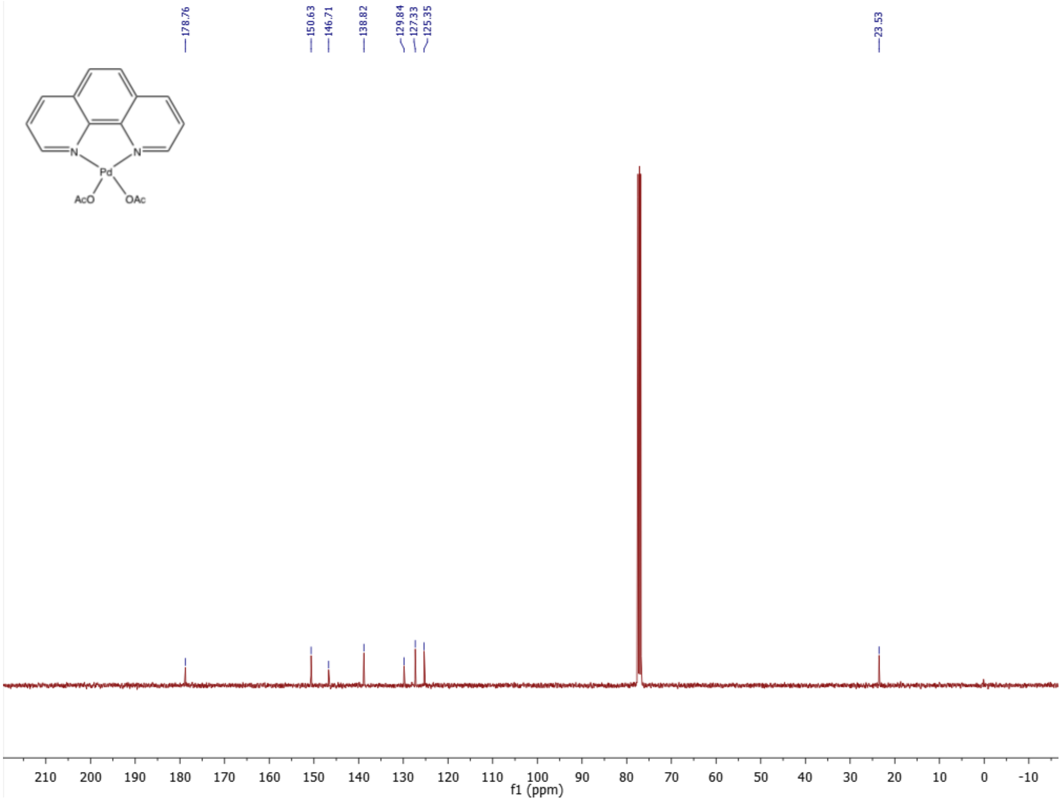
178.76
150.63
146.71
138.82
129.84
127.33
125.35
23.53
N
Pd
AcO
OAc
210 200 190 180 170 160 150 140 130 120 110 100 90 80 70 60 50 40 30 20 10 0 -10
f1 (ppm)

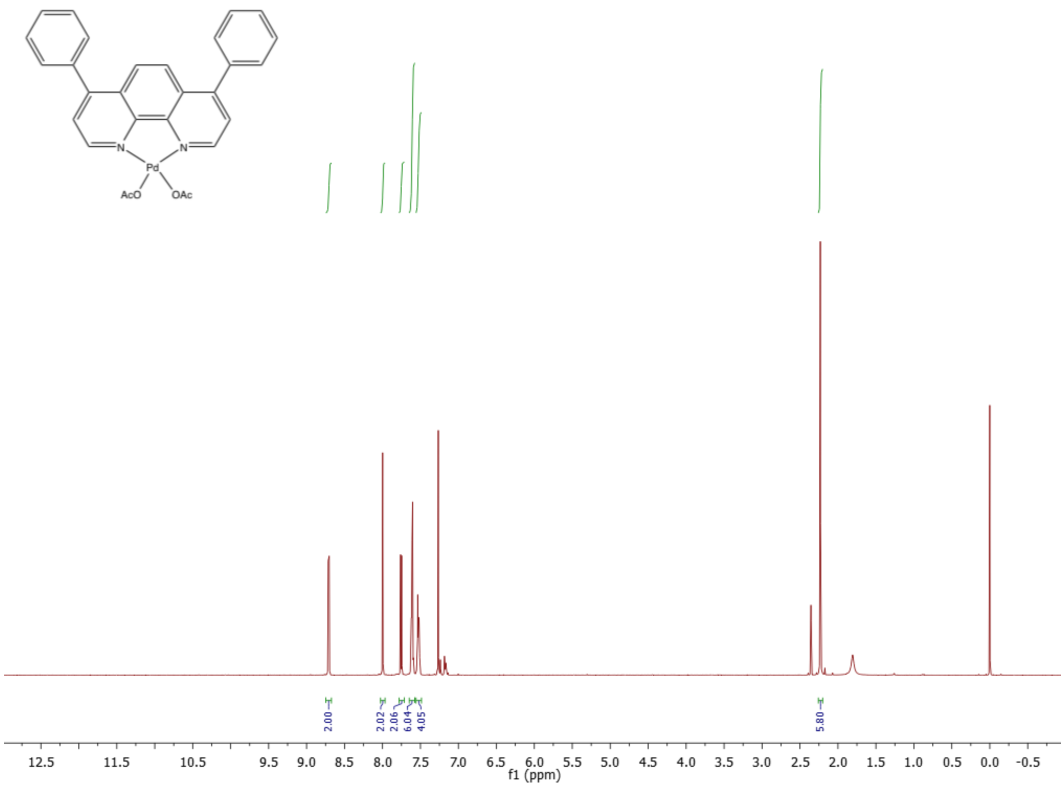

N
N
Pd
AcO
OAc
2.00
2.02
2.06
6.04
4.05
5.80
12.5
11.5
10.5
9.5
9.0
8.5
8.0
7.5
7.0
6.5
6.0
5.5
5.0
4.5
4.0
3.5
3.0
2.5
2.0
1.5
1.0
0.5
0.0
-0.5
f1 (ppm)

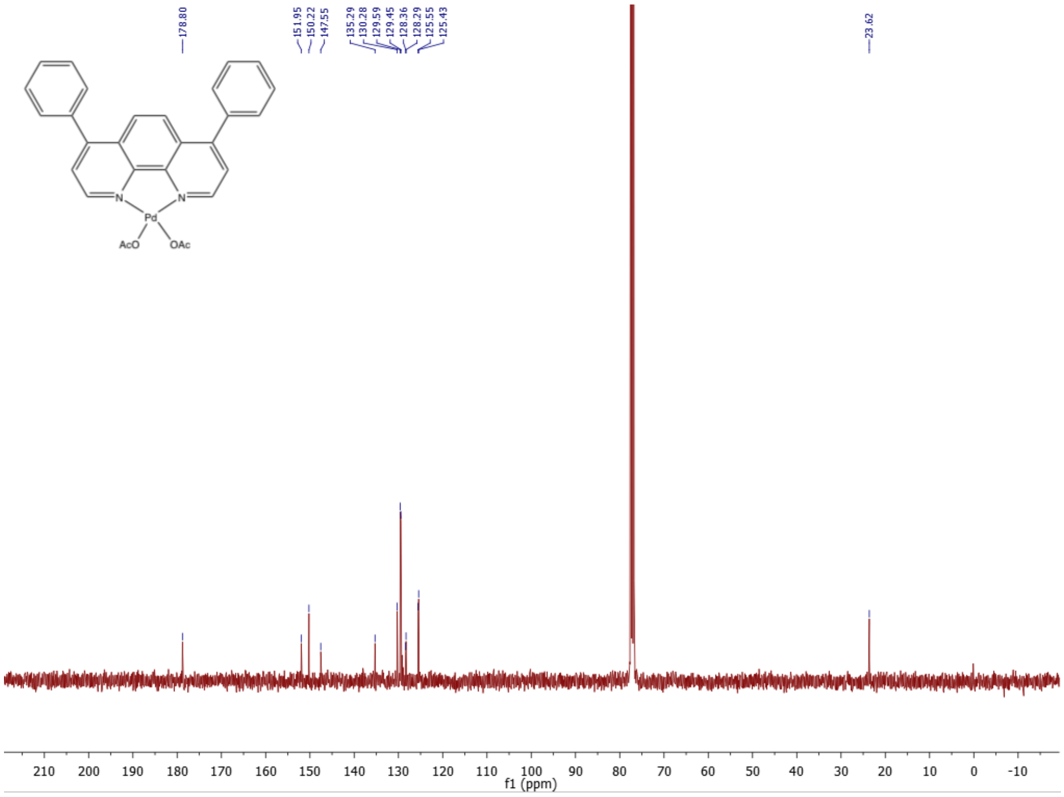

178.80
151.95
150.22
147.55
135.29
130.28
129.59
129.45
128.36
128.29
125.55
125.43
23.62
N
N
Pd
AcO
OAc
210
200
190
180
170
160
150
140
130
120
110
100
90
80
70
60
50
40
30
20
10
0
-10
f1 (ppm)

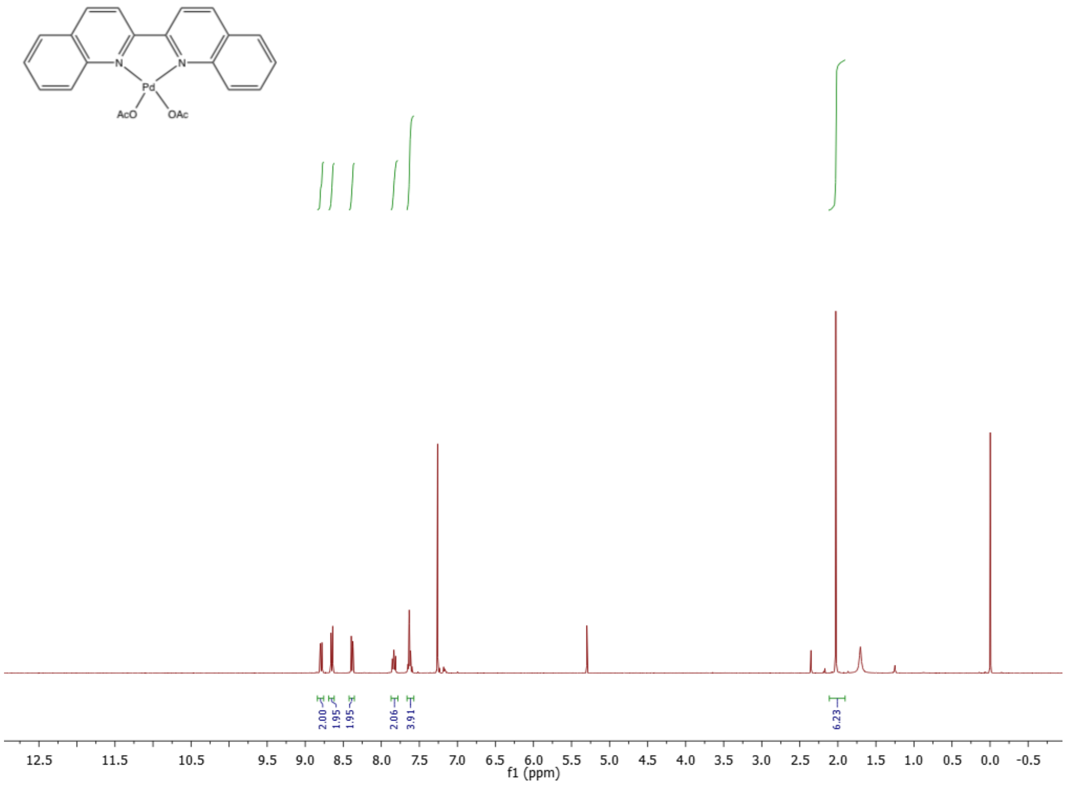
N
N
Pd
AcO
OAc
2.00
1.95
1.95
2.06
3.91
6.23
12.5
11.5
10.5
9.5
9.0
8.5
8.0
7.5
7.0
6.5
6.0
5.5
5.0
4.5
4.0
3.5
3.0
2.5
2.0
1.5
1.0
0.5
0.0
-0.5
f1 (ppm)

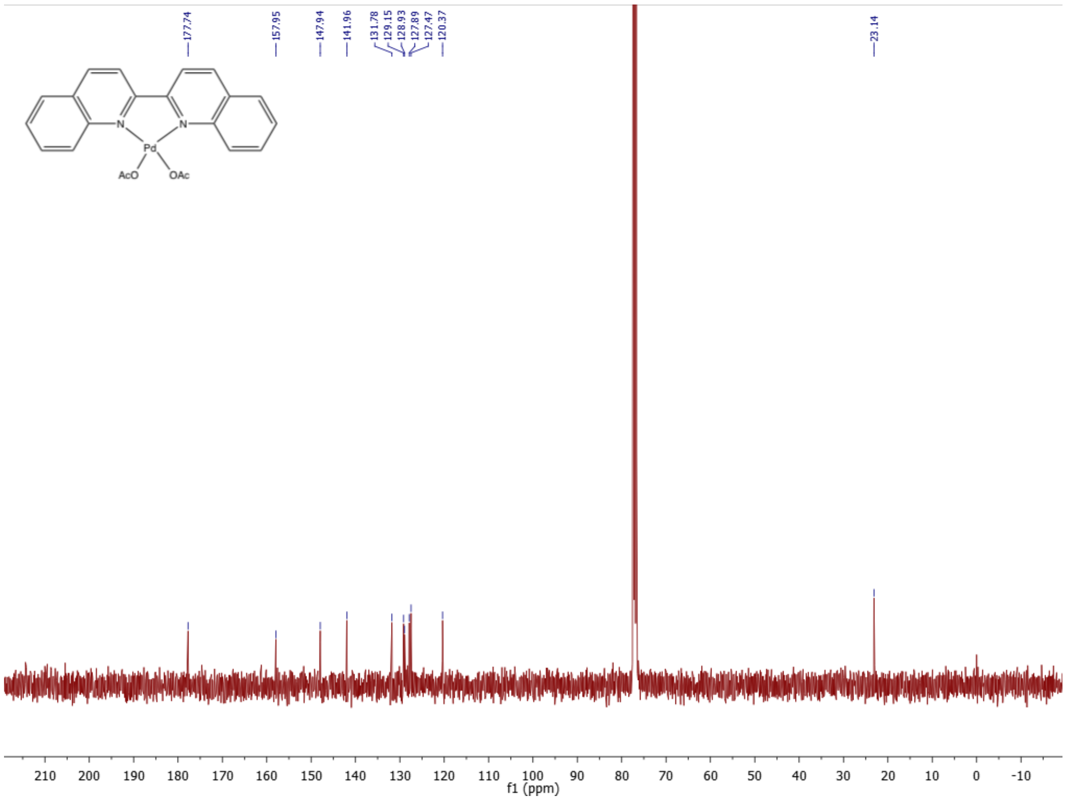
177.74
157.95
147.94
141.96
131.78
129.15
128.93
127.89
127.47
120.37
23.14
N
N
Pd
AcO
OAc
210
200
190
180
170
160
150
140
130
120
110
100
90
80
70
60
50
40
30
20
10
0
-10
f1 (ppm)

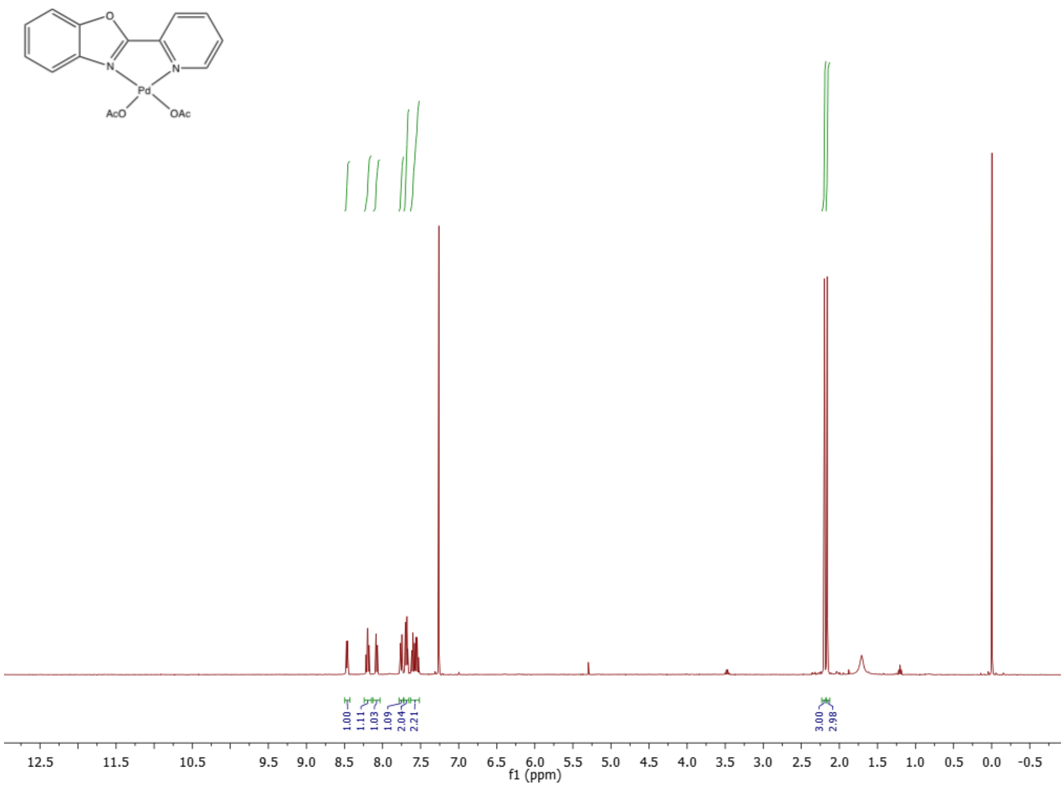
O
N
N
Pd
AcO
OAc
1.00
1.11
1.03
1.09
2.04
2.21
3.00
2.98
12.5 11.5 10.5 9.5 9.0 8.5 8.0 7.5 7.0 6.5 6.0 5.5 5.0 4.5 4.0 3.5 3.0 2.5 2.0 1.5 1.0 0.5 0.0 -0.5
f1 (ppm)

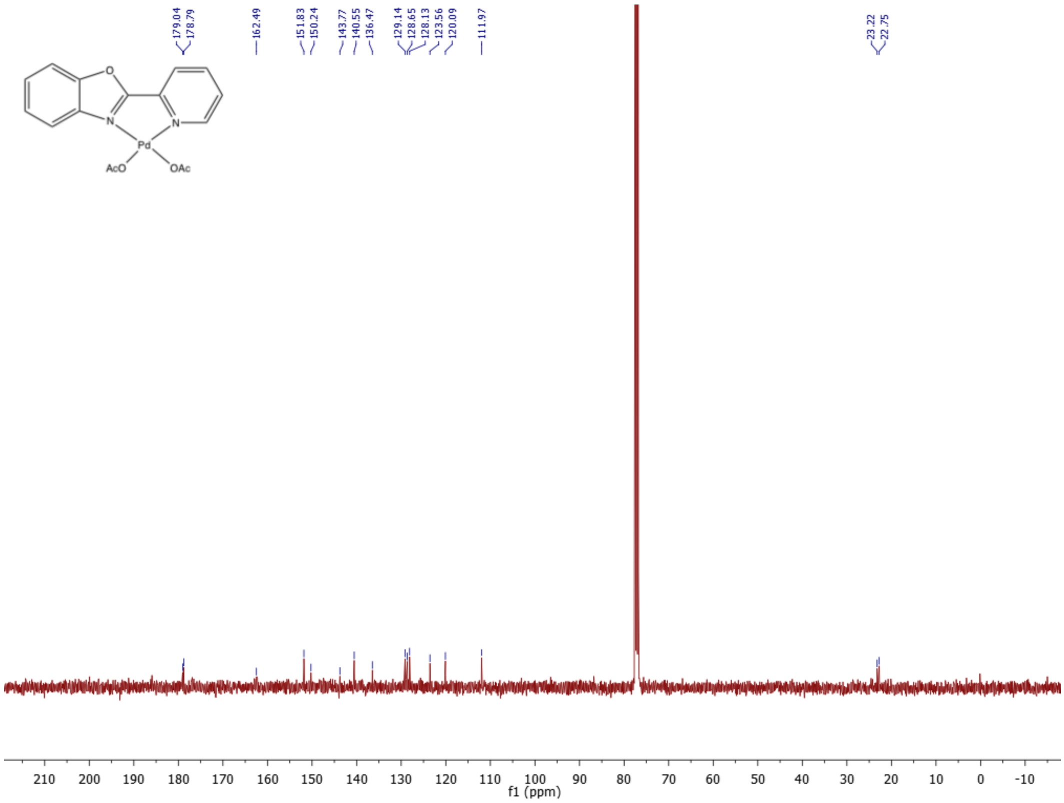
179.04
178.79
162.49
151.83
150.24
143.77
140.55
136.47
129.14
128.65
128.13
123.56
120.09
111.97
23.22
22.75
O
N
N
Pd
AcO
OAc
210 200 190 180 170 160 150 140 130 120 110 100 90 80 70 60 50 40 30 20 10 0 -10
f1 (ppm)

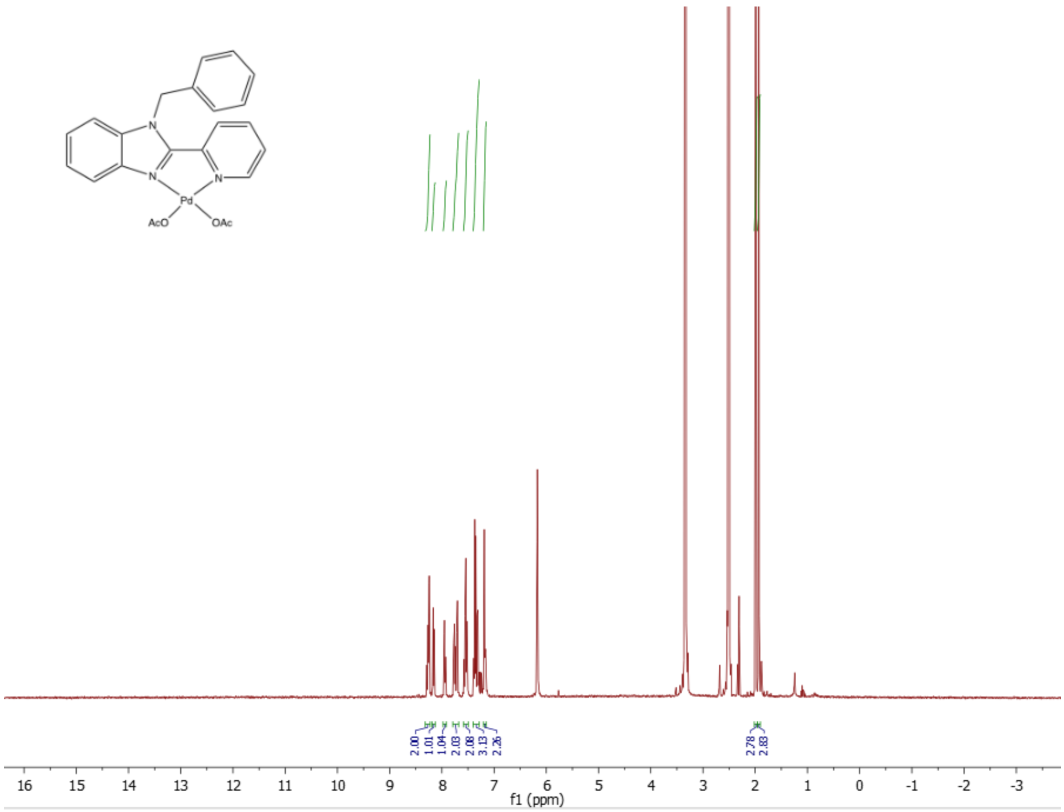
N
N
N
Pd
AcO
OAc
2.00
1.01
1.04
2.03
2.08
3.13
2.26
2.78
2.83
16
15
14
13
12
11
10
9
8
7
6
5
4
3
2
1
0
-1
-2
-3
f1 (ppm)

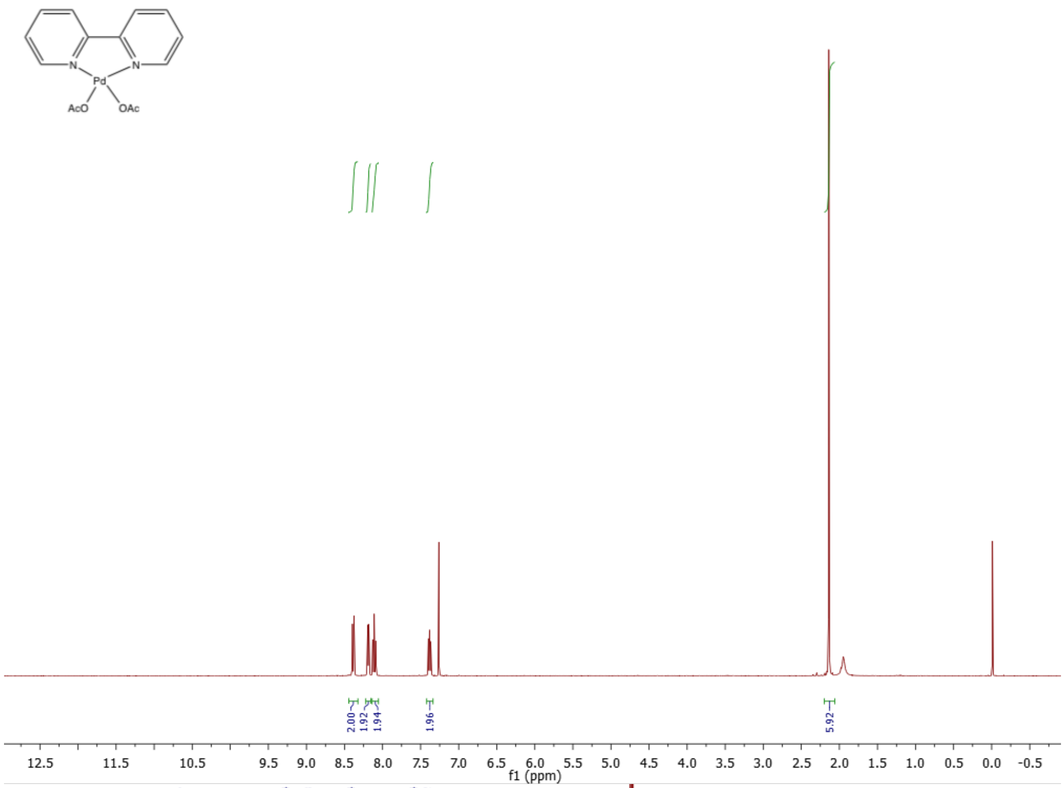
N
Pd
N
AcO
OAc
2.00
1.92
1.94
1.96
5.92
f1 (ppm)

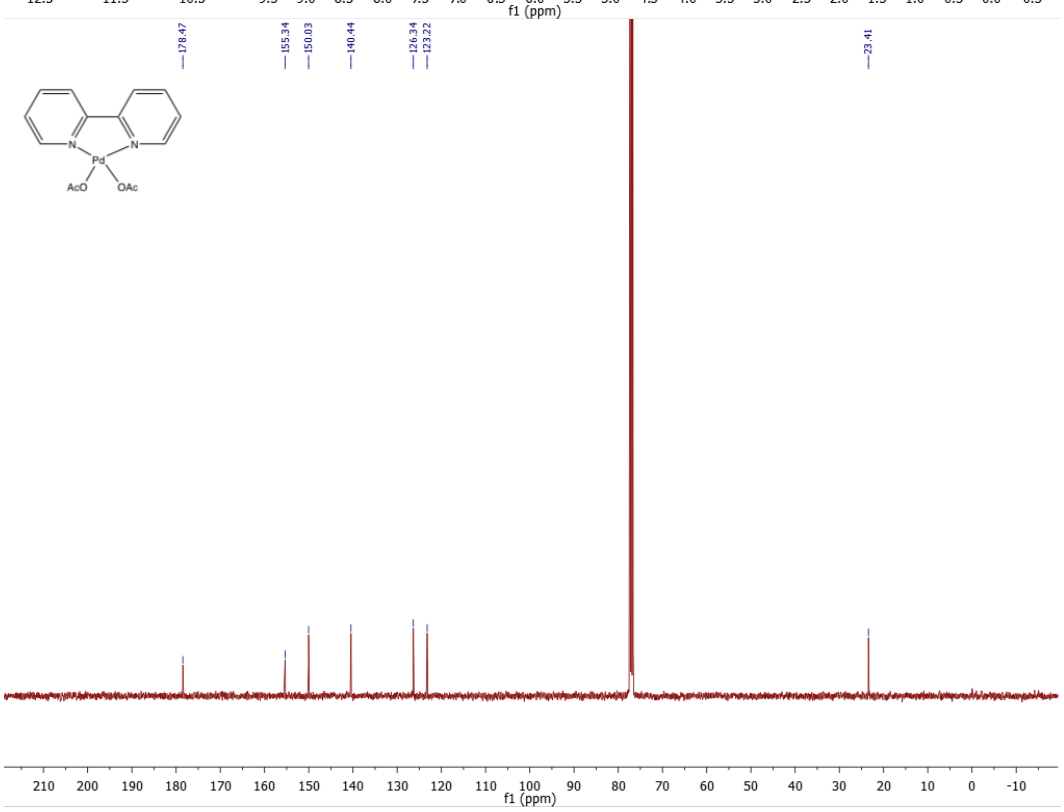
178.47
155.34
150.03
140.44
126.34
123.22
23.41
N
Pd
N
AcO
OAc
f1 (ppm)

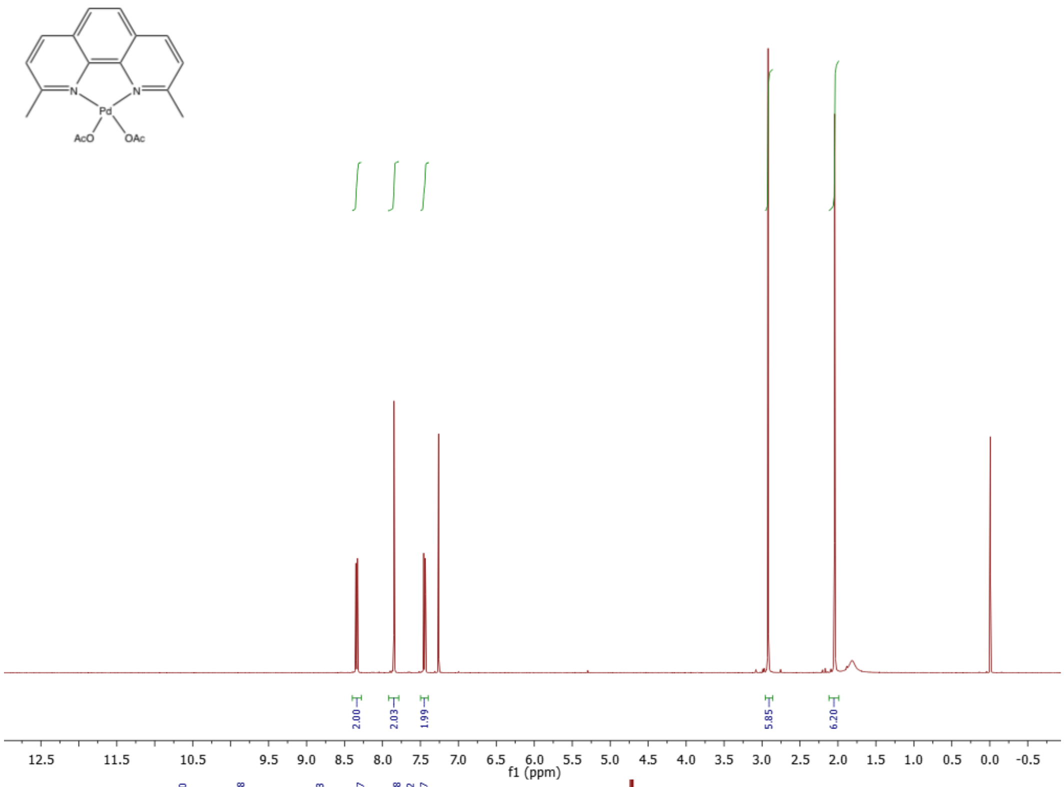
Pd
AcO
OAc
N
2.00
2.03
1.99
5.85
6.20
12.5 11.5 10.5 9.5 9.0 8.5 8.0 7.5 7.0 6.5 6.0 5.5 5.0 4.5 4.0 3.5 3.0 2.5 2.0 1.5 1.0 0.5 0.0 -0.5
f1 (ppm)

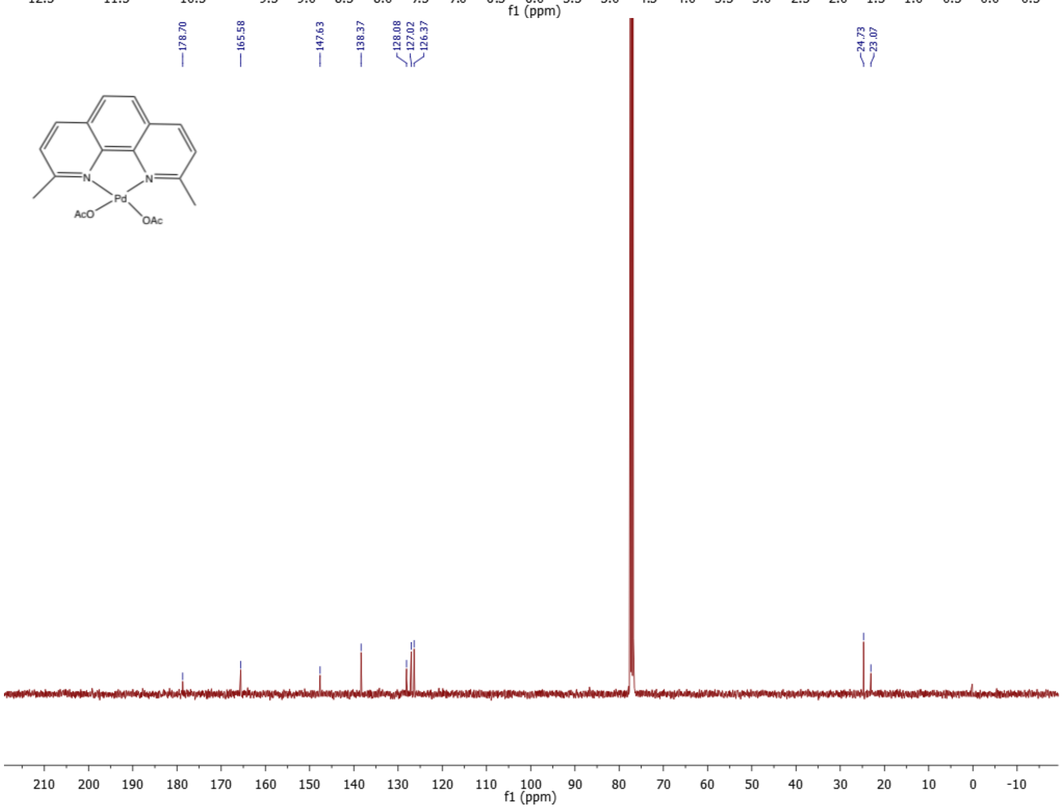
178.70
165.58
147.63
138.37
128.08
127.02
126.37
24.73
23.07
Pd
AcO
OAc
N
210 200 190 180 170 160 150 140 130 120 110 100 90 80 70 60 50 40 30 20 10 0 -10
f1 (ppm)

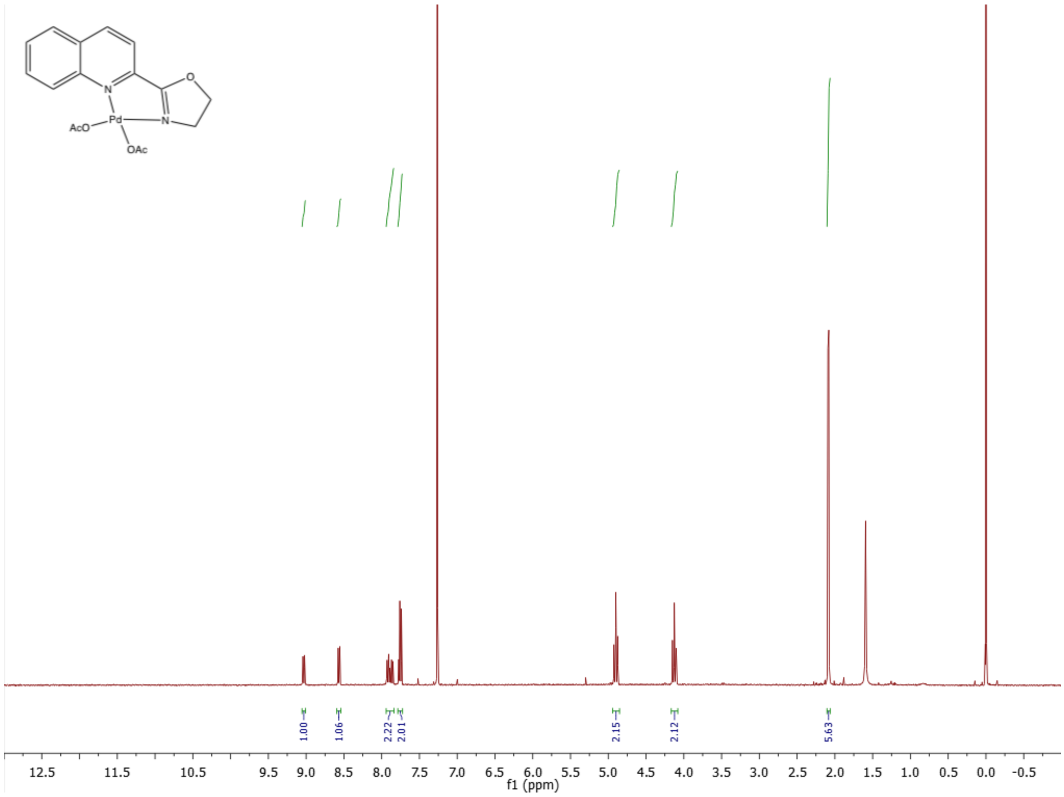
AcO
OAc
1.00
1.06
2.22
2.01
2.15
2.12
5.63
f1 (ppm)

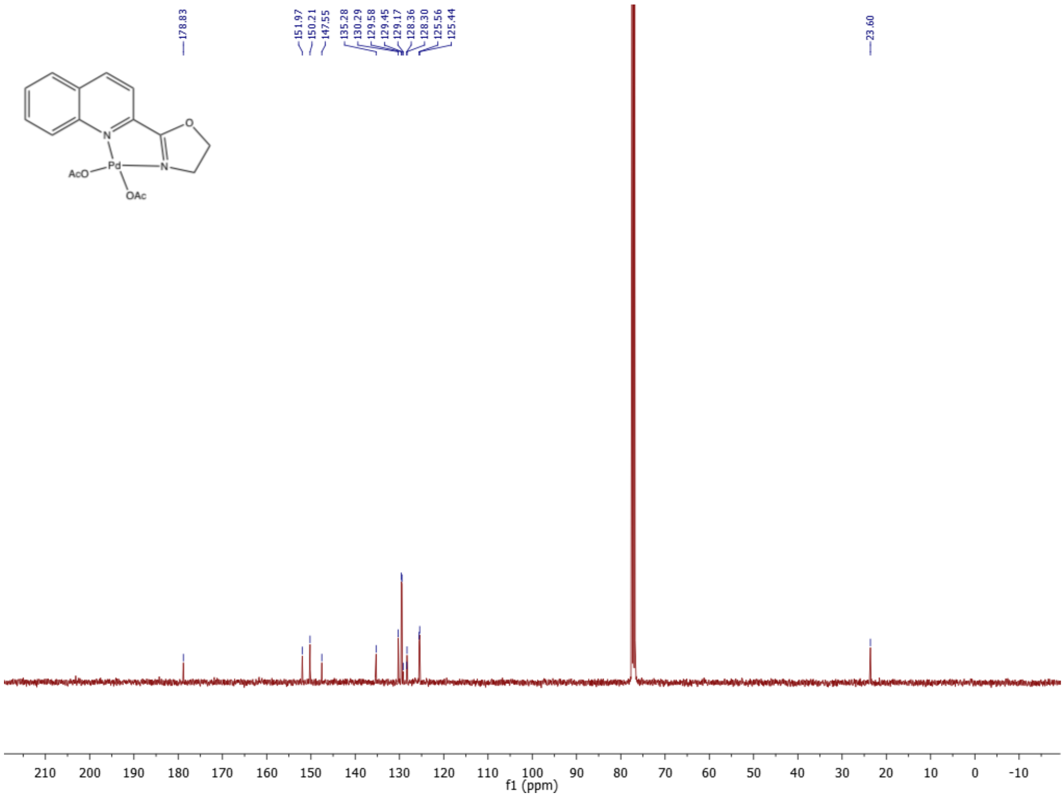
178.83
151.97
150.21
147.55
135.28
130.29
129.58
129.45
129.17
128.36
128.30
125.56
125.44
23.60
AcO
OAc
f1 (ppm)

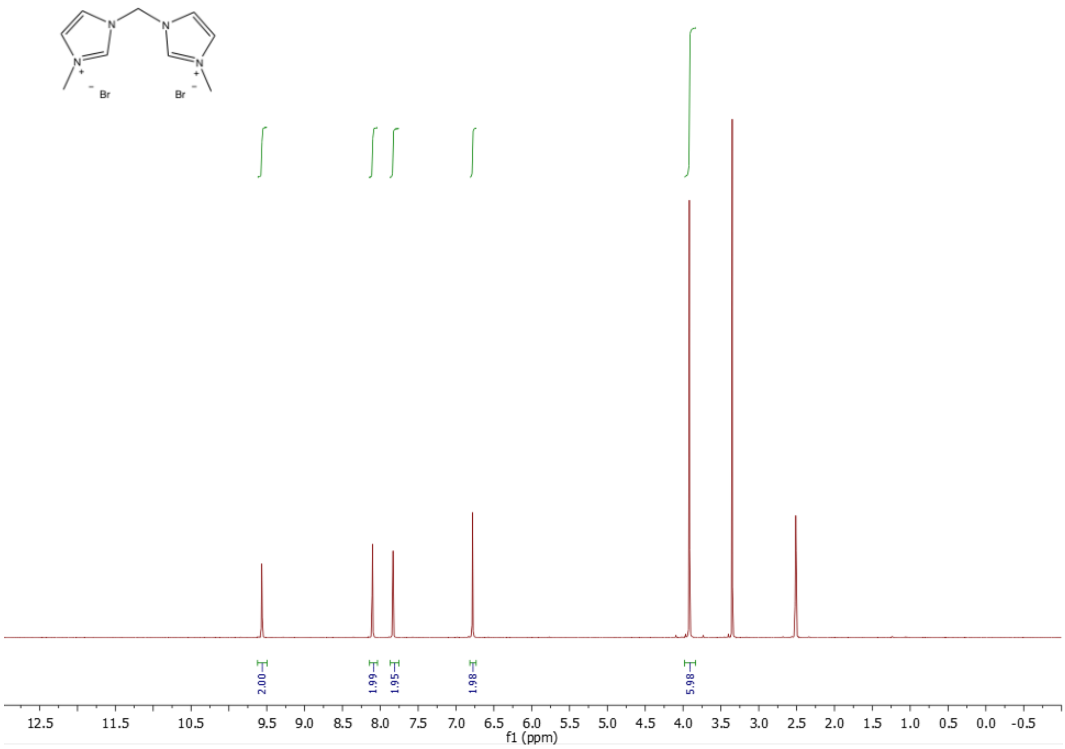

N
N
Br
Br
2.00
1.99
1.95
1.98
5.98
12.5 11.5 10.5 9.5 9.0 8.5 8.0 7.5 7.0 6.5 6.0 5.5 5.0 4.5 4.0 3.5 3.0 2.5 2.0 1.5 1.0 0.5 0.0 -0.5
f1 (ppm)

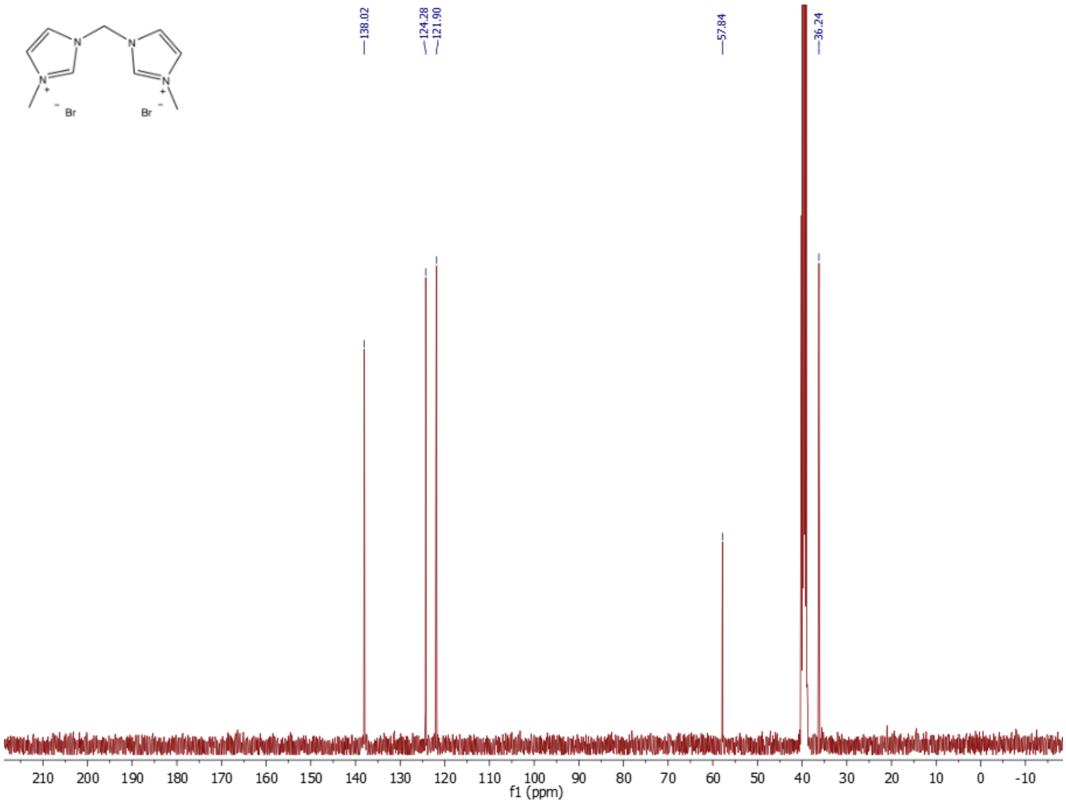

138.02
124.28
121.90
57.84
36.24
N
N
Br
Br
210 200 190 180 170 160 150 140 130 120 110 100 90 80 70 60 50 40 30 20 10 0 -10
f1 (ppm)

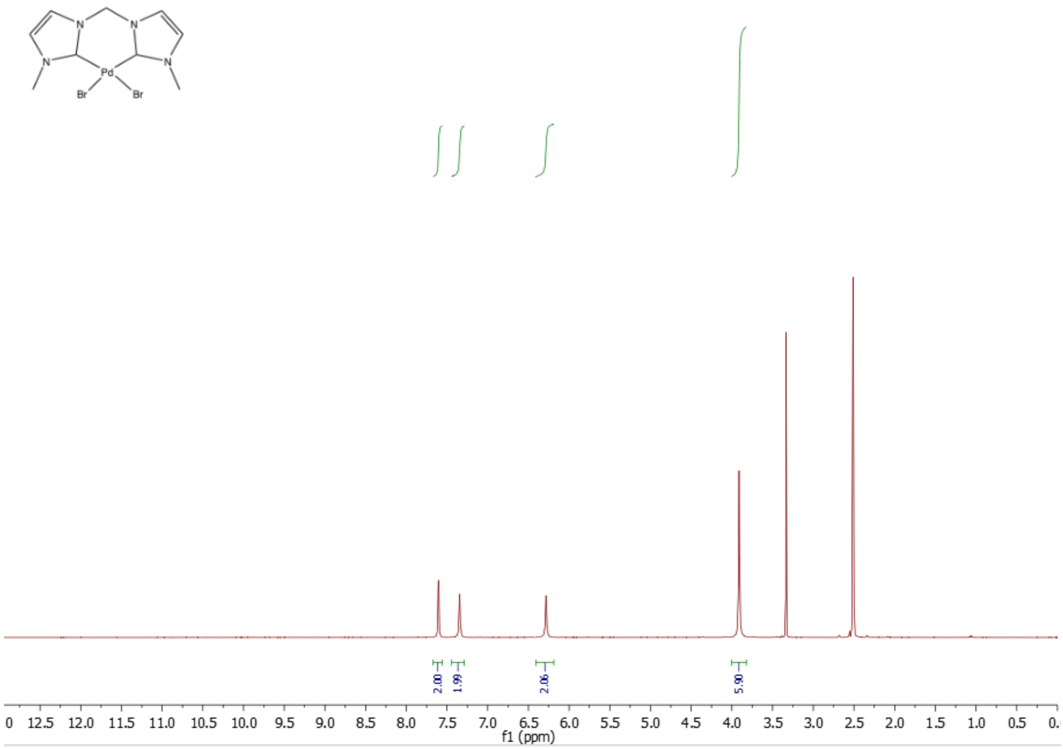
N
N
N
N
Pd
Br
Br
2.00
1.99
2.06
5.90
0 12.5 12.0 11.5 11.0 10.5 10.0 9.5 9.0 8.5 8.0 7.5 7.0 6.5 6.0 5.5 5.0 4.5 4.0 3.5 3.0 2.5 2.0 1.5 1.0 0.5
f1 (ppm)

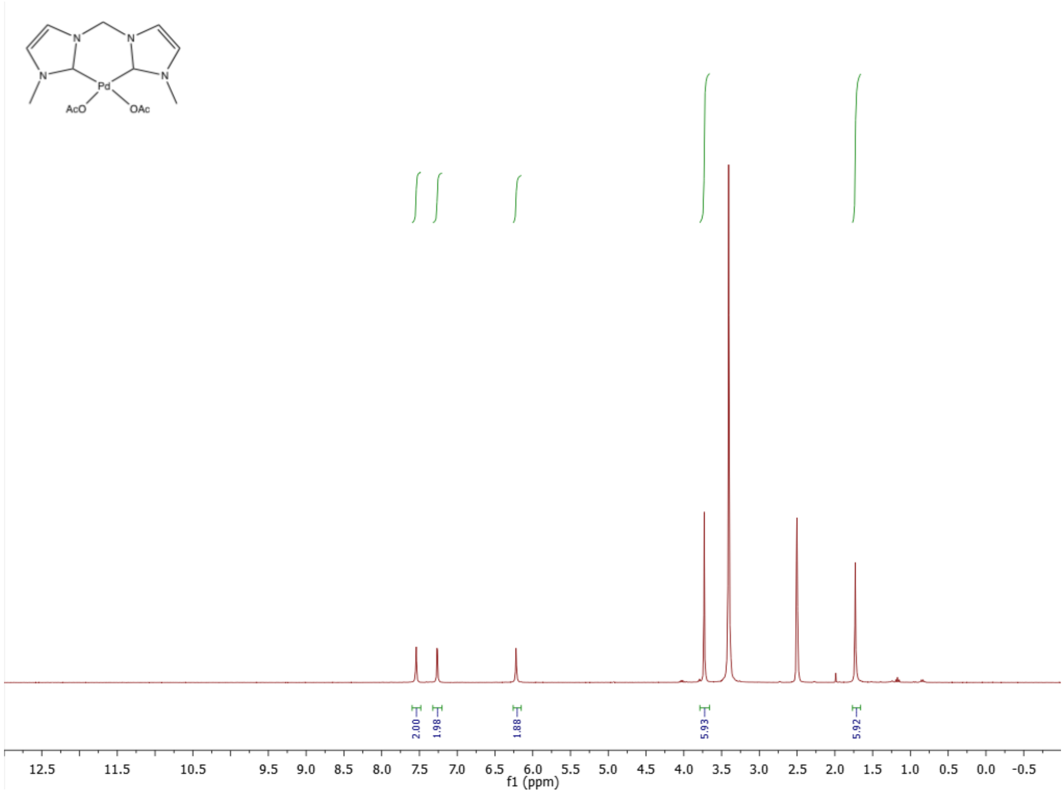
N
N
N
N
Pd
AcO
OAc
2.00
1.98
1.88
5.93
5.92
12.5 11.5 10.5 9.5 9.0 8.5 8.0 7.5 7.0 6.5 6.0 5.5 5.0 4.5 4.0 3.5 3.0 2.5 2.0 1.5 1.0 0.5 0.0 -0.5
f1 (ppm)

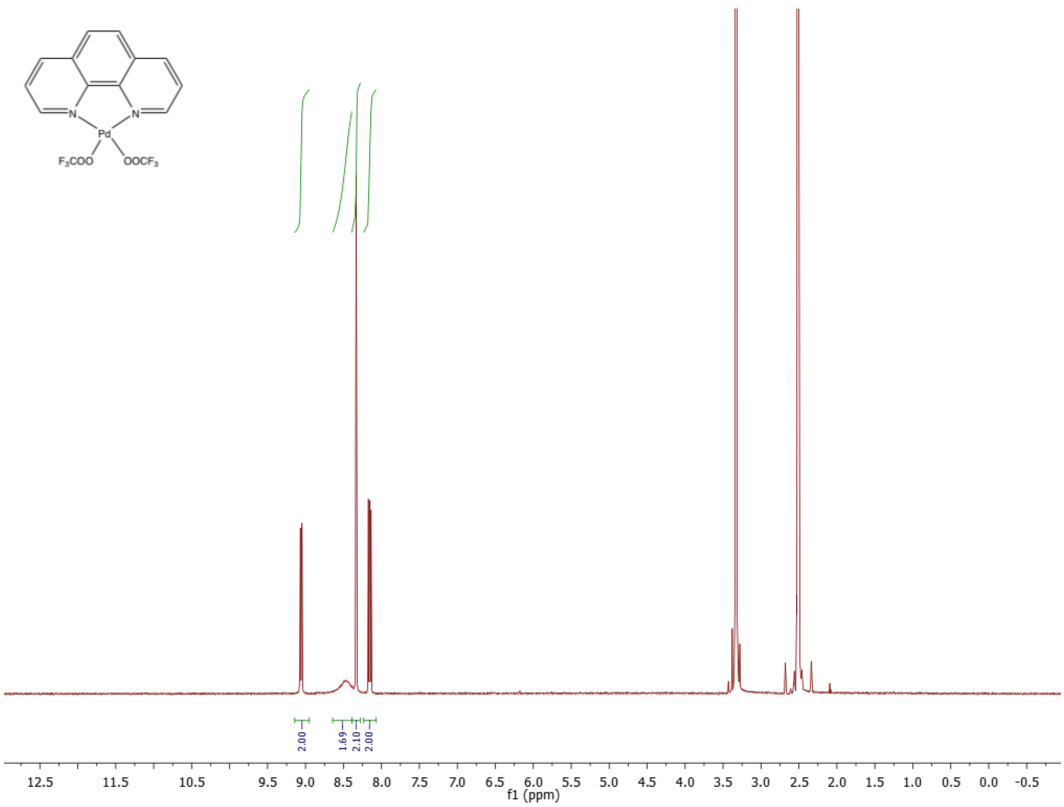

N
N
Pd
F3COO
OOCF3
2.00
1.69
2.10
2.00
12.5
11.5
10.5
9.5
9.0
8.5
8.0
7.5
7.0
6.5
6.0
5.5
5.0
4.5
4.0
3.5
3.0
2.5
2.0
1.5
1.0
0.5
0.0
-0.5
f1 (ppm)

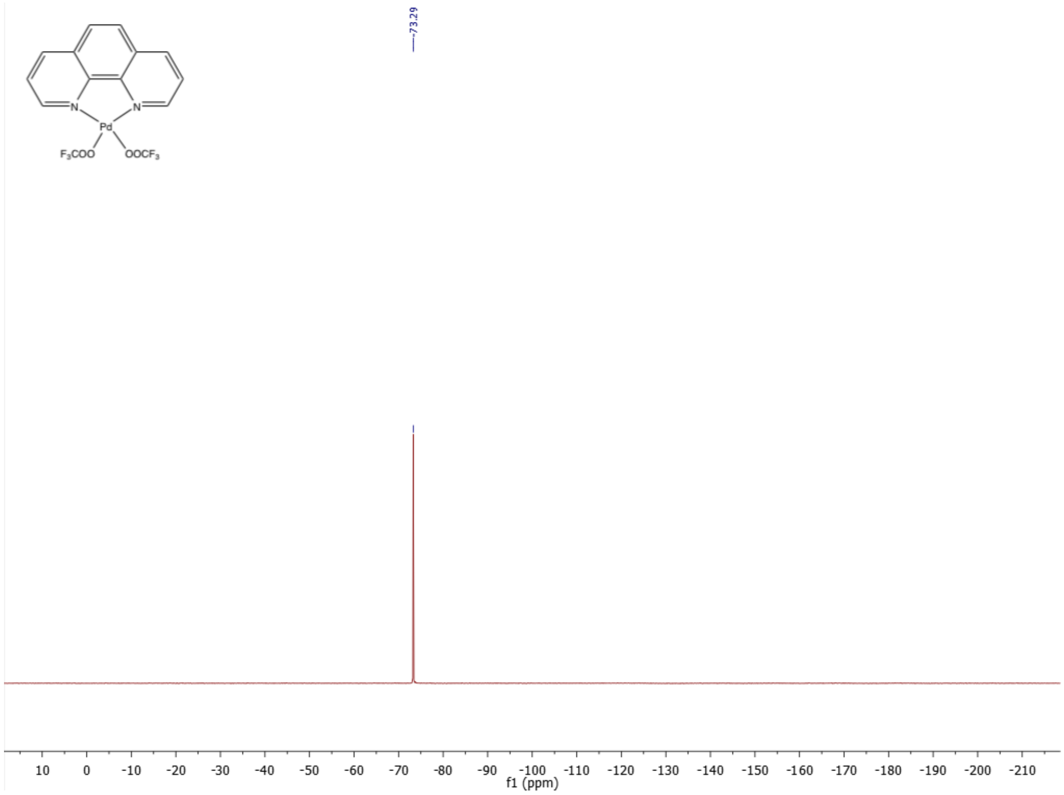

-73.29
N
N
Pd
F3COO
OOCF3
10
0
-10
-20
-30
-40
-50
-60
-70
-80
-90
-100
-110
-120
-130
-140
-150
-160
-170
-180
-190
-200
-210
f1 (ppm)

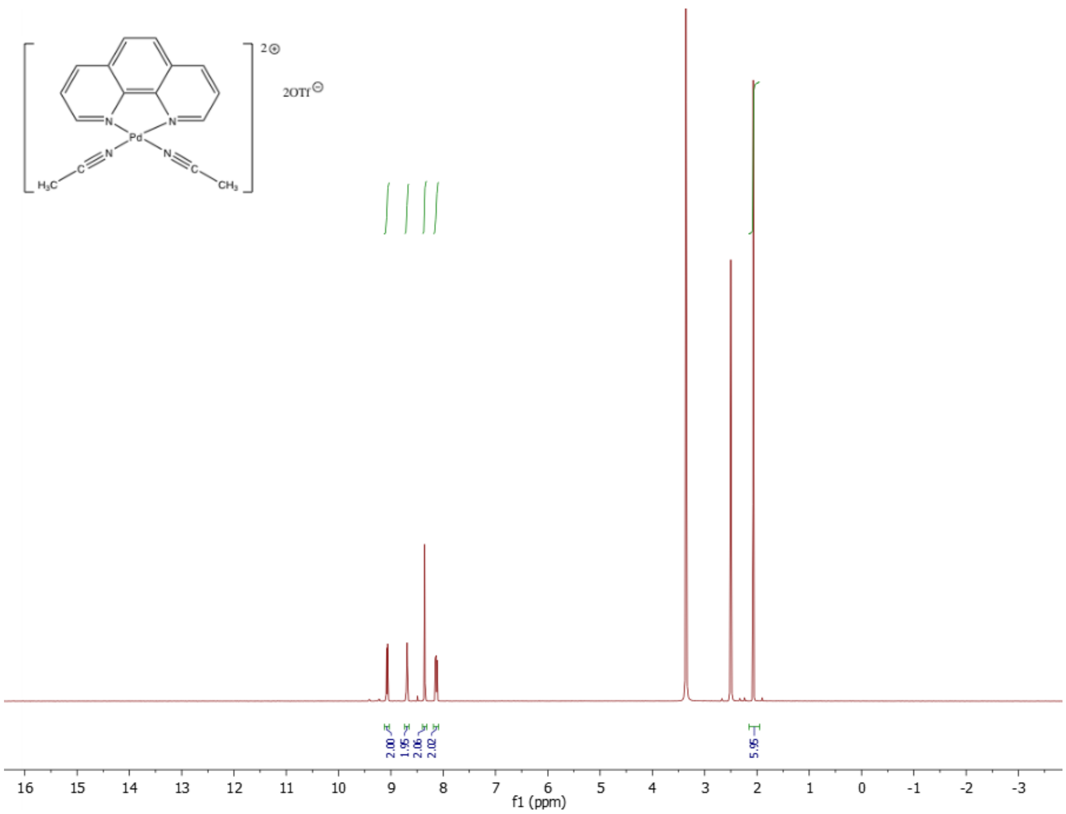
2⊕
2OTf⊖
N
Pd
N
H3C
CH3
2.00
1.95
2.06
2.02
5.95
16
15
14
13
12
11
10
9
8
7
6
5
4
3
2
1
0
-1
-2
-3
f1 (ppm)

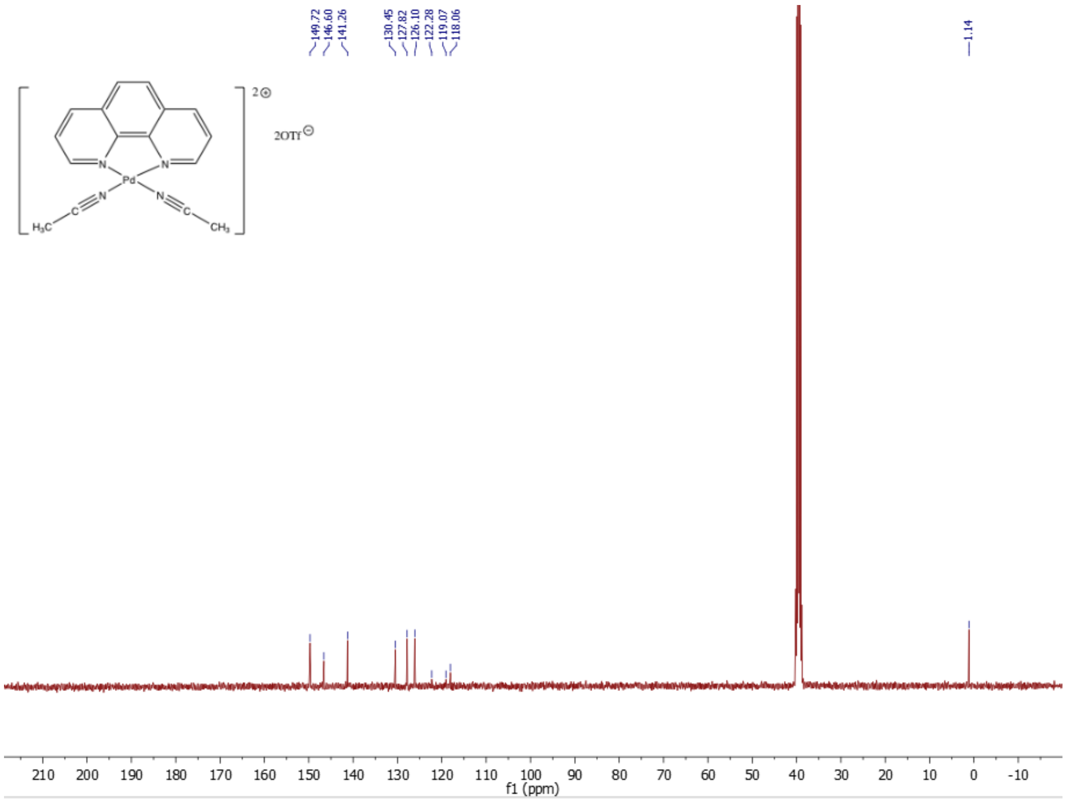
149.72
146.60
141.26
130.45
127.82
126.10
122.28
119.07
118.06
1.14
2⊕
2OTf⊖
N
Pd
N
H3C
CH3
210
200
190
180
170
160
150
140
130
120
110
100
90
80
70
60
50
40
30
20
10
0
-10
f1 (ppm)

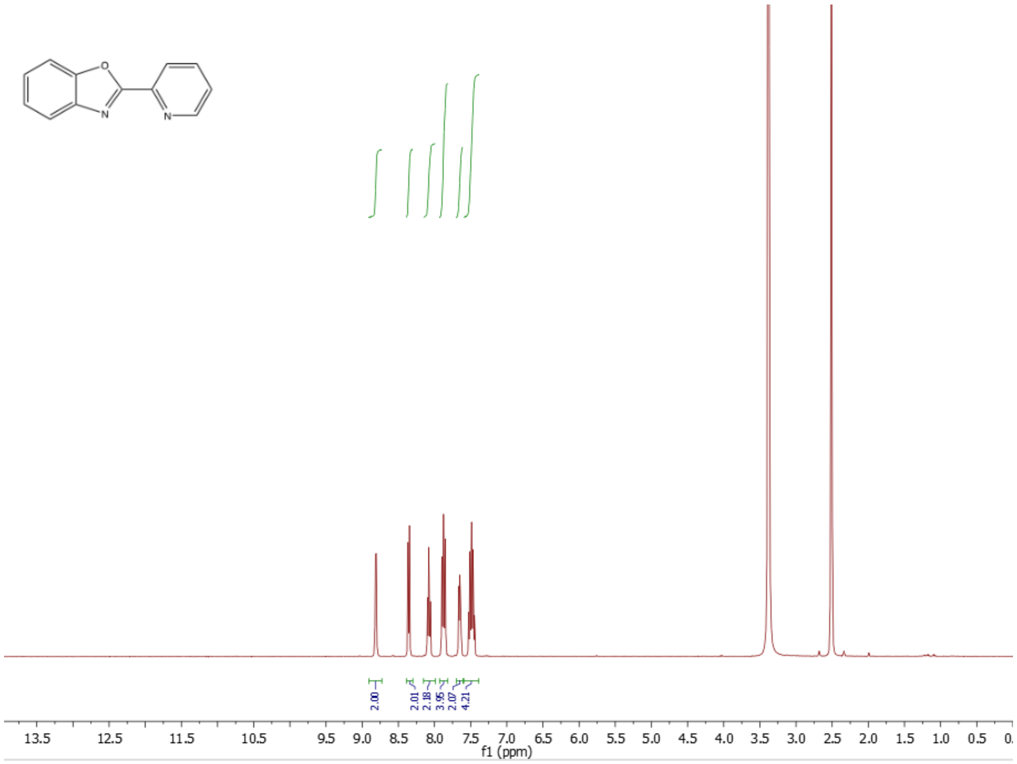
O
N
N
2.00
2.01
2.18
3.95
2.07
4.21
13.5 12.5 11.5 10.5 9.5 9.0 8.5 8.0 7.5 7.0 6.5 6.0 5.5 5.0 4.5 4.0 3.5 3.0 2.5 2.0 1.5 1.0 0.5 0.
f1 (ppm)

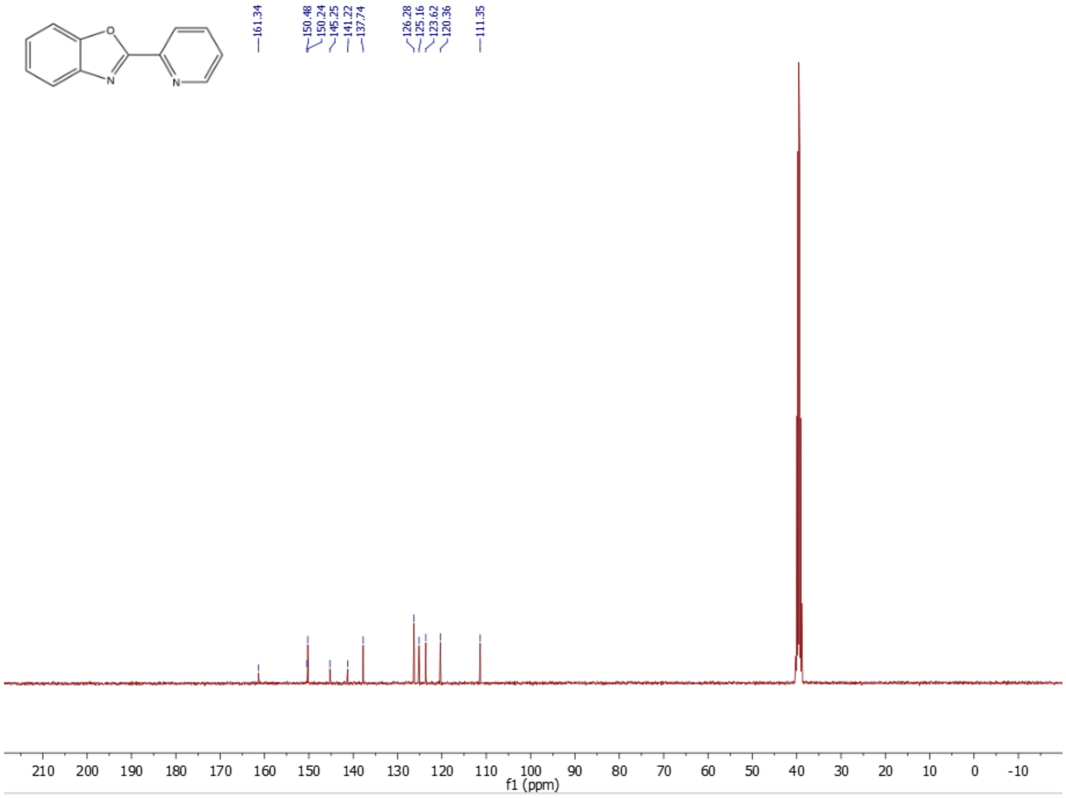
O
N
N
161.34
150.48
150.24
145.25
141.22
137.74
126.28
125.16
123.62
120.36
111.35
210 200 190 180 170 160 150 140 130 120 110 100 90 80 70 60 50 40 30 20 10 0 -10
f1 (ppm)

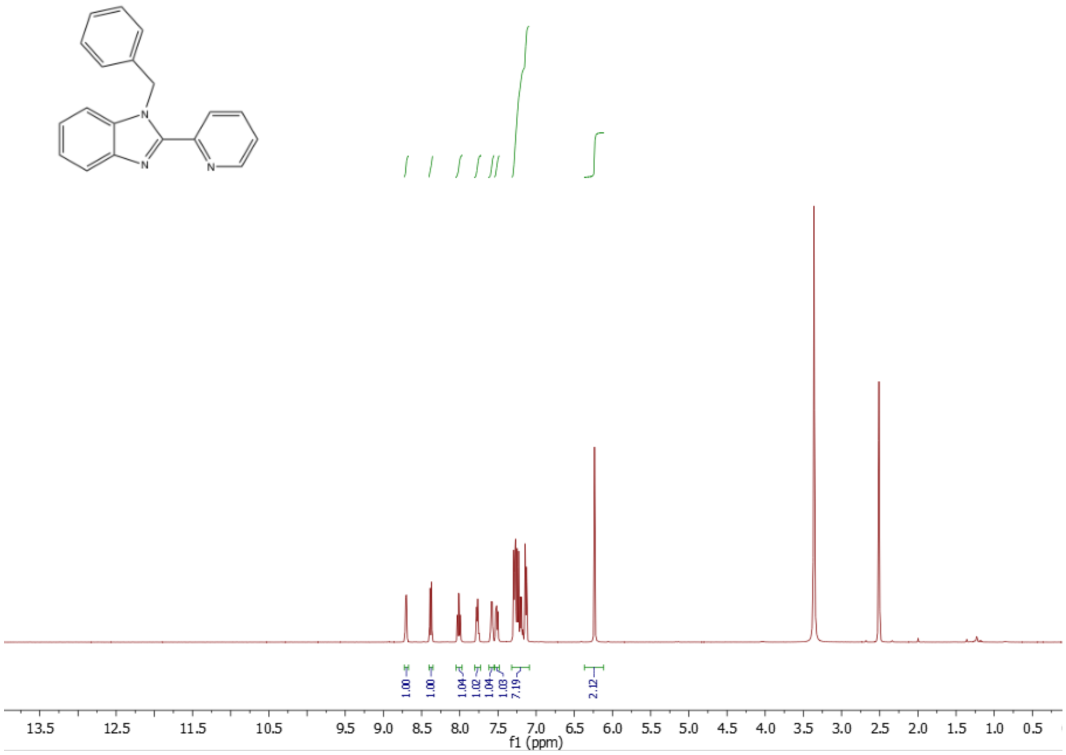

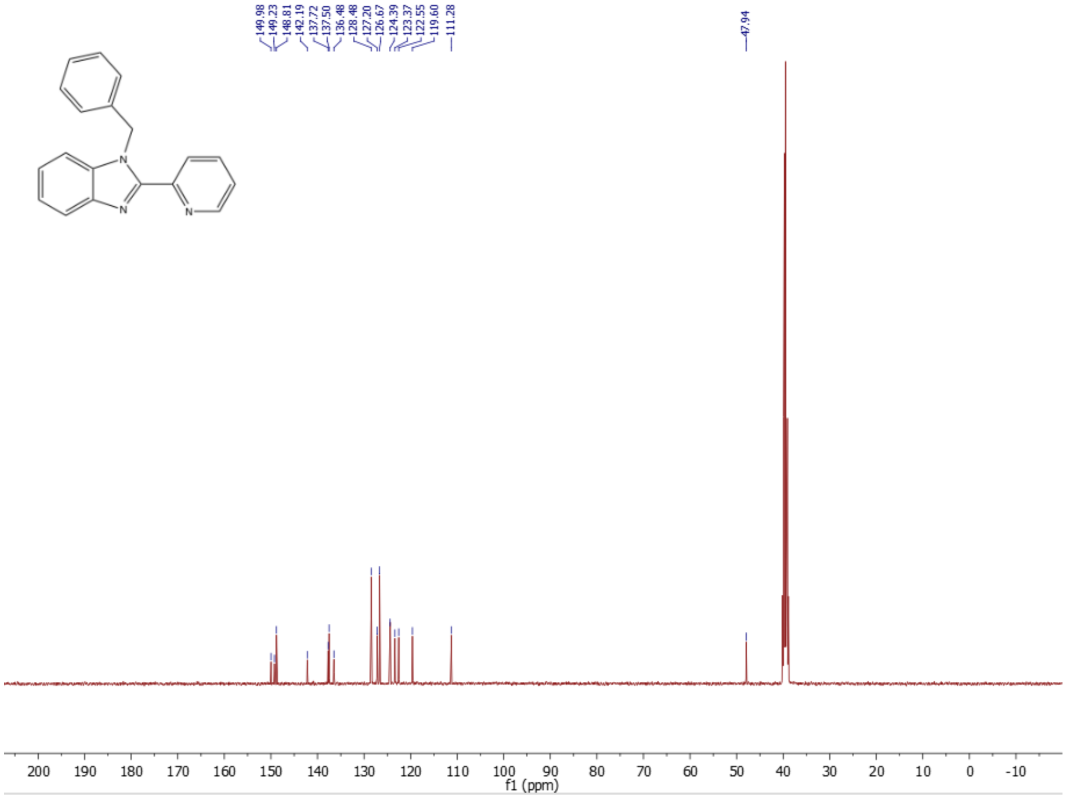

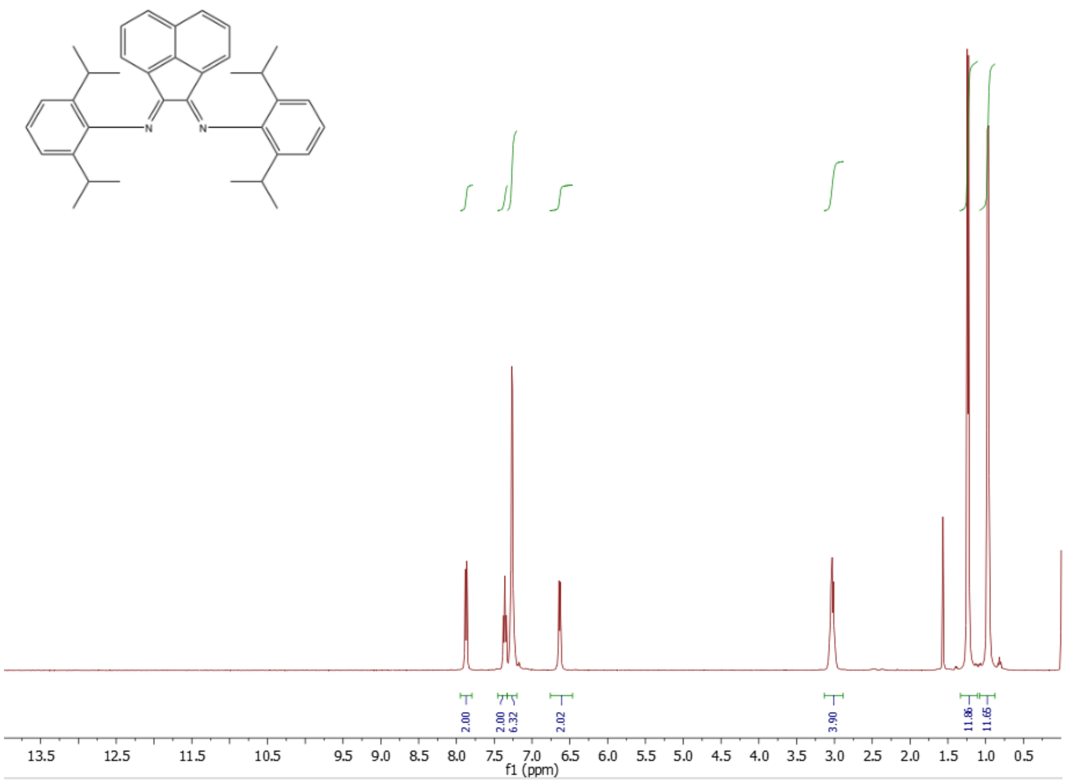
N
N
2.00
2.00
6.32
2.02
3.90
11.86
11.65
13.5 12.5 11.5 10.5 9.5 9.0 8.5 8.0 7.5 7.0 6.5 6.0 5.5 5.0 4.5 4.0 3.5 3.0 2.5 2.0 1.5 1.0 0.5
f1 (ppm)

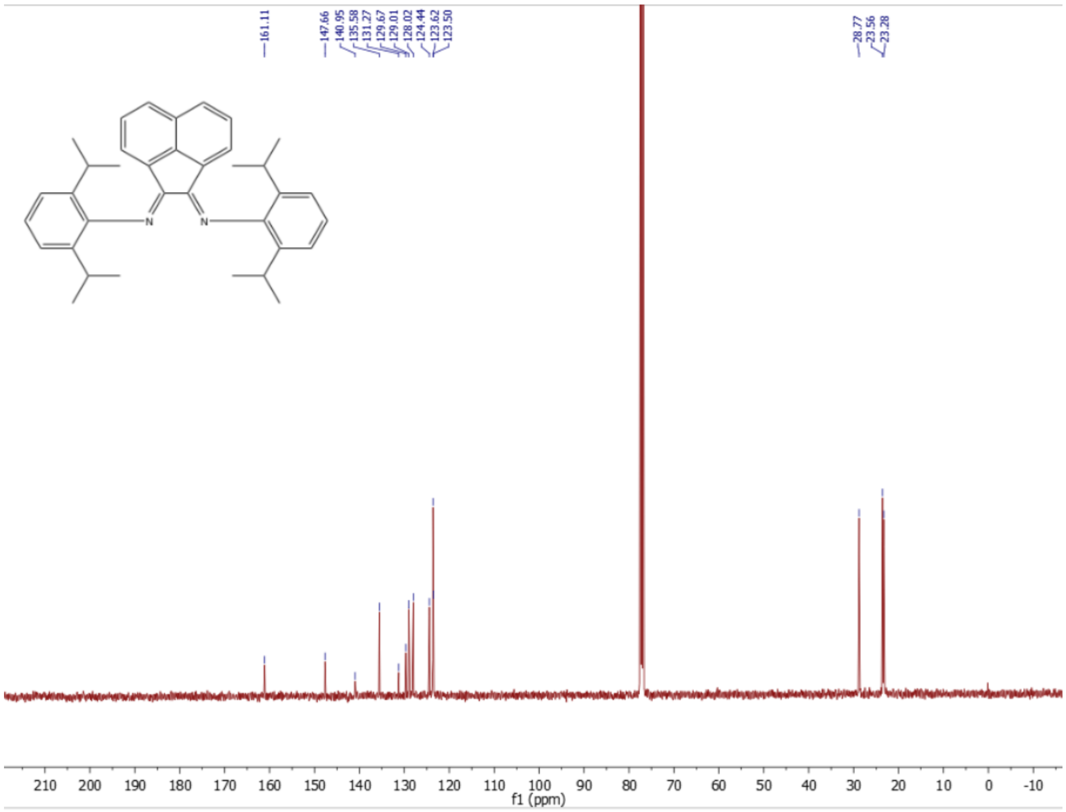
161.11
147.66
140.95
135.58
131.27
129.67
129.01
128.02
124.44
123.62
123.50
28.77
23.56
23.28
N
N
210 200 190 180 170 160 150 140 130 120 110 100 90 80 70 60 50 40 30 20 10 0 -10
f1 (ppm)

8 References

[1] J. Tummatorn, P. A. Albiniak, G. B. Dudley, *J. Org. Chem.*, **2007**, *72*, 8962.

[2] K. Inamoto, N. Asano, K. Kobayashi, M. Yonemoto, Y. Kondo, *Org. Biomol. Chem.*, **2012**, *10*, 1514.

[3] X. Zhang, W.-Z. Zhang, L.-L. Shi, C. Zhu, J.-L. Jiang, X.-B. Lu, *Tetrahedron*, **2012**, *68*, 9085.

[4] W.-Z. Zhang, W.-J. Li, X. Zhang, H. Zhou, X.-B. Lu, *Org. Lett.*, **2010**, *12*, 4748.

[5] J. Mao, X. Yang, H. Yan, Y. He, Y. Li, J. Zhao, *Catal. Lett*., **2016**, 1.

[6] A. Sakakura, S. Nakagawa and K. Ishihara, *Tetrahedron*, **2006**, *62*, 422.

[7] C. K.-W. Kwong, M. Y. Fu, H. C.-H. Law, P. H. Toy, *Synlett*, **2010**, *17*, 2617.

[8] A. Adhikari, J. Shah, K. Howard, C. Russo, D. Wallach, M. Linaburg and J. Chisholm, *Synlett*, **2014**, *25*, 283.

[9] R. Shen, K. Chen, Q. Deng, J. Yang, L. Zhang, *Org. Lett.*, **2014**, *16*, 1208.

[10] J. Lam, Y. Dong, K. Cheuk and B. Z. Tang, *Macromolecules*, **2003,** *36*, 7927.

[11] G.-J. T. Brink, I. W. C. E. Arends, M. Hoogenraad, G. Verspui, R. A. Sheldon, *Adv. Synth. Catal.*, **2003**, *345*, 1341.

[12] P. Xie, Y. Xie, B. Qian, H. Zhou, C. Xia, H. Huang, *J. Am. Chem. Soc.*, **2012**, *134*, 9902.

[13] B. Milani, E. Alessio, G. Mestroni, A. Sommazzi, F. Garbassi, E. Zangrando, N. Bresciani-Pahor, L. Randaccio, *J. Chem. Soc., Dalton Trans.*, **1994**, 1903.

[14] M. Aresta, P. Giannoccaro, I. Tommasi, A. Dibenedetto, A. M. Manotti Lanfredi, F. Ugozzoli, *Organometallics*, **2000**, *19*, 3879.

[15] J. C. Slootweg, P. Chen, *Organometallics*, **2006**, *25*, 5863.

[16] Q. Cao, D. S. Bailie, R. Fu and M. J. Muldoon, *Green Chem.*, **2015**, *17*, 2750.

[17] Y. Kawashita, N. Nakamichi, H. Kawabata, M. Hayashi, *Org. Lett.*, **2003**, *5*, 3713.

[18] S. Haneda, Z. Gan, K. Eda, M. Hayashi, *Organometallics*, **2007**, *26*, 6551.

[19] W.-K. Huang, C.-W. Cheng, S.-M. Chang, Y.-P. Lee, E. W.-G. Diau, *Chem. Commun.*, **2010**, *46*, 8992.

[20] T. Tu, Z. Sun, W. Fang, M. Xu, Y. Zhou, *Org. Lett.*, **2012**, *14*, 4250.
